# Supplementary material for: Landscapes of missense variant impact for human superoxide dismutase 1
Source: Am J Hum Genet. 2025 Sep 15;112(10):2295–315. doi: 10.1016/j.ajhg.2025.08.019 (PMC12696502; doi:10.1016/j.ajhg.2025.08.019)
Supplement: Document S1. Figures S1–S15 and Tables S1–S3 [file mmc1.pdf]

The American Journal of Human Genetics, Volume *112*

Supplemental information

Landscapes of missense variant impact for human superoxide dismutase 1

Anna Axakova, Megan Ding, Atina G. Cote, Radha Subramaniam, Vignesh Senguttuvan, Haotian Zhang, Jochen Weile, Samuel V. Douville, Marinella Gebbia, Ammar Al-Chalabi, Alexander Wahl, Jason Reuter, Jessica Hurt, Adele A. Mitchell, Stephanie Fradette, Peter M. Andersen, Warren van Loggerenberg, and Frederick P. Roth

Supplemental Note

Preliminary explorations to find an multiplexed assay of SOD1 gain of function effects

ALS-linked SOD1 variants have been reported to confer a gain of function in that they promote the formation of oligomers[1], with toxicity that correlates with the presence of smaller oligomers rather than larger aggregates[2]. Since SOD1-ALS is a dominant disease, we did not knock out the endogenous *SOD1* in HEK293T cells to better recapitulate the endogenous system. Indeed, one pathomechanism that has been implicated in ALS is the potential for wild-type SOD1 to heterodimerize with SOD1 variants and thereby increase the amount of aggregated SOD1[3,4]. Although we showed that human cells bearing SOD1-GFP fusions could reliably identify variants that decrease protein abundance, examination of these cells by fluorescence microscopy did not reveal GFP puncta indicating protein aggregation for either gain of function variants or WT cells under the growth conditions we used initially. We therefore sought alternative conditions that might sensitize cells to this toxic aggregation phenotype, with the hope that we might then use FACS to detect aggregating variants[5]. Previous reports suggested that cells can be sensitized to yield SOD1 variant toxicity either by SOD1 variant overexpression, or by environmental changes that either disrupt calcium homeostasis, trigger calpain or activate neuronal nitric oxide synthase[6,7].

First, to assess whether HEK293T cells for could be sensitive to SOD1 aggregation via its overexpression, we used transient transfection of plasmids with SOD1-GFP (WT; pathogenic variants p.Ala5Val, p.Gly86Arg, p.Arg116Gly which are known to aggregate) downstream of the CMV promoter. This was performed both in our baseline growth condition and after addition of the cysteine protease inhibitor MG-101 (also called ALLN) that has been reported to reduce the processing of misfolded SOD1 and result in a higher aggregation[7]. In these experiments, ALLN treatment did yield more aggregation for p.Ala5Val, p.Gly86Arg and p.Arg116Gly variants than for WT SOD1, but aggregation was observed in fewer than 1% of all cells (Figure S3A). The weakness of this effect, coupled with the fact that our multiplexing strategy is most effective when each cell expresses only a single variant, led us to abandon this sensitization strategy.

Second, we assessed whether SOD1 was able to aggregate when more stably expressed in the HEK293T cell system alone, or with or without treatment with KNK437, a heat shock protein 70 chaperone inhibitor. HEK293T integrant cells bearing SOD1-GFP for multiple alleles, including WT, known-aggregating variants p.Gly86Arg and p.Leu145Phe, as well as p.Gly130Ser (with an unknown propensity to aggregate) were evaluated for aggregation after 24 and 48 hours. Only for KNK437 (and only after 48 hours) did we observe the aggregating phenotype, and this was observed only in <1% of all cells stably expressing pathogenic variants (Figure S3B). Low penetrance of this cellular phenotype, coupled with variable aggregation across replicates suggested that this assay was unsuitable for a large-scale assay. Additional attempts to increase the sensitivity of this assay to aggregation using combinations of exogenous NO donor (GSNO), calcium ionophore (A23187), sorbitol, heat shock, ALLN and KNK437 were unsuccessful (data not shown).

Supplemental Figures

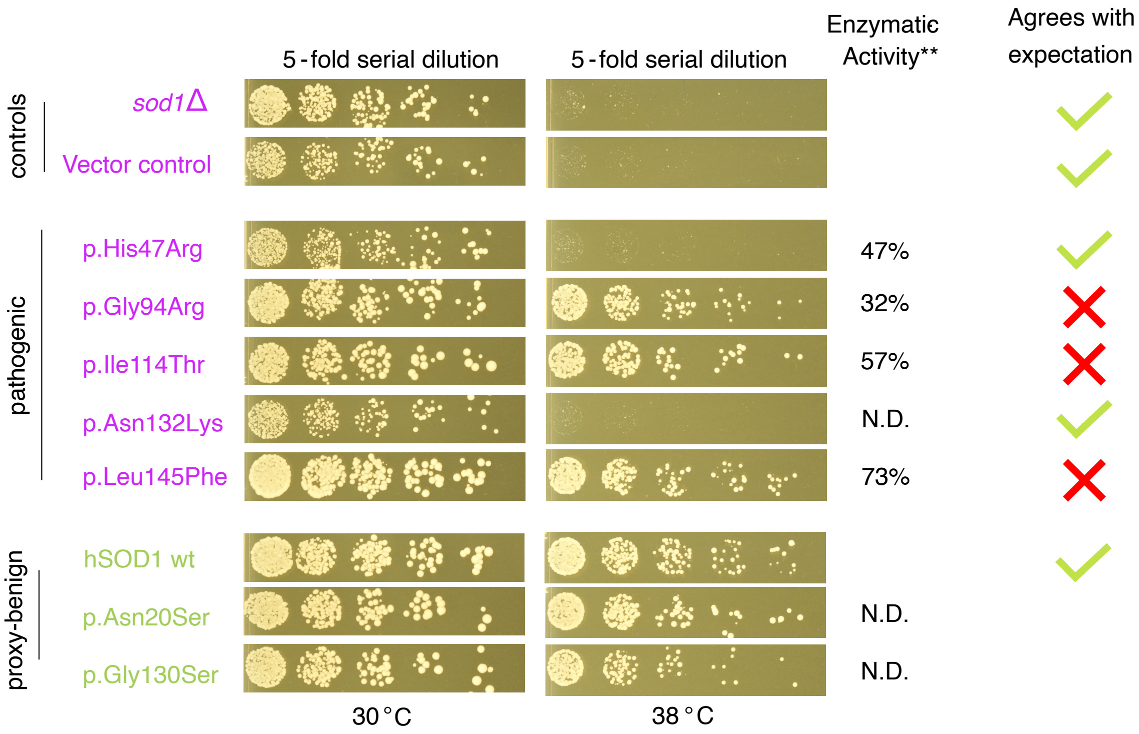

Figure S1: Functional complementation assay results showing whether expression of human SOD1 protein variants can rescue growth of a yeast *sod1Δ* strain.

Complementation assay results for ClinVar-reported negative controls and pathogenic variants (purple text), and WT control and proxy-benign variants (green text). Five-fold serial dilutions of yeast cells were spotted onto plates, with growth evaluation after incubating for 48 hours at either permissive (30°C) or non-permissive (38°C) temperature. Enzymatic activity measurements[8] from individuals with SOD1-ALS variants p.His47Arg, p.Gly94Arg, p.Ile114Thr and p.Leu145Phe are shown.

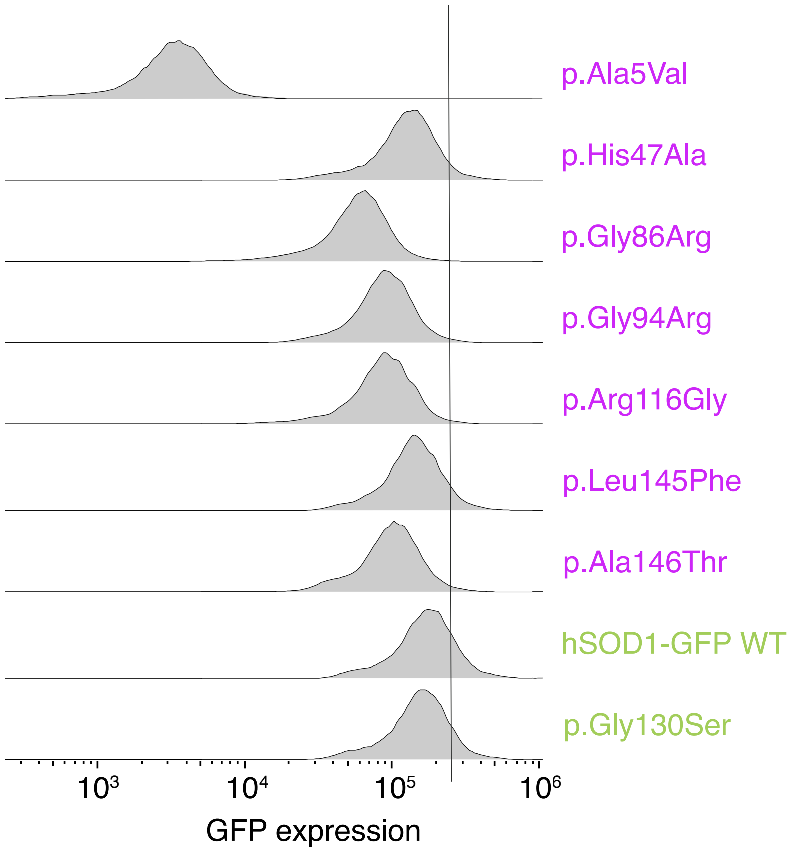

Figure S2: Distributions of GFP intensity from HEK293T cells with stably integrated SOD1-GFP indicate reduced abundance for pathogenic SOD1 variants.
Flow cytometric GFP distribution of single, viable cells with integrated SOD1-GFP at the *Bxb1* site in HEK293T cells for pathogenic variants (purple text), and WT SOD1-GFP control and one proxy-benign variant (p.Gly130Ser) taken from gnomAD (green text). Black vertical line indicates potential gating strategy to enrich for variants with high abundance.

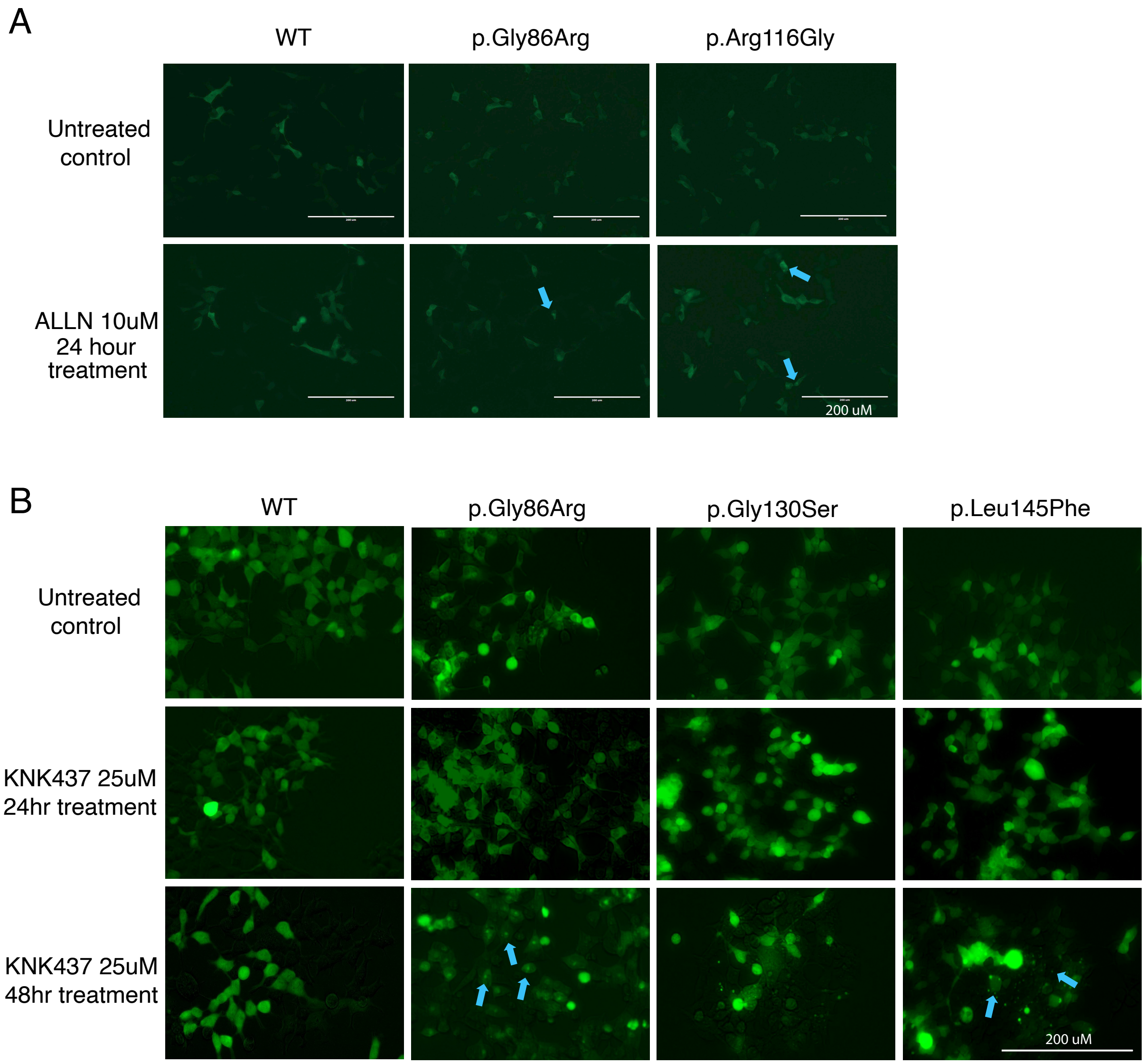

Figure S3: Chemical treatment of HEK293T cells achieved only limited SOD1-GFP aggregation. Aggregates indicated by the blue arrows for: (A) HEK293T cells with SOD1-GFP expressed at high levels via transient transfection, with and without ALLN treatment; (B) HEK293T cells with SOD1-GFP expressed at moderate levels via stable integration in the Bxb1 recombination site, with and without KNK437 treatment.

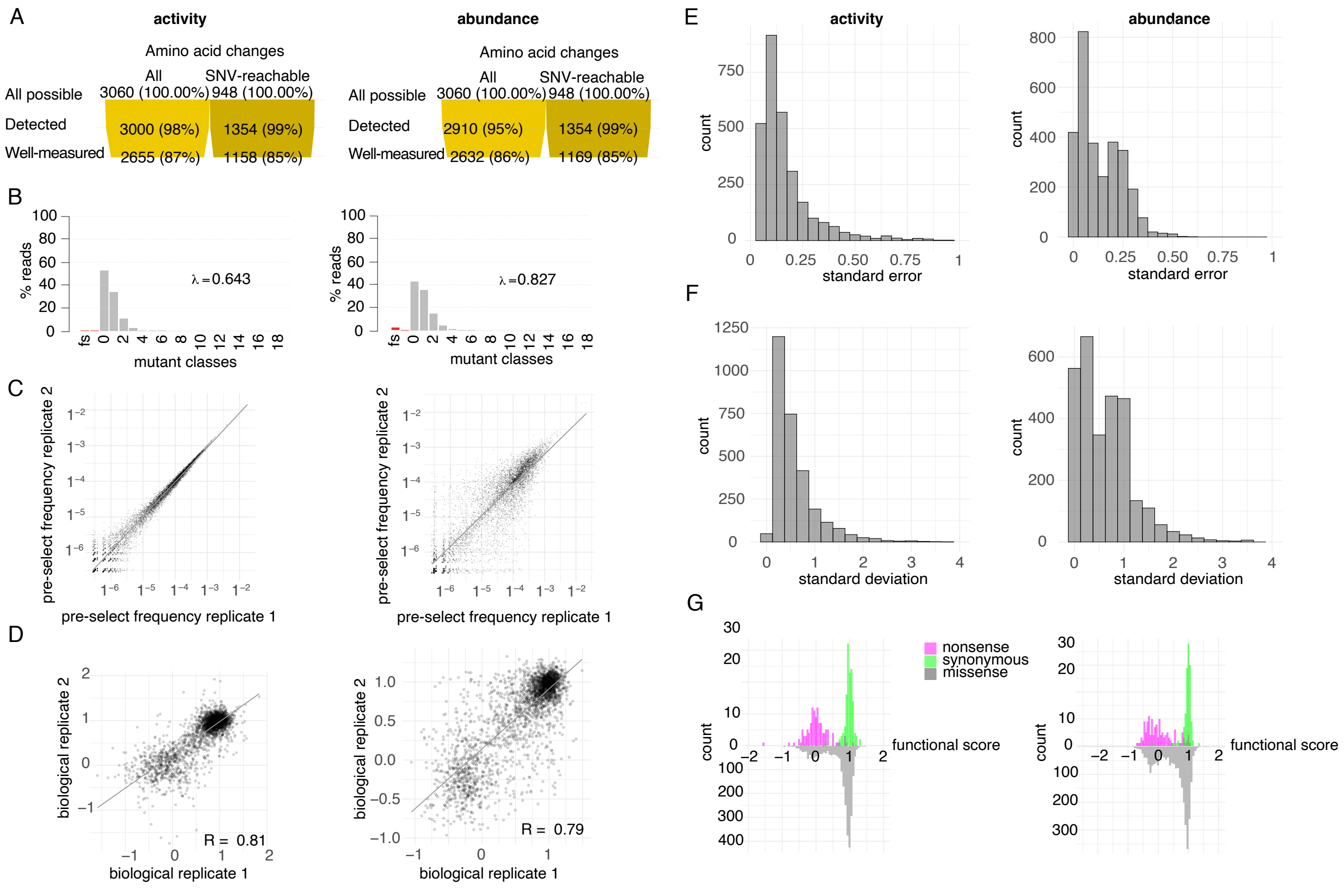

Figure S4: Characterization of SOD1 variant libraries used for activity (left) and abundance (right) variant effect maps.

(A) Percentage of variants detected and passing quality control are shown for each SOD1 map. For each map, the number of synonymous, nonsense, and missense substitutions are shown across all residue positions, both before (left) and after (right) restricting to substitutions that are possible given a single nucleotide change. The three rows correspond to: 1) theoretically possible substitutions; 2) substitutions detected in the pre-selection condition; and 3) substitutions above a threshold pre-selection frequency.

(B) Distribution of the number of missense variants in clones from the total enzymatic activity and abundance SOD1 mutagenized libraries, and the fraction of clones carrying small indels resulting in frameshifts (“fs”). The average number of amino acid changes per clone (λ) is also estimated (see Material and Methods).

(C) Pre-selection variant frequency correlation between biological replicates, for each assay.

(D) Functional score (including missense, nonsense and synonymous variants) correlations between biological replicates, for each assay.

(E) Distributions of standard errors for all missense, nonsense and synonymous variants measured.

(F) Distributions of standard deviations for all missense, nonsense and synonymous variants measured.

(G) Distributions of measured functional impact scores for nonsense (pink), synonymous (green), and missense (gray) variants for the maps.

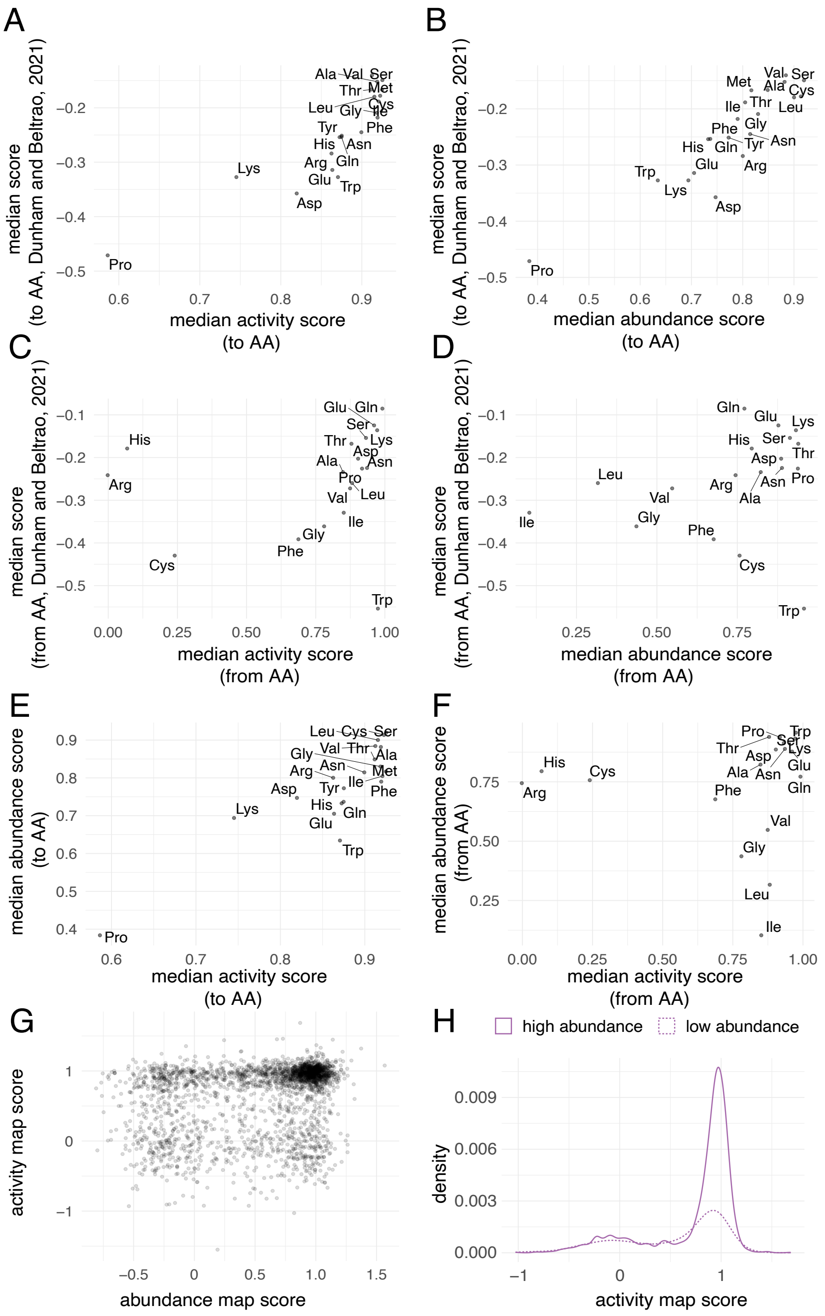

Figure S5: Correspondence between functional scores of SOD1 missense variants from the total enzymatic activity and abundance maps.

Correlation of activity and abundance map functional scores to aggregated scores from substitutions ‘to’ (A-B) and ‘from’ (C-D) amino acids from 28 deep mutational scans[9]. (‘To’ amino acids: Spearman’s R activity = 0.86, p<2e-16. Spearman’s R abundance = 0.90, p<2e-6. ‘From’ amino acids: Spearman’s R activity = 0.48, p=0.045, abundance p>0.05). Correlation of scores corresponding to median score of changes to (E) and from (F) specific amino acids between abundance and activity map (Spearman’s R to amino acid = 0.80, p<4e-5; Spearman’s R from amino acid = 0.56, p<0.02). Correlation between activity and abundance map scores for missense variants only (G; Spearman’s R = 0.25; p<2e-16). Activity scores corresponding to variants with high and low-abundance functional scores (H; abundance score > 0.5, abundance score < 0.5, respectively.) Solid lines represent linear regression fits.

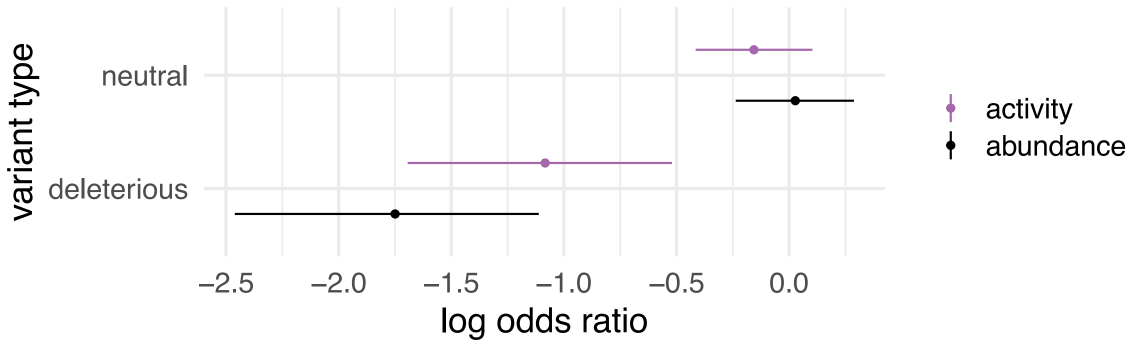

Figure S6: Depletion in human cohorts of missense variants with damaging functional impact scores for activity and abundance assays. Log-odds ratios for the depletion of variants with neutral or damaging scores from total enzymatic activity (purple) or abundance (black) maps in both UK Biobank and gnomAD population sequencing databases. Range lines represent 95% confidence intervals.

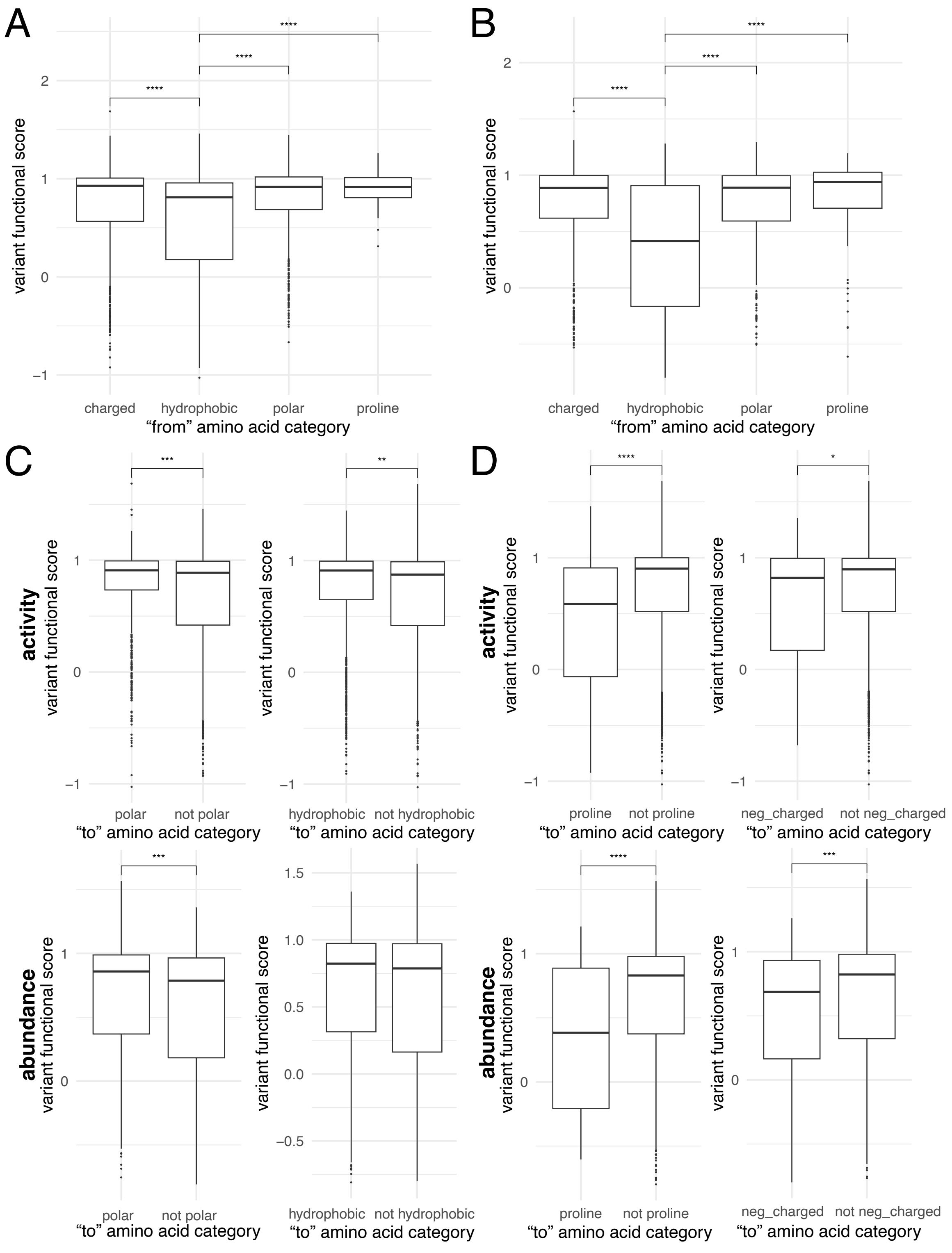

Figure S7: Variant functional scores by biochemical property.
Variant functional scores by initial amino acid category for the (A) activity map and (B) the abundance map. Scores for conservative substitutions compared to all else from (C) initially polar positions and initially hydrophobic positions for activity (top) and abundance maps (bottom). (D) Scores for variants changed to proline, or to negatively charged residues (Asp or Glu) for activity (top) and abundance maps (bottom). $p^{**}<0.01$, $p^{***}<0.001$, $p^{****}<0.0001$ by Wilcoxon.

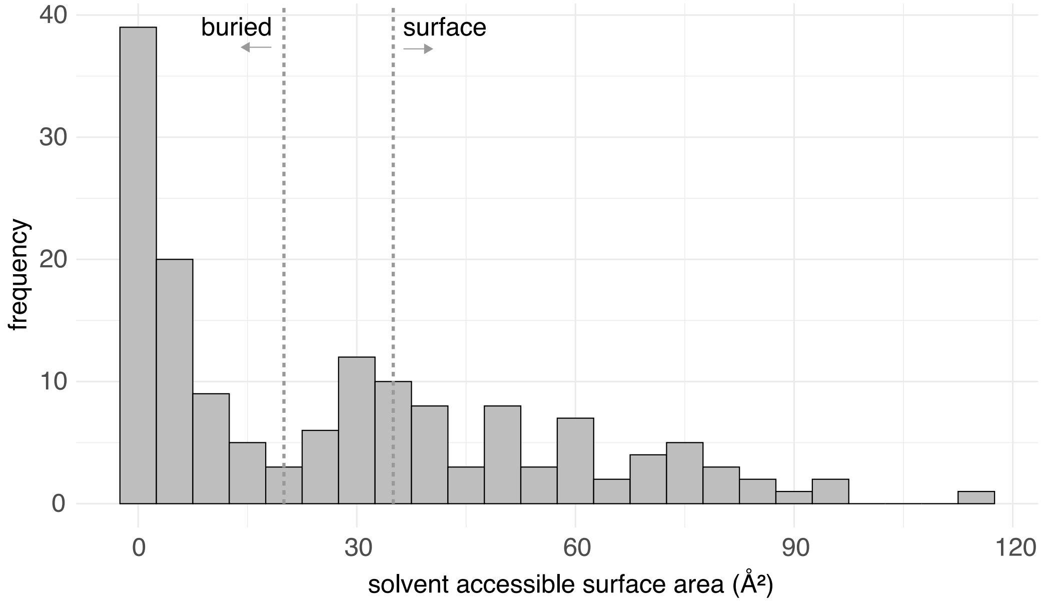

Figure S8: Distribution of solvent accessible surface area for SOD1 residues. SOD1 residues with surface area values exceeding 35% (based on freeSASA values) were considered exposed, while those below 20% were classified as buried (see Materials and Methods).

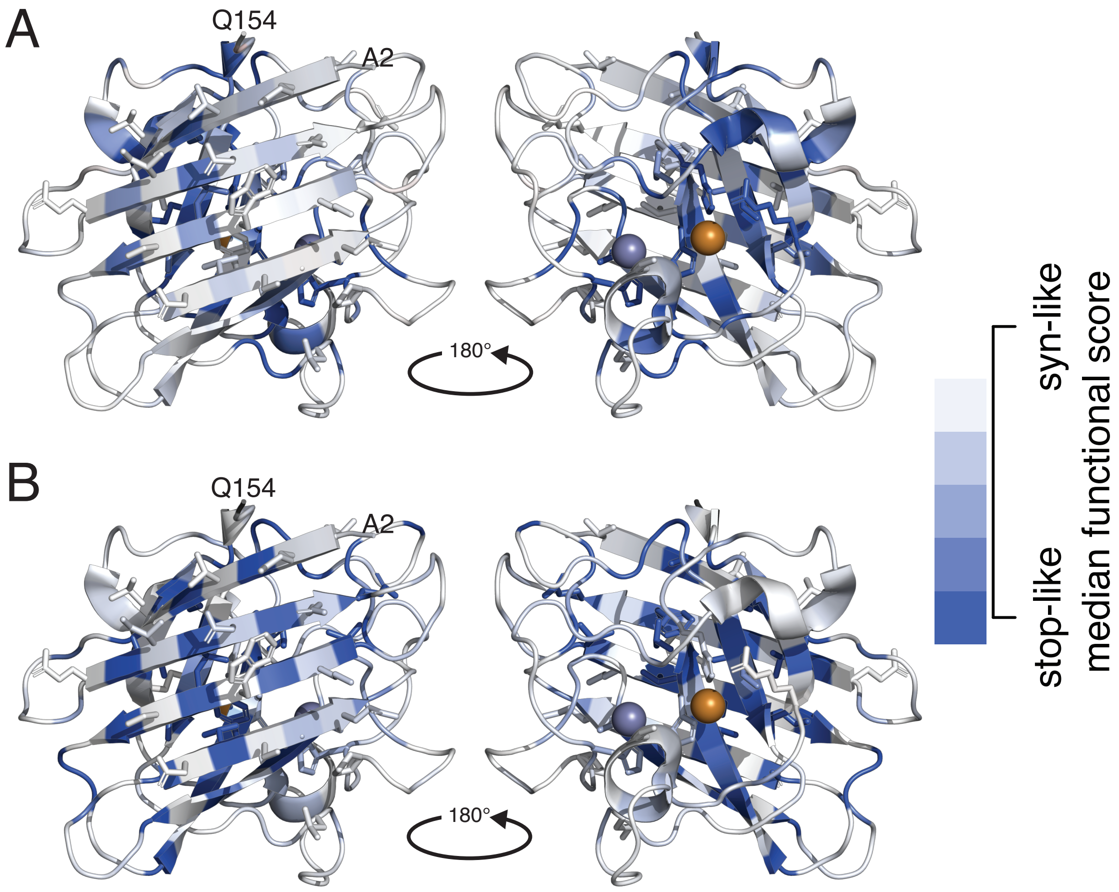

Figure S9: Crystal structure for SOD1 with residues colored according to map scores. Residues in the structure, PDB:1HL5[10] with Cu^{2+} (orange)/Zn^{2+} (blue-grey) ions, were colored based on the median scores of missense variants measured from (A) the enzymatic activity map and (B) the abundance map.

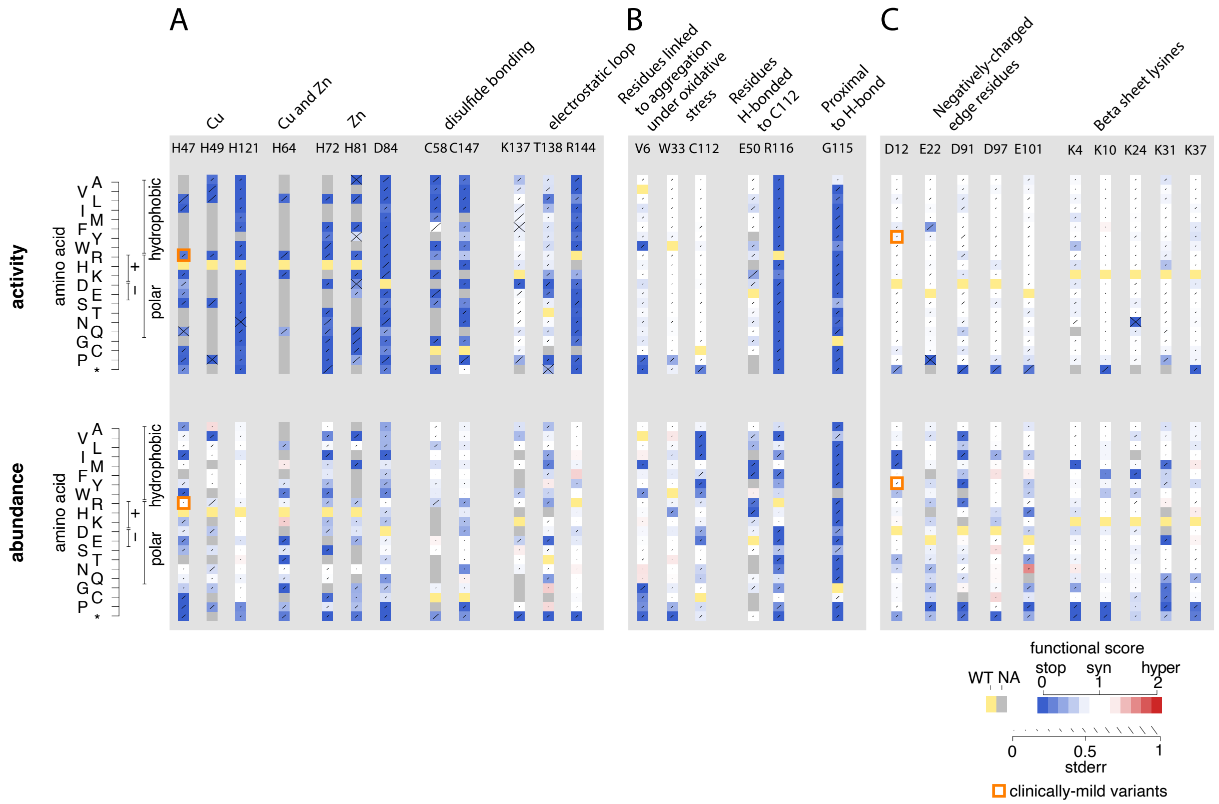

Figure S10: Identifying patterns of mutational tolerance in SOD1.

Functional scores for each possible amino acid substitution (y-axis) at specific SOD1 residue position sets of interest (x-axis). Sets of interest included: (A) residues that participate in Cu2+ and Zn2+-binding, disulphide bonds, or the electrostatic loop that promotes high-affinity site-specific metal binding; (B) residues at which variation has been reported to either cause SOD1 aggregation under oxidative stress, or instability of the H-bond around Cys112; and (C) negatively-charged residues at the edge of the first β-sheet and lysine residues within the first β-sheet. Orange boxes indicate variants with clinically-mild disease presentation.

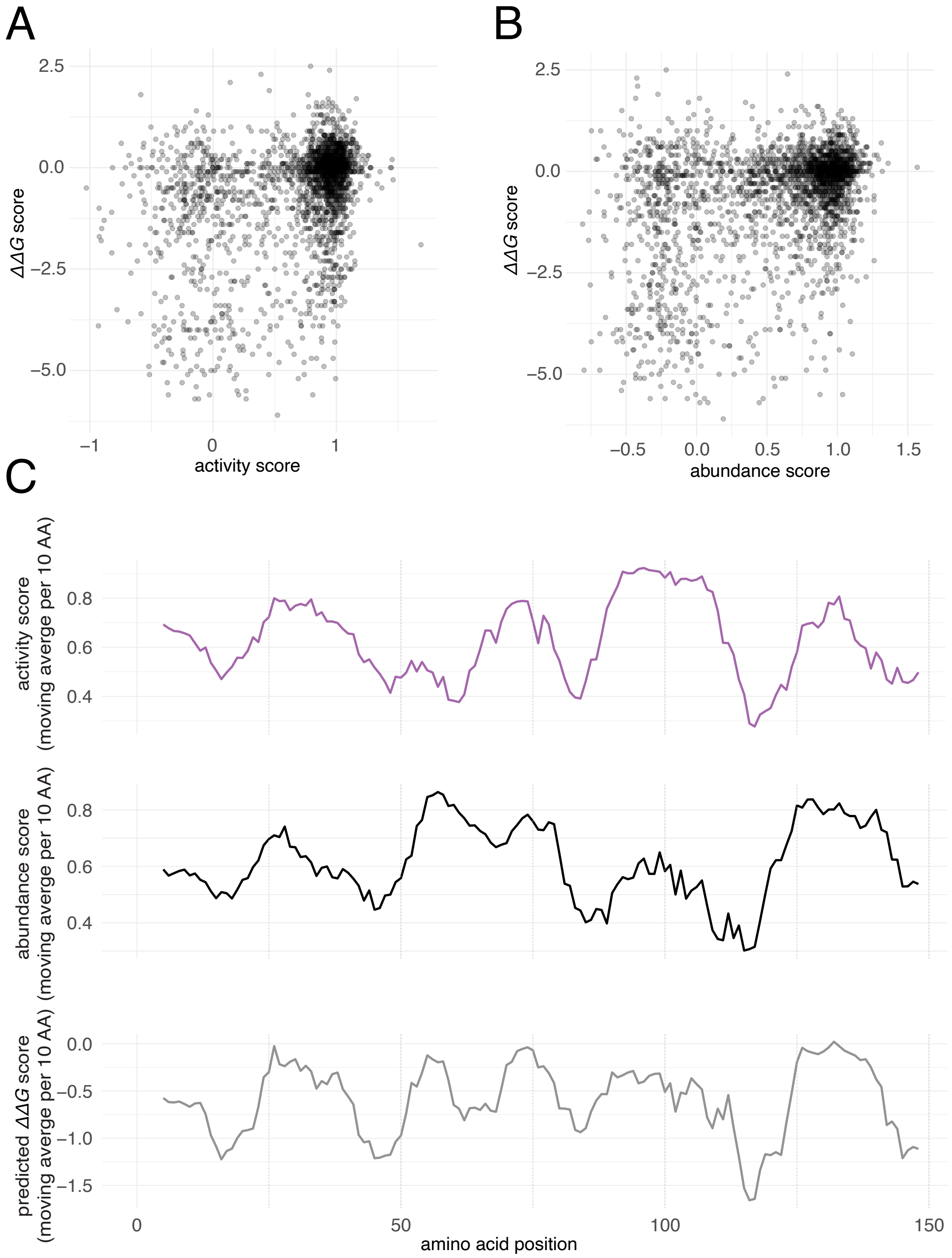

Figure S11: Comparing map scores with predicted effects on protein stability. Functional scores compared to free energy change (*ΔΔG*) for activity (A) and abundance (B) maps. (C) Moving windows of scores from the total enzymatic activity (purple), abundance maps (black), and predicted ΔΔG (light grey) values for SOD1 missense variants at different protein positions. Plotted values represent averages within windows of ten amino acid (AA) positions.

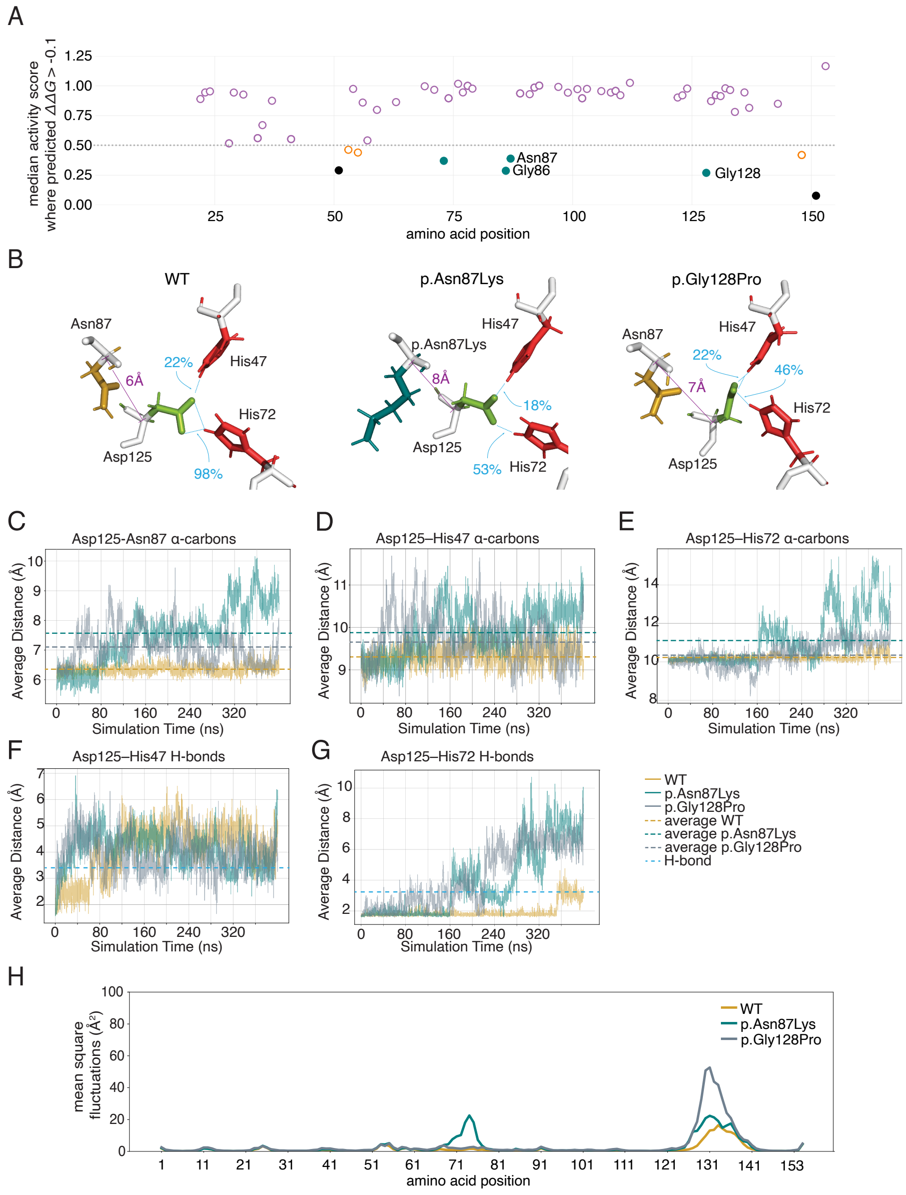

Figure S12: Modeling effects of SOD1 missense variants on protein stability and structure.
(A) For the subset of SOD1 missense variants with no predicted detrimental impact on stability (median predicted *ΔΔG*>-0.1), median total enzymatic activity score is plotted. Circles indicate residues with damaging activity scores: a) at the SOD1-CCS interface (orange); b) residues proximal to the active site ('second-shell residues'; teal); c) at the SOD1 homodimeric interface (black).
(B) To illustrate the structural impact of variants p.Asn87Lys and p.Gly128Pro on the metal-binding site, the average distance (Å) between the Cα atoms of residue pairs 125 (electrostatic loop) and 87 (βstrand residue proximal to the electrostatic loop) is shown in purple, along with the fraction of simulation time (blue) in which hydrogen bonds occurred between metal-binding residue pairs Asp125-His47 and Asp125-His72.
(C-G) Molecular dynamics simulations evaluating impact of variation on inter-residue distances relevant for SOD1's ability to bind metal. The dotted lines represent the average distances between the α-carbons of residue 125 and (C) Asn87, (D) His47, and (E) His72, in WT SOD1, as well as in the p.Asn87Lys and p.Gly128Pro variants. The blue dotted line indicates the hydrogen bond interaction threshold (3.4 Å) for (F) Asp125-His47 and (G) Asp125-His72.
(H) Mean-square fluctuation (MSF) of Cα atoms of WT SOD1, as well as p.Asn87Lys and p.Gly128Pro variants. MSF reflects the average deviation of atoms throughout the simulation (400 ns) relative to the initial structure.

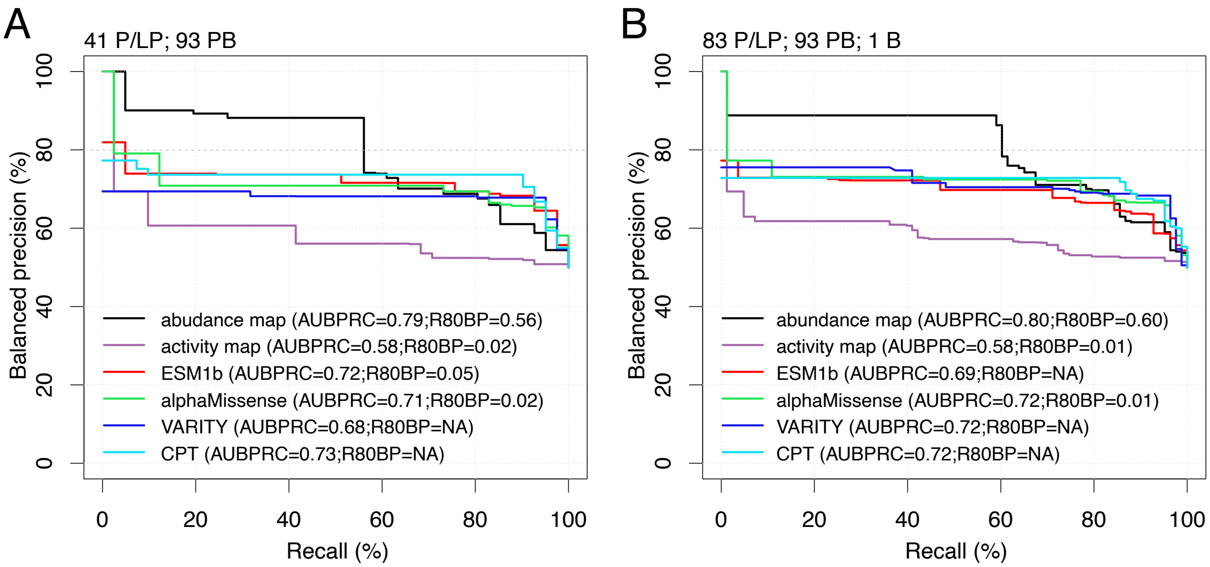

Figure S13: Comparison of total enzymatic activity and abundance SOD1 maps with computational predictors using balanced precision recall analysis for SOD1. We used reference sets drawn from (A) ClinVar/gnomAD and (B) Labcorp/gnomAD to draw PRC curves for the maps and computational predictors. Here we evaluate precision (fraction of variants scoring below each threshold functional impact score that are in the positive reference set containing pathogenic variants) vs recall (fraction of positive reference variants with functional scores below threshold). Precision has been transformed to reflect performance in a balanced test setting where positive and negative sets contain the same number of variants. Balanced precision-recall curves are shown for the total enzymatic activity (purple) and abundance maps (black), as well as computational predictors; ESM1b (red), AlphaMissense (green), VARITY (blue) and CPT (turquoise). Positive and negative reference set sizes (P/LP and B/PB, respectively; see Methods) are indicated. The number of variants in the reference set included in these PRC curves was lower than the curated reference sets due to the intersection between sets for experimental and computational approaches. The variant p.Asn20Ser was classified as B by Labcorp Genetics but was excluded from the ClinVar set because of a conflicting annotation.

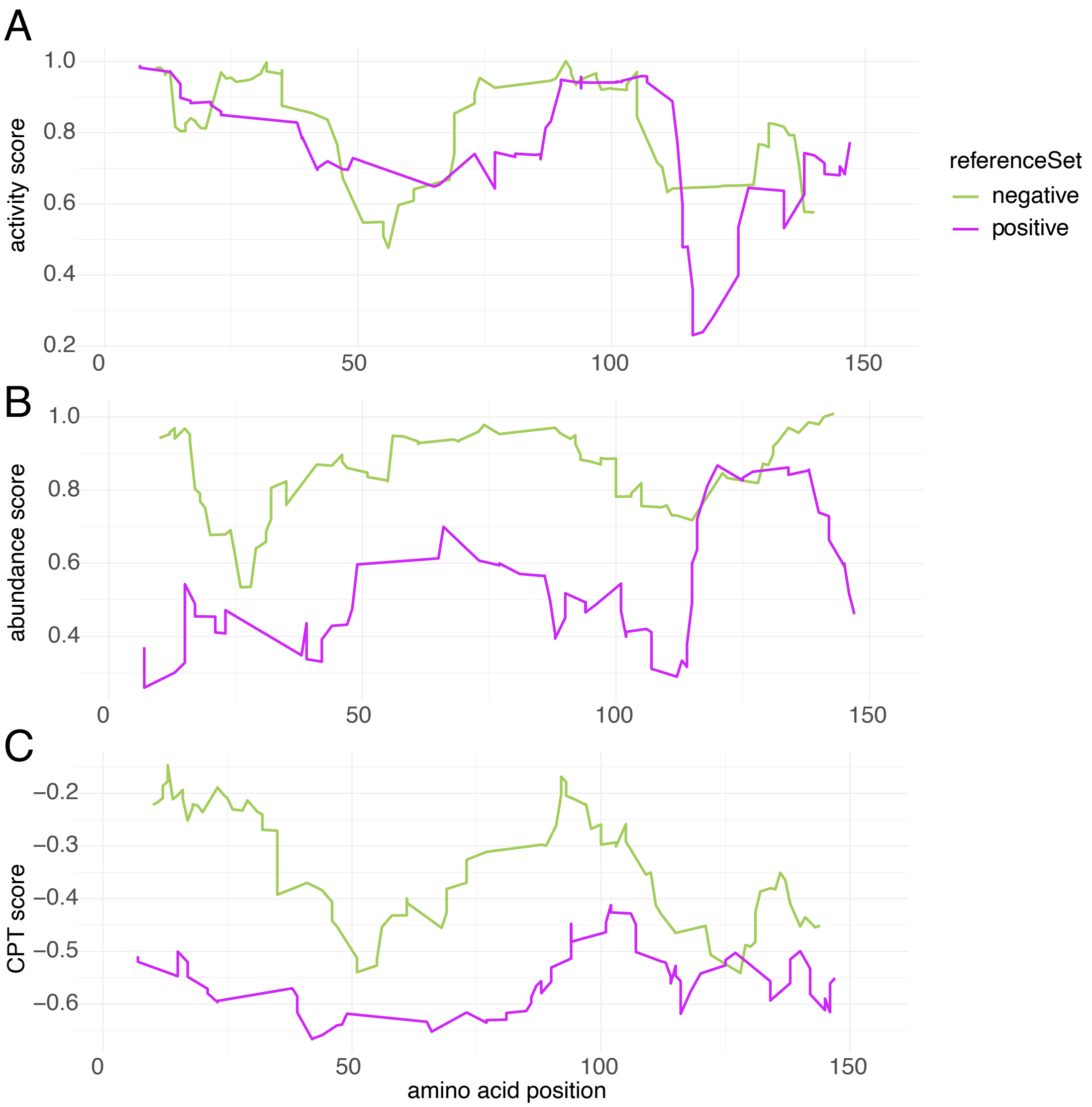

Figure S14: Correspondence of SOD1 maps and computational predictor CPT to clinical variant annotations. Plotted values are (A) activity map, (B) abundance map and (C) computational predictor CPT scores as part of the positive (magenta) or negative (green) reference variant sets from Labcorp/gnomAD, respectively. CPT scores were multiplied by -1 so score directions matched the map scores (more negative considered more damaging).

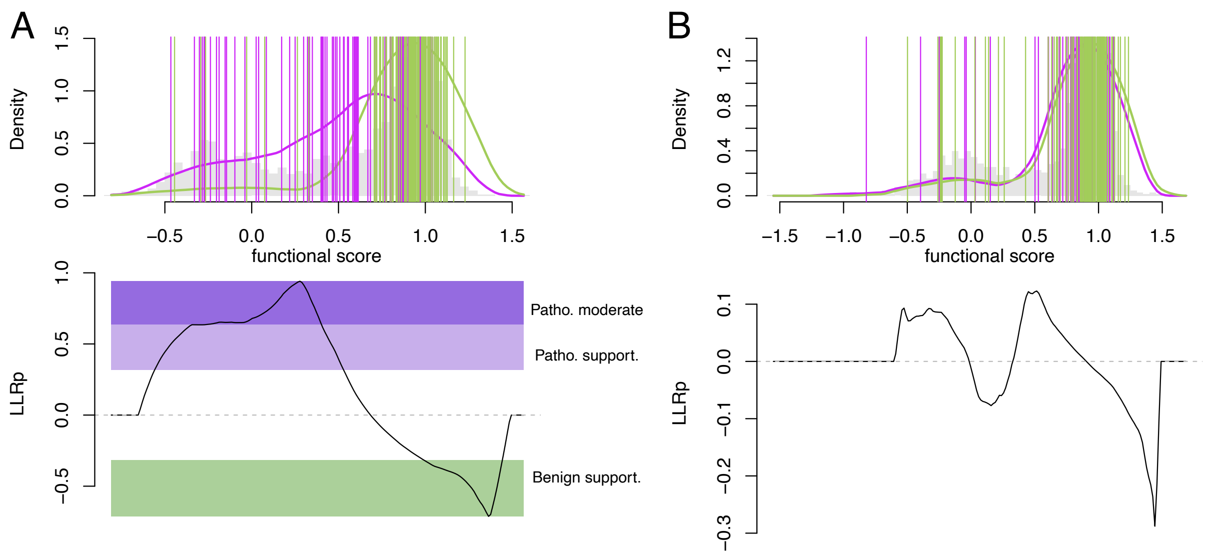

Figure S15: Evidentiary value of abundance map scores for clinical variant interpretation. Calculation and distribution of log-likelihood ratios of pathogenicity (LLRps) for scores of the SOD1 abundance (A) and activity (B) maps. The functions (top) express the log ratio between the likelihood of observing a given score in the score distribution of the Labcorp/gnomAD reference set, including positive reference variants (magenta) from Labcorp Genetics and negative reference set variants from gnomAD + one B from Labcorp (green). LLRps and corresponding evidence strengths are indicated in the bottom panels. Gray histogram bars show the distribution of missense variants for comparison.

Table S1: Summary table of molecular dynamics simulations for residues involved in SOD1 electrostatic loop.

Condition	Position 1	Position 2	Ca Distance (A) between positions 1 and 2	Hydrogen Bonding Time (%) between positions 1 and 2
WT	Asn87	Asp125	6.4 ± 0.2	N/A
WT	His47	Asp125	9.3 ± 0.3	22
WT	His72	Asp125	10.2 ± 0.2	98
p.Asn87Lys	p.Asn87Lys	Asp125	7.6 ± 1.0	N/A
p.Asn87Lys	His47	Asp125	9.9 ± 0.6	18
p.Asn87Lys	His72	Asp125	11.2 ± 1.4	53
p.Gly128Pro	Asn87	Asp125	7.1 ± 0.5	N/A
p.Gly128Pro	His47	Asp125	9.7 ± 0.5	22
p.Gly128Pro	His72	Asp125	10.4 ± 0.6	46

Table S2: Summary table of variant impact on activity or abundance assays for measured variants included in either reference set, with variant-specific information detailed in Table S1.

	**ClinVar/gnomAD Reference Set**		**Labcorp/gnomAD Reference Set**	
	ClinVar P/LP	gnomAD PB	Labcorp P/LP	gnomAD PB, 1 Labcorp B
% damaging (score <0.5) in activity map	5/41<br>12%	12/95<br>13%	10/84<br>12%	12/96<br>13%
% damaging (score <0.5) in abundance map	19/41<br>46%	7/96<br>7%	34/83<br>41%	7/96<br>7%

Table S3: Summary table of the area under the balanced precision-recall curve (AUBPRC) and the recall at 80% balanced precision for maps and computational predictors for the two reference sets tested.

	**Reference set**			
**Map or computational predictor**	ClinVar/gnomAD: 41 ClinVar P/LP, 93 gnomAD PB		Labcorp/gnomAD**:** 83 Labcorp P/LP, 93 gnomAD PB, 1 Labcorp B	
	**AUBPRC**	**R80BP**	**AUBPRC**	**R80BP**
Abundance map	0.79	56%	0.80	60%
Activity map	0.58	2%	0.58	1%
ESM1b	0.72	5%	0.69	NA
alphaMissense	0.71	2%	0.72	1%
VARITY	0.68	NA	0.72	NA
CPT	0.73	NA	0.72	NA

Supplemental References

1. Proctor, E.A., Fee, L., Tao, Y., Redler, R.L., Fay, J.M., Zhang, Y., Lv, Z., Mercer, I.P., Deshmukh, M., Lyubchenko, Y.L., et al. (2016). Nonnative SOD1 trimer is toxic to motor neurons in a model of amyotrophic lateral sclerosis. Proc. Natl. Acad. Sci. U. S. A. *113*, 614–619.

2. Choi, E.S., and Dokholyan, N.V. (2021). SOD1 oligomers in amyotrophic lateral sclerosis. Curr. Opin. Struct. Biol. *66*, 225–230.

3. Huai, J., and Zhang, Z. (2019). Structural Properties and Interaction Partners of Familial ALS-Associated SOD1 Mutants. Front. Neurol. *10*, 527.

4. Brasil, A. de A., de Carvalho, M.D.C., Gerhardt, E., Queiroz, D.D., Pereira, M.D., Outeiro, T.F., and Eleutherio, E.C.A. (2019). Characterization of the activity, aggregation, and toxicity of heterodimers of WT and ALS-associated mutant Sod1. Proc. Natl. Acad. Sci. U. S. A. *116*, 25991–26000.

5. Hidalgo, I.H., Fleming, T., Eckstein, V., Herzig, S., Nawroth, P.P., and Tyedmers, J. (2016). Characterization of aggregate load and pattern in living yeast cells by flow cytometry. Biotechniques *61*, 137–148.

6. Kim, H.J., Im, W., Kim, S., Kim, S.H., Sung, J.J., Kim, M., and Lee, K.W. (2007). Calcium-influx increases SOD1 aggregates via nitric oxide in cultured motor neurons. Exp. Mol. Med. *39*, 574–582.

7. Lee, H., Radu, C., Han, J.W., and Grailhe, R. (2017). Assay Development for High Content Quantification of Sod1 Mutant Protein Aggregate Formation in Living Cells. J. Vis. Exp. https://doi.org/10.3791/56425.

8. Huang, M., Liu, Y.U., Yao, X., Qin, D., and Su, H. (2024). Variability in SOD1-associated amyotrophic lateral sclerosis: geographic patterns, clinical heterogeneity, molecular alterations, and therapeutic implications. Transl. Neurodegener. *13*, 28.

9. Dunham, A.S., and Beltrao, P. (2021). Exploring amino acid functions in a deep mutational landscape. Mol. Syst. Biol. *17*, e10305.

10. Strange, R.W., Antonyuk, S., Hough, M.A., Doucette, P.A., Rodriguez, J.A., Hart, P.J., Hayward, L.J., Valentine, J.S., and Hasnain, S.S. (2003). The structure of holo and metal-deficient wild-type human Cu, Zn superoxide dismutase and its relevance to familial amyotrophic lateral sclerosis. J. Mol. Biol. *328*, 877–891.
