# Supplementary material for: Landscapes of missense variant impact for human superoxide dismutase 1
Source: Am J Hum Genet. 2025 Sep 15;112(10):2295–315. doi: 10.1016/j.ajhg.2025.08.019 (PMC12696502; doi:10.1016/j.ajhg.2025.08.019)
Supplement: Document S2. Article plus supplemental information [file mmc3.pdf]

Landscapes of missense variant impact for human superoxide dismutase 1

Authors

Anna Axakova, Megan Ding, Atina G. Cote, ..., Peter M. Andersen, Warren van Loggerenberg, Frederick P. Roth

Correspondence

wav14@pitt.edu (W.v.L.),
fritz@pitt.edu (F.P.R.)

***SOD1* variants cause the motor neuron disease amyotrophic lateral sclerosis. Axakova et al. functionally assay ∼86% of all possible SOD1 missense variants, producing a variant-effect map resource that quantitatively correlates with human phenotypes, yields insights into sequence-structure-function relationships, and provides evidence for interpreting clinical variants.**

Axakova et al., 2025, The American Journal of Human Genetics *112*, 2295–2315
October 2, 2025

© 2025 The Author(s). Published by Elsevier Inc. on behalf of American Society of Human Genetics.
https://doi.org/10.1016/j.ajhg.2025.08.019

CellPress

Landscapes of missense variant impact for human superoxide dismutase 1

Anna Axakova,[1,2,3] Megan Ding,[1,2,3] Atina G. Cote,[1,2,3] Radha Subramaniam,[1,2,3] Vignesh Senguttuvan,[4] Haotian Zhang,[4] Jochen Weile,[1,2,3] Samuel V. Douville,[1,2,3,5] Marinella Gebbia,[1,2,3] Ammar Al-Chalabi,[6] Alexander Wahl,[7] Jason Reuter,[7] Jessica Hurt,[8] Adele A. Mitchell,[8] Stephanie Fradette,[8] Peter M. Andersen,[9] Warren van Loggerenberg,[1,2,3,4,*] and Frederick P. Roth[1,2,3,4,*]

Summary

Amyotrophic lateral sclerosis (ALS) is a progressive motor neuron disease for which important subtypes are caused by variation in superoxide dismutase 1 (*SOD1*). Diagnosis based on *SOD1* sequencing can not only be definitive but can also indicate specific therapies available for *SOD1*-associated ALS (SOD1-ALS). Unfortunately, SOD1-ALS diagnosis is limited by the fact that a substantial fraction (currently 26%) of ClinVar SOD1 missense variants are classified as "variants of uncertain significance" (VUSs). Although functional assays can provide strong evidence for clinical variant interpretation, SOD1 assay validation is challenging given the current incomplete and controversial understanding of SOD1-ALS disease mechanism. Using saturation mutagenesis and multiplexed cell-based assays, we measured the functional impact of over 2,000 SOD1 amino acid substitutions on both enzymatic function and protein abundance. The resulting "missense variant-effect maps" not only reflect prior biochemical knowledge of SOD1 but also provide sequence-structure-function insights. Importantly, our variant-abundance assay can discriminate pathogenic missense variation and provides new evidence for 41% of missense variants that had been previously reported as VUSs, offering the potential to identify additional people who would benefit from therapy approved for SOD1-ALS.

Introduction

Amyotrophic lateral sclerosis (ALS; MIM: 105400) is a heritable mainly autosomal-dominant neurodegenerative disease with a lifetime prevalence of 1 per 300 people.[1–3] *SOD1* encodes the ubiquitously expressed free-radical-scavenging enzyme Cu^{2+}/Zn^{2+} superoxide dismutase 1 (MIM: 147450; EC: 1.15.1.1), variants of which appear in 9%–23% of people with diagnosed familial ALS (fALS) and at least 2%–5% of people with diagnosed sporadic ALS (sALS) depending on the population.[4–7]

Historically, ALS diagnosis is delayed for an average of 1 year after symptom onset.[8,9] However, genetic analysis now offers the potential for earlier diagnosis of ALS or carrier status and can identify subsets of people who may benefit from specific therapeutic interventions.[10,11] Classifying the pathogenicity of rare missense variants remains challenging. Of the 156 missense variants of SOD1 that have been reported in ClinVar, 26% have been categorized as "variants of uncertain significance" (VUSs), and none have been interpreted as likely benign or benign.[12] More broadly, for variants across many disease genes, validated functional assays can provide strong evidentiary value under current American College of Medical Genetics and Genomics and Association for Molecular Pathology (ACMG/AMP) guidelines.[13] Cell-based functional assays, either in cultured human cells or more tractable eukaryotic model systems, like yeast, can be used to detect variant functional impacts.[14]

Small-scale functional assays are resource intensive and typically "reactive," performed only after (and often years after) the first clinical presentation of a variant. In contrast, computational methods can predict the impact of all missense variants "proactively," in advance of the first clinical presentation. While computational predictors are steadily improving, these are better suited to detect pathogenic missense variants that cause loss of function.[15] Currently, there are no published variant interpretation guidelines for *SOD1*, and computational methods, as outlined in the ACMG/AMP recommendations for variant interpretation,[13] provide, at best, supporting evidence. Although recent studies have suggested that greater weight should be afforded to computational predictors of pathogenicity,[16,17] this has not been validated for *SOD1* in particular.

Within the past decade, it has become possible to proactively test missense variants at large scale using massively multiplexed cell-based assays, yielding exhaustive sequence-function maps ("variant-effect maps") that describe functional impacts of thousands of variants.[18]

[1]Donnelly Centre for Cellular and Biomolecular Research, University of Toronto, Toronto, ON M5S 3E1, Canada; [2]Department of Molecular Genetics, University of Toronto, Toronto, ON M5S 3K3, Canada; [3]Lunenfeld-Tanenbaum Research Institute, Sinai Health, Toronto, ON M5G 1X5, Canada; [4]Department of Computational and Systems Biology, University of Pittsburgh School of Medicine, Pittsburgh, PA 15260, USA; [5]Faculty of Health Science, McMaster University, Hamilton, ON L8S 4L8, Canada; [6]Maurice Wohl Clinical Neuroscience Institute, King's College London, London SE5 9RX, UK; [7]Labcorp Genetics (Formerly Invitae Corp.), San Francisco, CA 94103, USA; [8]Biogen Inc., Cambridge, MA 02142, USA; [9]Umeå University, 901 87 Umeå, Sweden
*Correspondence: wav14@pitt.edu (W.v.L.), fritz@pitt.edu (F.P.R.)
https://doi.org/10.1016/j.ajhg.2025.08.019.
© 2025 The Author(s). Published by Elsevier Inc. on behalf of American Society of Human Genetics.
This is an open access article under the CC BY license (http://creativecommons.org/licenses/by/4.0/).

These maps can accurately identify functional variants even for variants that had not yet been clinically observed before the map was produced. Moreover, functional evidence from variant-effect maps has already begun to assist variant reclassification.[18,19]

Here we employ highly multiplexed cell-based assays to proactively and systematically measure all missense variant impacts for the human SOD1 protein using multiplexed assays in both yeast and human cells. We find that the resulting impact scores of nearly all possible missense variants correspond well with prior knowledge about the SOD1 protein structure and with known patterns of mutational tolerance, and they also point to additional sequence-structure-function relationships. Finally, we demonstrate that variant-effect map scores provide new large-scale evidence to classify the pathogenicity of *SOD1* alleles.

Material and methods

SOD1 variant notation

Amino acid substitution notations (e.g., p.Ala5Val, p.Gly86Arg, p.Gly94Arg) correspond to the current SOD1 NCBI:NC_000021.9 code-determining nucleotide sequence.

Cell lines, yeast strains, and plasmids

The *Saccharomyces cerevisiae* strain (*MATa sod1Δ::KanMX his3Δ1 lys2Δ0 leu2Δ0 ura3Δ0*) used to assay activity for *SOD1* variant libraries was obtained from Horizon Discovery (formerly Open Biosystems). For yeast expression, wild-type (WT) and mutant SOD1 open reading frames (ORFs) were subcloned into the Gateway-compatible yeast expression vector pHYC-DEST2 (CEN/ARS based, ADH1 promoter, *LEU2* marker).[20] The *SOD1* ORF clone (Ensembl: ENSG00000142168; GenBank: NM_000454.5) was obtained from the Human ORFeome v.9.1 library.[21]

HEK293T T-REx Bxb1 landing pad cells, kindly provided by Dr. D. Fowler, were also used to assay *SOD1* variant libraries. To generate the Gateway-compatible human integration vector pDEST-HC-REC-v2 for the Bxb1 landing pad, the attB-PTEN-IRES-mCherry plasmid (also a gift from Dr. D. Fowler) was obtained and the Kozak sequence and *PTEN* ORF replaced with the 1873 bp Gateway attR1-CcdB-attR2 cassette from pDEST-AD-CYH2. We used overlap extension PCR to generate a "SOD1 abundance reporter" construct expressing GFP (GenBank: KC896843.1) fused to the C terminus of SOD1.

Using Gateway LR reactions, both WT and variant disease-associated *SOD1* ORFs were subcloned into the yeast expression vector (pHYC-DEST2), and both WT and variant versions of the construct encoding SOD1-GFP were subcloned into the pDEST-HC-REC-v2 human integration vector. ORF identity and expected mutations were confirmed by Sanger sequencing. Yeast expression vectors (including an empty-vector control bearing the ccdB marker under a bacterial promoter) were transformed into the appropriate *sod1Δ* yeast strain. Human integration vectors (including an "mCherry-only" negative control bearing a construct expressing mCherry in place of that expressing SOD1-GFP) were transfected into HEK293T cells.

Although the phenomenon of WT SOD1 stabilizing SOD1 variants through heterodimerization has been reported,[22] this has not been implicated as a pathomechanism for SOD1-ALS.[23] However, to allow for this possibility, we did not knock out the endogenous *SOD1* gene in the HEK293T cell line we used.

Validation of a yeast-based total enzymatic activity assay

From a single colony, *sod1Δ* yeast strain cells expressing human SOD1 WT, empty-vector control, or variant plasmid were grown to saturation at 30°C. Each culture was then adjusted to an optical density at 600 nm (OD_{600}) of 1.0 and serially diluted by factors of 5^1, 5^2, 5^3, 5^4, and 5^5. These cultures (5 mL of each) were then spotted on plates with synthetic complete (SC) medium with glucose as the carbon source, lacking leucine (SC-LEU), as appropriate to maintain the plasmid and incubated at either 30°C or 38°C for 48 h. After imaging, results were interpreted by comparing the growth difference between the yeast strains expressing human genes and the corresponding empty-vector control.

Small-scale abundance assay validation in mammalian cells

Analysis of GFP expression profiles in (mycoplasma-free) transfected HEK293T T-REx Bxb1 landing pad cells was conducted for WT, empty-vector control (mCherry+ only), and variants at day 7 post transfection with plasmid and Bxb1 recombinase following 72-h induction with 2 μg/mL doxycycline, using a Beckman-Coulter Gallios flow cytometer. Each population was gated for live (Sytox Red dead cell stain) and singlet cells (based on forward-scatter and side-scatter or FSC/SSC) and subsequently for integrants based on being negative for blue fluorescent protein (BFP) and positive for mCherry. Compensation was applied to correct for GFP fluorescence at the wavelength used for mCherry fluorescence detection.

Generating mutagenic libraries

Libraries were generated using an updated version of precision oligo-pool-based code alteration (POPCode) approach,[24] which eliminates a requirement of the previous POPCode approach[24] for a uracilated template. Oligos were designed along the entire coding region of SOD1, such that each oligo contained an "NNK" degenerate code centered on each codon (see Data S2 for primers). Although oligo designs differed only near the SOD1-GFP junction, for convenience we generated separate libraries for both SOD1 and SOD1-GFP. To generate the mutagenic libraries, the template plasmid backbone was denatured and the pooled, phosphorylated oligos were annealed along with primers that add an overhang "tag" to allow for preferential amplification of the mutagenized strand by PCR. A fill-in reaction was performed with Phusion MM (NEB), and Taq DNA ligase (NEB) was applied to seal the nicks. Subsequent PCR reactions amplified the mutagenized strand using the tag sequences and added attB1 and attB2 sites for Gateway cloning into destination vectors compatible for either yeast or human cell-based assays (see Data S2 for primers).

Multiplexed assay for total enzymatic activity

Total enzymatic activity of individual SOD1 variants was examined using four biological transformation replicates of the yeast-based functional complementation assay: the mutagenic library without GFP was transformed into the *S. cerevisiae sod1Δ* strain using the EZ Kit Yeast Transformation kit (Zymo Research),

and ~1 million transformants pooled to form the host library. Yeast transformants were grown at 30°C in SC-LEU medium (US-Biological) to ensure plasmid retention (pre-selection condition). Plasmid pools were prepared from 10 optical density units (ODU) of cells (defined as the number of yeast cells in 10 mL of a 1 OD_{600} culture, typically 10^8 cells) and used as templates for downstream tiling PCR. Two biological replicates of approximately 4×10^8 cells from each of the two independent transformant pools were each inoculated into 200 mL of SC-LEU medium and grown at restrictive temperature (post-selection condition; 38°C) for 48 h (four total biological replicates). In parallel, the *sod1Δ* yeast strain was transformed with the WT ORF and grown alongside the mutagenic pool. Plasmids were extracted from 10 ODUs of cells from each culture (two replicate cultures for both post-selection condition and WT control) and used as templates for downstream tiling PCR (see Data S2 for primers).

Large-scale mammalian cell-based assays

The impact of *SOD1* variation on protein abundance was evaluated in a human cell line using the variant abundance by massively parallel sequencing (VAMP-seq) method.[25] The mutagenized library expressing SOD1-GFP library and Bxb1 recombinase in a 10:1 stoichiometric ratio was transfected using FuGene 6 into two biological replicates of approximately 15×10^6 HEK293T Bxb1 landing pad cells, enabling the expression of a single variant per cell. After induction with 2 μg/mL doxycycline, ~2 million cells per replicate were sorted 7 days post transfection using a SONYMA900 cell sorter, gated for live cells (those unstained with SYTOX Red dead cell stain), singlets based on FSC/SSC, and enriched for integrated cells lacking BFP and having mCherry expression. Integrated cells were expanded for 7 days and ~2 million cells sorted on the SONYMA900 for the post-selection condition: high GFP and high mCherry (corresponding to high transcription and high abundance of SOD1-ALS). The pre-selection condition was 2 million cells re-sorted for integration with the mCherry marker on the same day as the post-selection condition. Cells of the pre-selection and post-selection conditions were expanded for 7 days and pellets of 1×10^7 cells were collected. gDNA from two pellets for each biological replicate condition was extracted with the Sigma gDNA extraction kit. Purified DNA was amplified using primers BxbAmp_Tet_F2 and Bxb1Amp_R1 (see Data S2 for primers) and used as templates for downstream tiling PCR (see below). Each biological transfection replicate had three technical tiling PCR replicates.

Sequencing and large-scale analysis

For plasmid (total enzymatic activity) and amplicon (abundance) libraries from both pre-selection and post-selection conditions, five short template amplicons (~150 bp) that tile the *SOD1* ORF were amplified with primers carrying a binding site for Illumina sequencing adaptors (see Data S2). In a second-round PCR, Illumina sequencing adaptors with index tags were added to the first-step tiling amplicons. Paired-end sequencing was conducted on all tiles, significantly reducing base-calling errors and enabling accurate detection of very low (parts per million) variant frequencies. Each assay was independently sequenced using an Illumina NextSeq 500 with a NextSeq 500/550 High Output Kit v.2, achieving a sequencing depth of >1.3 million paired-end reads per tile. Sequencing reads were demultiplexed with bcl2fastq v.2.17 (Illumina).

Raw read data were processed and functional impact (total enzymatic activity or abundance) scores for variants were assigned as previously described.[24,26] Briefly, we quantified variant allele frequencies using TileSeq_MutCount (https://github.com/RyogaLi/tileseq_mutcount), which incorporates Bowtie2 for aligning paired sequencing reads to the reference template. The posterior probability for each divergent base call was determined, and those surpassing the 0.9 threshold were counted. We then filtered out variants with read counts less than 10 or frequencies below the 90th percentile of the WT. Next, we generated a score for each variant using the tileseqMave (https://github.com/jweile/tileseqMave) pipeline. After calculating the error-corrected enrichment ratios by subtracting WT variant frequencies from both the pre-selection and post-selection frequencies, a functional impact score was determined for each variant based on its relative enrichment compared to the median ratios of nonsense and synonymous variants. Scores were then rescaled such that the medians of nonsense and synonymous variants were 0 and 1, respectively. Biological replicate scores were averaged, and error in the average was estimated using the delta method of error propagation. Use of inverse-variance weighted averages provided greater weights for scores with lower uncertainty. For variants with no replicate, the standard error and degrees of freedom were taken directly from the existing biological replicate.

Reference sets and benchmarking

To initially evaluate the ability of total enzymatic activity and abundance assays to predict variant pathogenicity, we curated a small reference set of pathogenic/likely pathogenic (P/LP) variants from ClinVar selecting those that spanned the length of the *SOD1* coding sequence. "Proxy-benign" (PB) p.Asn20Ser and p.Gly130Ser variants were chosen based on their presence in gnomAD with no corresponding annotation.

To assess the ability of variant-effect maps to identify pathogenic variants in large scale, we then used a positive set of 84 and 41 variants annotated as P/LP based on Labcorp Genetics's variant interpretation framework (Sherloc)[27] and ClinVar P/LP variants with multiple submitters (ClinVar accession date: December 29, 2024),[12] respectively. Due to the absence of reported benign variants, we augmented the negative set with 100 PB variants from gnomAD v.4.1.0 (gnomAD accession date: December 21, 2024), requiring only that these variants have no reported clinical annotations. The final reference sets were called "ClinVar/gnomAD" and "Labcorp/gnomAD." The Labcorp/gnomAD reference set was supplemented with one benign variant (B) sourced from the Labcorp dataset. To assess the performance of the maps and computational predictors of disease variants, we evaluated the trade-off between precision (proportion of variants below a threshold that are pathogenic) and recall (fraction of known pathogenic variants identified).[28] To account for reference set size imbalances and varying prior probabilities, we generated "balanced" precision-recall curves, which weigh the positive and negative reference sets 50/50 using a Bayes' rule-based method adjusting precision for prior probability.[29]

To determine whether variants were tolerant or intolerant to variation in either the activity or abundance map, we initially set a score threshold of <0.5 as deleterious, corresponding to the midpoint between the median scores of nonsense (0) and synonymous (1) variants. See Data S2 for variant "quadrant" classifications based on tolerance thresholds defined in Figure 2.

Transformation of scores to log likelihood ratios of pathogenicity

To provide a quantitative Bayesian evidence weight for clinical variant interpretation, we estimated the log likelihood ratio of pathogenicity (LLRp) for each variant's abundance score. To this end, probability density functions were separately estimated for positive and negative reference variant score sets using kernel density estimation (more specifically, the Epanechnikov kernel[30] with bandwidth determined by the Sheather and Jones method[31]). For variant scores in each map, a pathogenic:benign log ratio was calculated using the estimated probability density functions. LLRp values were then calibrated against thresholds established by the ACMG/AMP variant classification system using an adapted version[26] of the calibration approach developed by Tavtigian et al.[32] See Data S2 for variant LLRp values and for evidence strengths provided by the abundance map only (given poor precision vs. recall performance and lack of evidence strengths provided by the activity map).

Thermostability calculations

Toward determining whether variant impacts in our total enzymatic activity map could be attributed to changes in steady-state protein level (abundance), as opposed to specific activity, we calculated protein thermostability ($\Delta\Delta G$) values. Calculations of $\Delta\Delta G$ were obtained from the stability predictor DDGun3D version 0.0.2.[33] The PDB entry 1HL5[34] for SOD1 was used to predict these values, and it satisfied the following conditions: an X-ray-determined structure with resolution 1.8 Å or better and no missing or non-standard residues. We defined stabilizing amino acid substitutions as those for which $\Delta\Delta G > -0.1$ and a destabilizing substitution for $\Delta\Delta G < -0.5$. Scores obtained from DDGun3D are available in Data S2.

Population allele frequencies and SOD1-ALS cohort data

SOD1 variant allele frequencies were retrieved from population databases, including gnomAD v4.1.0 (https://gnomad.broadinstitute.org/) and the UK Biobank (application ID: 51135). Odds ratios of damaging or neutral variant depletions for both maps were calculated using non-overlapping sets of variant allele frequencies from the population databases.

SOD1-ALS cohort data, including age of onset; disease duration; and SOD1 enzymatic specific activity, abundance, and half-life (measured in mice), were obtained from Huang et al.,[35] which aggregated information from the literature. In the case where multiple papers reported data for a given variant (e.g., enzymatic activity of SOD1 p.Leu118Val was measured as both 61%[36] and 88%[37]), a weighted average was calculated with weights proportional to the sample size in each study.

Structure analyses

We used PyMOL to place map scores in the context of solved crystal structures of apo-SOD1 (PDB: 1HL4),[34] holo-SOD1 (PDB: 1HL5),[34] and the Zn^{2+}-replete SOD1-copper chaperone for SOD1 (CCS) complex (PDB: 6FP6).[38] To simulate the binding of the de-metalated intermediate (monomer) of SOD1 (PDB: 1RK7)[39] with CCS (PDB: 1DO5),[40] we used the ClusPro web server (https://cluspro.bu.edu/publications.php) with default parameter settings for protein-protein docking.[41–45] The monomeric SOD1 and CCS PDB structures were uploaded to the server as the ligand and receptor, respectively. ClusPro initially performs rigid-body docking, clusters the top 1,000 complexes based on root-mean-square deviation (RMSD), then refines and ranks these complexes through energy minimization using the CHARMM force field.

Residues at the interface (within a threshold of 5.0 Å) of homo- and heterodimer complexes were identified using the PRODIGY-crystal (https://wenmr.science.uu.nl/prodigy) web server, which bases its predictions on structural and energetic features.[46,47] We used the FreeSASA program (https://freesasa.github.io/) to calculate the relative solvent exposure of residue positions.[48] After examining the distribution of relative solvent exposure values, we established thresholds corresponding to the high and low peaks. Residues with surface area values exceeding 35% were considered exposed, while those below 20% were classified as core ("buried").

Setup of molecular dynamics simulations

To examine variant-specific impacts on the dynamic behavior of SOD1, especially movements associated with metal binding, we performed molecular dynamics (MD) simulations using a high-resolution monomeric SOD1 structure (PDB: 2C9V,[49] chain A). The apoenzyme system was constructed by removing metal ions (Zn^{2+} and Cu^{2+}) and introducing site-specific mutations with PyMol (version 1.3, Schrödinger). MD simulations were conducted with the NAMD software suite, employing the CHARMM36m force field, and the system was prepared via the CHARMM webserver (https://www.charmm-gui.org/),[50] reflecting physiological conditions (pH 7.4). The N terminus and C terminus were patched to their charged states, and a disulfide bond was established between Cys58 and Cys147.

The protein was solvated in a rectangular box of TIP3P water molecules with a 10.0-Å minimum distance from the edge of the box. To simulate physiological ionic strength, 0.15 M potassium chloride was added using the Monte Carlo method.

Production simulations were run at 310 K for 400 ns across five independent runs. MD trajectories were analyzed using the Python package ProDy. System stability was assessed by calculating the mean-square fluctuations (MSFs) of backbone atoms across the five runs. Secondary structure and flexibility profiles were examined, and hydrogen-bond interactions were defined with a 120° angle (donor-hydrogen···acceptor) threshold and a 3.4-Å distance threshold between the donor and acceptor heavy atoms.

Results

Implementing scalable functional assays for SOD1 missense variants

For *SOD1*, previous small-scale cell-based assays have been based on the premise that pathogenesis is via toxic gain-of-function variants.[51–53] More specifically, the model for SOD1-ALS suggests that gain-of-function SOD1 variants cause SOD1 polymerization and consequent toxicity.[54] Although cell-based aggregation assays have been used as evidence in support of clinical variant annotation, these assays have either used cells from individuals (which is not scalable) or required either transient transfection[53] or lentiviral integration,[51] yielding SOD1 variant expression that potentially goes well beyond physiological levels. We therefore sought to identify a scalable

cell-based assay for the propensity of SOD1 variants to aggregate that employs moderate SOD1 expression levels. Unfortunately, despite pursuing both yeast- and human cell-based assays under a variety of sensitizing conditions, we were unable to develop such an assay (see Supplemental Note in Document S1).

Many ALS-associated pathogenic SOD1 variants exhibit reduced function, and variants that completely ablate SOD1 enzymatic activity have been linked to a progressive, adult-onset ALS phenotype,[55] an infantile SOD1-deficiency syndrome (iSODDES; ALS phenotype),[56] and a neurological disorder distinct from ALS.[57–59] Pathogenic SOD1 variants observed in people with ALS have been described as, in addition to having gain-of-function (aggregation) effects, also being highly variable in enzymatic activity. Understanding the mechanism of ALS-associated SOD1 variants is made complicated by the fact that they can display decreased half-lives and either higher- or lower-than-normal protein expression levels.[6,57,60–62] Moreover, both expression and enzymatic activity of SOD1 variants can be tissue dependent.[63] We therefore next sought to assess the impact of SOD1 variants on both enzymatic function and protein abundance.

First, to investigate the functional impact of missense variation in SOD1 at scale, we implemented a previously validated humanized yeast model. Yeast *sod1Δ* strains are known to have growth defects under heat-stress (38°C) growth conditions, and we confirmed that expression of human *SOD1* cDNA rescues this growth defect.[24,64] We evaluated this assay by measuring growth rescue for a small set of high-confidence pathogenic variants as defined by multiple ClinVar submitters (Figure S1). This set of variants included some known to exhibit both aggregation and loss-of-enzymatic-activity effects (p.His47Arg and p.Asn132Lys), some known to exhibit aggregation for which the impact on activity had not been previously reported (p.Gly94Arg, p.Ile114Thr, and p.Leu145Phe), and also included both p.Asn20Ser and p.Gly130Ser as PB controls drawn from gnomAD (as no benign variants had been reported in ClinVar). Each variant, together with a positive-control WT human *SOD1* and an empty-vector control, was expressed in the yeast *sod1Δ* strain under thermal stress. We observed complementation for all non-pathogenic variants (100% precision) while observing lack of complementation for two of five pathogenic variants (40% recall). Although, ideally, pathogenic variants known to have gain-of-function effects (e.g., p.His47Arg, p.Gly94Arg, and p.Ile114Thr) would have exhibited reduced growth at both temperatures, no SOD1 variants showed toxicity under normal-temperature growth conditions. However, these preliminary results still supported larger-scale application of the assay, in that a variant-effect map achieving this level of performance could still be considered clinically useful.

Next, to assess the impact of SOD1 variants on protein abundance, we implemented a VAMP-seq assay, in which the protein of interest is fused to GFP. Using this approach, variants causing misfolding or destabilization followed by degradation can be detected on the basis of reduced GFP expression.[25] Constructs containing *SOD1-GFP* variants were introduced into human HEK293T cells and integrated at a landing pad containing a *Bxb1* recombination site flanked by an inducible doxycycline-inducible promoter on one side of the *Bxb1* site, enabling expression of a BFP gene on the other side. As a result, cells with integration events (which can be enriched by sorting for cells lacking BFP) each express a single variant SOD1-GFP.[25] Because SOD1-ALS generally presents in people who are heterozygous for a dominant mutation, no endogenous copy of *SOD1* was knocked out.

We initially subjected a small set of high-confidence (multiple submitters on ClinVar) reported pathogenic gain-of-function (aggregating) SOD1 variants to this assay, measuring GFP abundance in each integrated cell line via flow cytometry (Figure S2). This set of variants included some known to exhibit both aggregation and loss-of-enzymatic-activity effects (p.Ala5Val, p.His47Ala, p.Gly86Arg, p.Gly94Arg, p.Arg116Gly, and p.Leu145Phe[35,36,64]). We included p.Gly130Ser as a PB control drawn from gnomAD. All variants we tested showed distributions of GFP intensity that were lower than that of WT SOD1, although the pathogenic p.His47Arg and p.Leu145Phe variants and p.Gly130Ser PB control showed intermediate GFP levels (higher than all pathogenic variants tested, but lower than WT).

Systematically testing SOD1 variant effects on enzymatic activity and protein abundance

To measure functional impacts for all possible missense SOD1 variants, we next adapted the above-described total enzymatic activity and abundance assays to be multiplexed assays of variant effects, using the previously described TileSeq framework (Figure 1A).[24] As an initial step, we constructed mutagenized libraries of SOD1 variants (with and without C-terminally fused GFP) by using a large-scale oligo-directed codon mutagenesis protocol (see material and methods). Each mutagenized library was initially generated as a pool of amplicons and transferred en masse via two steps of recombinational subcloning (see material and methods) into one of two alternative destination vectors. Care was taken in this process to maintain pool complexity such that the average variant appears in at least 50 independent clones. Mutagenized destination-vector libraries were introduced into the appropriate yeast or human assay cells and subjected to the appropriate selection procedure:

First, to assess the loss-of-function effects of SOD1 variants in parallel, the mutagenized library without fused GFP was cloned en masse into yeast expression vectors (designed to express SOD1 using the moderate-strength yeast *ADH1* promoter) and transformed into the yeast *sod1Δ* strain. In order to maintain the expression plasmid, the transformed yeast strain pool expressing human SOD1

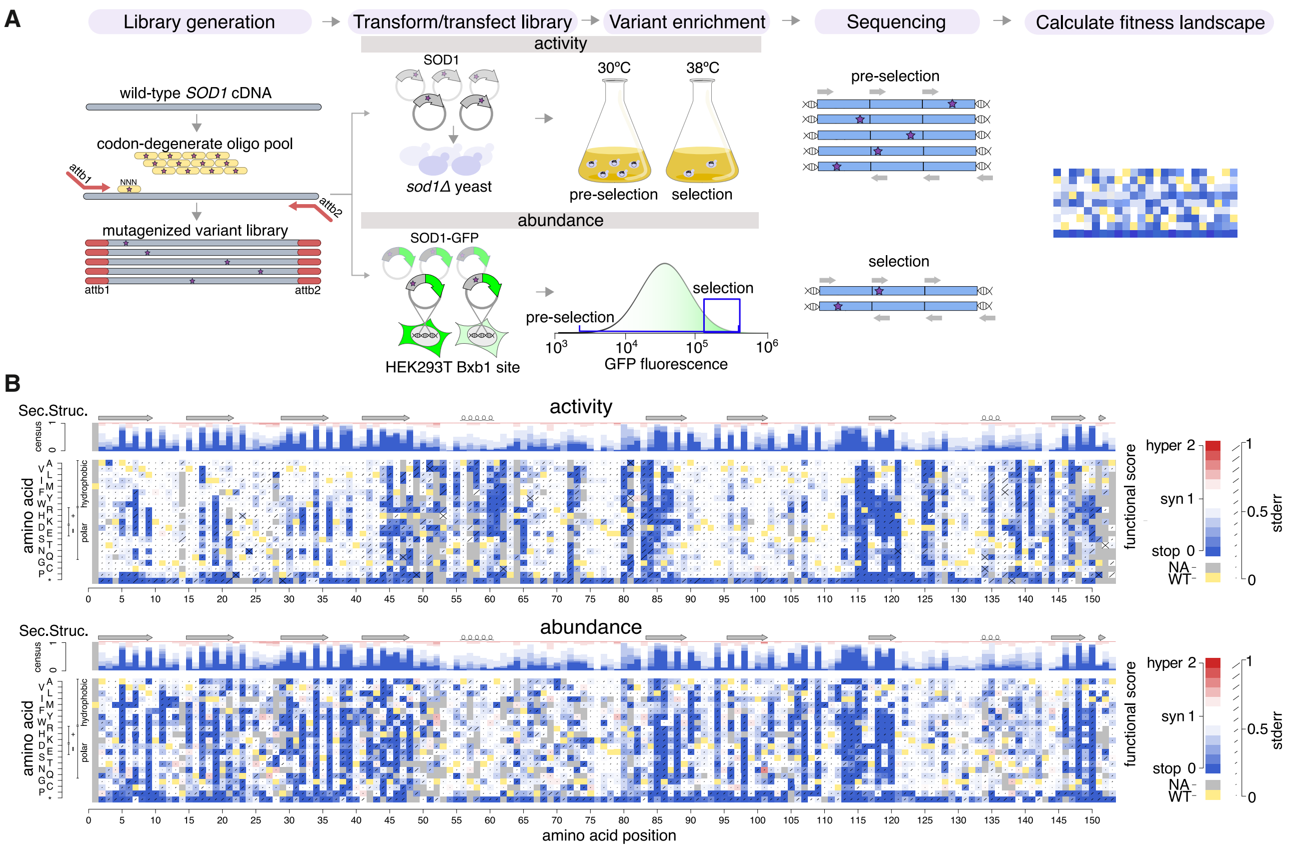

Figure 1. Generation of SOD1 missense variant-effect maps
(A) Overview of process to generate variant-effect maps.
(B) SOD1 variant-effect maps measuring total enzymatic activity (top) and abundance (bottom). Box color indicates the WT residue (yellow); a substitution with damaging (blue), tolerated (white), or above-WT ("hyper"; red) functional score; or missing data (gray). Consensus tracks summarize the map scores by position. β strands are indicated by arrows, and α helices by loops.

variants was maintained in Leu-synthetic medium. To enrich for SOD1 variants that retain enzymatic function, cells were grown competitively at the restrictive (38°C) temperature at which SOD1 activity is essential. The relative frequencies of variants were measured in cell pools in both pre-selection and post-selection conditions.

Second, to assess SOD1 variant impacts on protein abundance, we used the VAMP-seq method in cultured human cells to measure the steady-state abundance of human SOD1 variants. The mutagenized library was designed such that each *SOD1* variant clone also encoded a C-terminal GFP fusion followed by an internal ribosomal entry site (IRES) and a coding region for mCherry (as a marker of both transfection and construct expression level). This library was recombined into HEK293T cells containing the *Bxb1* landing pad, simultaneously disrupting BFP expression at the landing pad and enabling expression of SOD1-GFP and mCherry via the TetOn promoter upstream of the Bxb1 site. Stable recombinant integrant cells harboring SOD1 variants (BFP−, mCherry+ cells) were then isolated by fluorescence-activated cell sorting (FACS) and expanded. To enrich for SOD1 variants that retain protein abundance, we selected for a population with both high GFP and mCherry expression (post-selection condition) and measured the relative frequencies of variants in these cells relative to all SOD1 integrant cells (the pre-selection condition).

For both total enzymatic activity and abundance assays, we obtained functional scores of each SOD1 variant by measuring the relative abundance of that variant in the post-selection relative to the pre-selection condition, using the previously described TileSeq approach.[24] Briefly, five ~150-bp tiles, collectively covering the entire gene coding region, were subjected to "duplex sequencing" (in which each single-molecule-derived "colony" of PCR products is sequenced on both strands), allowing lower base-calling error. Each nucleotide position had a sequencing depth of at least 1.3 million reads, detecting 96.5% of all possible missense variants and 99% of those amino acid substitutions that are achievable via a single-nucleotide variants (SNVs) (Figure S4A). We considered variants seen at a frequency of at least 10 counts per million reads in the pre-selection condition to have been well measured. This criterion was met by 2,655 (87% of all possible) and 2,632 (86% of all possible) missense variants in each map and 85% of all possible SNV-accessible substitutions detected for both the activity and abundance maps (Figure S4A). The average number of SOD1

codon substitutions per cell was estimated at 0.64 for activity and 0.83 for the abundance map (Figure S4B). Pre-select variant frequencies in biological replicates were highly correlated for both activity and abundance maps (Figure S4C; Pearson's R activity = 0.99; R abundance = 0.94). The uncertainty (standard error) for each functional score was also estimated as previously described,[26,28] based on trends in the extent of agreement between biological replicates as a function of variant frequency in the pre-selection condition. After the filtering described above, the scores for all variants were rescaled such that 0 corresponds to the median of nonsense variants and 1 to the median of synonymous variants.

Biological replicate scores showed high agreement for both the total enzymatic activity (Pearson's R = 0.81) and abundance (Pearson's R = 0.79) maps (Figure S4D). To generate the final scores, we averaged the biological replicates for each variant and estimated error in the final score as described in material and methods. Figures S4E and S4F provide distributions of standard error and standard deviation for the activity and abundance variant scores. We observed nonsense variants to have substantially lower scores than synonymous variants (Figure S4G). A bimodal distribution of missense variants was observed for both maps, with modes that fell close to those of nonsense and synonymous variants, presumably corresponding to either deleterious or neutral effects, respectively (Figure S4G). The peaks corresponding to deleterious variants (score < 0.5; see material and methods) in the activity and abundance maps contained ∼25% and ∼33% of missense variants, respectively. Figure 1B visualizes the complete variant-effect maps for both total enzymatic-activity and protein-abundance assays.

Comparing variant effects between activity and abundance maps

We next evaluated, for each map, the deleteriousness of substitutions to each of the 19 possible non-WT amino acids. To this end we calculated, for each substitution, the median score for all instances of substitution of the reference residue with that amino acid. For both maps, the correlation of this "deleteriousness vector" with the corresponding vector derived previously from 28 other variant-effect maps[65] was strong (Figures S5A and S5B; Spearman's R activity = 0.86, $p < 2e{-}16$, R abundance = 0.90, $p < 2e{-}6$), giving further evidence that the biochemical impact of changes to amino acids is captured. We also evaluated the impact of substitutions of the initial amino acids. The correlation of the aggregated "from" amino acid scores to each the 28 other variant-effect maps[65] was moderate for the activity map and not significant for the abundance map (Figures S5C and S5D; Spearman's R activity = 0.48, $p = 0.045$, abundance $p > 0.05$). We note that there is reduced power in the "from" amino acid analysis given that many amino acids do not appear often in the reference sequence.

If the impact of missense variation on total enzyme activity was predominantly due to changes in protein abundance (e.g., arising through reduction of stability), we would expect high correlation between our total enzymatic activity and abundance maps. To assess this, we initially aggregated scores for each of the possible "to" and "from" amino acids. We observed high correlation between abundance and activity maps in terms of the deleteriousness trends for "to" amino acids and moderate correlation in terms of the "from" amino acids (Figures S5E and S5F; Spearman's R to amino acid = 0.80, $p < 4e{-}5$, Spearman's R from initial amino acid = 0.56, $p = 0.02$). Although measuring correlation between abundance and activity maps using individual substitution scores yielded weaker correlation, it remained statistically significant (Figure S5G; Spearman's R = 0.25, $p < 2e{-}16$). The incomplete correlation we observed also supports the idea that many SOD1 variants are deleterious without impacting protein levels. However, for those variants exhibiting low abundance scores, we see a corresponding increase in the proportion of low activity scores (36%) relative to variants with high abundance scores (20%) (Figure S5H).

To characterize differences or similarities between activity and abundance maps, we divided the correlation between abundance and activity missense variant scores into four distinct quadrants, each defined by a specific color (Figure 2A): (1) deleterious in both maps (lower-left quadrant, blue, 12% of missense variants); (2) neutral in both maps (upper right, white, 54%); (3) deleterious in only the activity map (lower right, purple, 13%); and (4) deleterious in only the abundance map (upper left, teal, 21%). Quadrants were defined by threshold scores of 0.5 in each map (see material and methods). We next visualized both the SOD1 structure by coloring each residue position according to the predominant quadrant (if any) at that position (Figure 2B) and map on the amino acid substitution level (Figure 2C). Interestingly, variants that were identified as damaging in the activity map and neutral in the abundance map (purple quadrant) clustered around metal binding or CCS-interacting positions: Indeed, 18 of 28 (64%) positions in this quadrant involved metal binding, the electrostatic loop, homodimerization, or CCS binding (Data S2), illustrating the importance of residues at these positions for the enzymatic activity of SOD1. Positions that were found deleterious in both maps (blue quadrant), as well as positions found deleterious in the abundance map and neutral in the activity map (teal), tended to be at the buried residues in β strands, with the blue positions tending to be at the C-terminal edges of β strands (discussed further below).

Initial evaluation of map quality

As an initial evaluation of the quality of our maps, we compared with the small-scale assays that had been used to validate the assays. For example, the P/LP variants p. Arg116Gly and p.Ala146Thr, which our small-scale

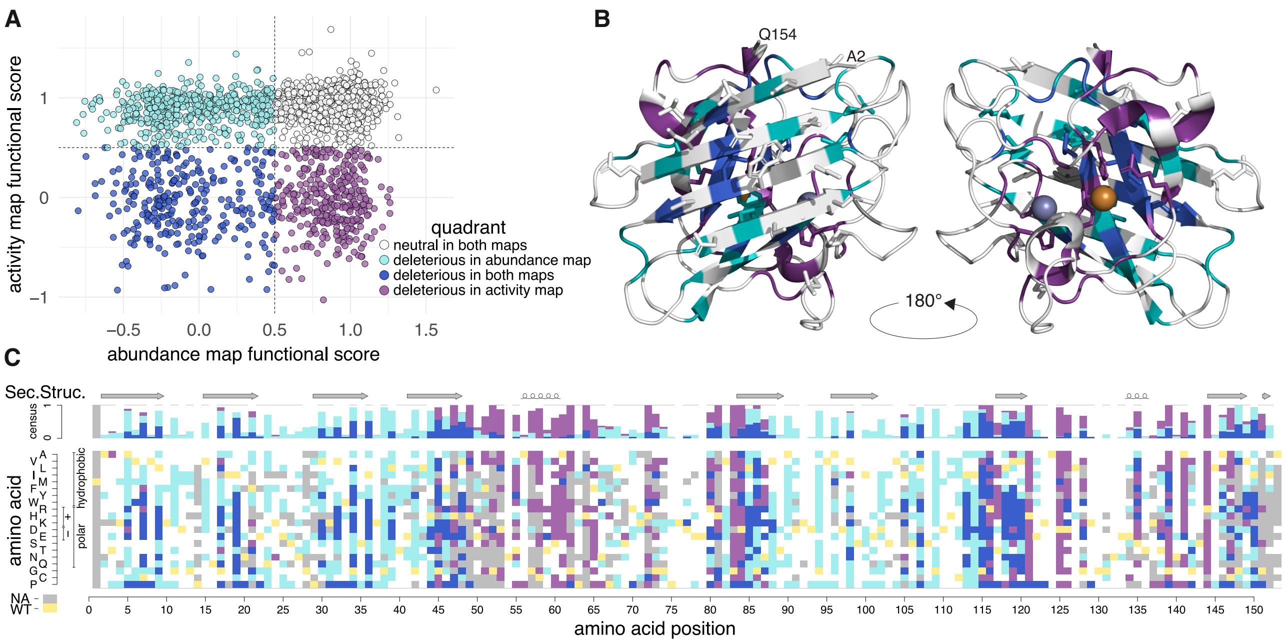

Figure 2. Comparison between activity and abundance scores

(A) Scatterplot representing the combination of activity and abundance score for each variant, divided into quadrants (defined by scoring either above or below a score of 0.5, the midpoint between nonsense and synonymous scores) as follows: neutral in both maps (white), only deleterious in the abundance map (teal), deleterious in both maps (blue), and only deleterious in the activity map (purple).

(B) Structure of SOD1 (PDB:1HL5),[34] with Cu^{2+} (orange)/Zn^{2+} (blue-gray) ions, in which each residue is "painted" according to the dominant quadrant for substitutions at that position. Positions Val32 and Arg80, which did not show a dominant quadrant type, were painted gray.

(C) Map of missense variants painted by quadrant type for variants measured in both maps. Secondary structure is indicated by arrows for β strands and loops for α helices. The WT residue is shown in yellow, while gray indicates that the substitution was not well measured in at least one of the maps.

abundance assay found damaging (these were not tested in our small-scale activity assay), appeared damaging in our abundance map. Finally, the PB control p.Gly130Ser present in gnomAD, which appeared tolerated in small-scale assays, was also tolerated in both maps. Except for p.Asn132Lys, which was found damaging in small-scale activity assays and not in our large-scale activity map, there were no clear examples of discordance between large- and small-scale assay results.

As a first evaluation of the relevance of our maps to human biology, we investigated whether variants that appeared deleterious in our maps are selected against in the population. A previously described missense constraint *Z* score[66] derived from analysis of *SOD1* variants in gnomAD was 1.2, indicating moderate selective pressure against these variants. In keeping with this, missense variants classified as damaging in our activity and abundance maps were less common in human cohort databases (see material and methods; total activity log-odds ratio = −1.08 and abundance log-odds ratio = −1.74; Fisher's exact test; Figure S6). Thus, variants found damaging in each of our maps appear to be counter-selected in the human population.

Interestingly, a loss-of-function intolerance (pLI) score[66] applied to gnomAD yielded a score 0.01 for *SOD1*, suggesting general tolerance to nonsense and frameshift variants. This is consistent with the view that deleterious SOD1 variation is primarily due to its dominant gain-of-function impacts.

We next investigated, for both maps, trends related to amino acid substitutions with different properties. As expected, substitutions at hydrophobic residue positions had significantly more damaging effects in the activity map compared to substituting polar and charged amino acids (Figure S7A; adjusted p (p_{adj}) = 2e−10 and 1e−10, respectively, Wilcoxon test). Results for the abundance map were similar (Figure S7B; p_{adj} = 7e−30, 2e−37, respectively, Wilcoxon test). As expected, conservative substitutions at uncharged positions tended to be tolerated (higher scoring) in both maps relative to substitutions of other types (Figure S7C). Additionally, we found that substitutions to proline tended to be damaging in both maps (Figure S7D; activity p_{adj} = 2e−9, abundance p_{adj} = 1e−7), as expected given the general tendency of proline residues to disrupt secondary structure.

Biochemical insights from SOD1 variant-effect maps for activity and abundance

Substitutions that reduce both the net negative charge of SOD1 and also stability have been associated with lower survival time in ALS.[67] In keeping with this, the activity map showed that introduction of negatively charged

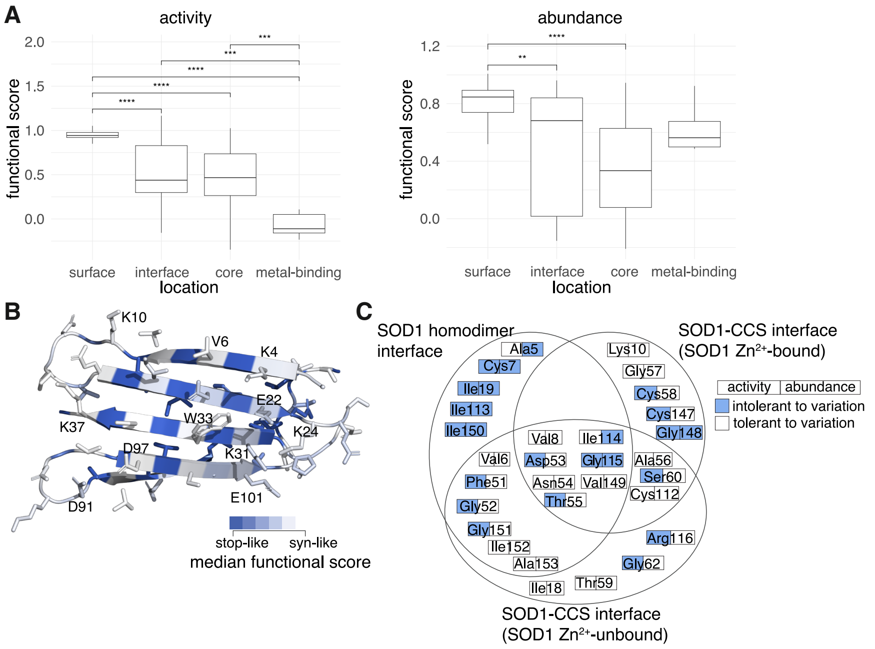

Figure 3. Modeling the effects of SOD1 missense variants on protein structure

(A) Distributions of median functional scores (where each median is of missense substitutions at each residue position) are shown for total enzymatic activity (left) and abundance (right) maps, summarizing sets of positions where the WT residue is "buried" (below 20 Å^2 accessible surface area [ASA]), "metal-binding," "surface" (above 35 Å^2 of ASA), and "interface" (at the homodimerization interface). Boxes correspond to interquartile range, and bold bars indicate medians of the positional medians. Whiskers correspond to minima and maxima. p values were calculated by Mann-Whitney U test, ****$p < 0.0001$, **$p < 0.001$.

(B) Structural model of SOD1, colored according to the median functional score of substitutions at each position, with positions tolerant to substitutions in white and positions intolerant to substitution in blue.

(C) Venn diagram indicating whether amino acids found at SOD1 interfaces (either the homodimeric SOD1 interface or the SOD1-CCS interface with and without Zn^{2+} bound to SOD1) show for each map whether the variant was tolerant (white) or intolerant (blue) to substitutions.

See material and methods for details on determination of threshold scores.

amino acids tended to have a greater impact (lower score) than introduction of other amino acids (Figure S7D; Δmedian 0.08, $p_{adj} = 0.03$), an effect that was even more strongly observed in the abundance map (Figure S7D; Δmedian 0.14, $p_{adj} = 9e{-4}$). This fits the previously proposed model that unstable SOD1 variants with maintained or modestly lowered net negative charge will tend to be more soluble and therefore more likely to form toxic oligomers rather than accumulating as more benign larger aggregates.[68]

We assessed whether our scores agreed with known secondary structural features of SOD1 given the importance of core (buried) residues to protein stability (Figure S8; ASA < 20 Å^2). As expected, substitutions to buried residues were poorly tolerated in both maps relative to non-homodimer surface residues (Figure 3A; Δmedian score between buried and non-homodimer surface residues = 0.48, 0.51, $p_{adj} = 6e{-12}, 4e{-9}$, activity and abundance, respectively). Interestingly, the lowest-scoring categories of residue positions were metal-binding positions for the activity map and buried positions for the abundance map. This supports the expected importance of metal binding for enzymatic activity of SOD1, while protein abundance depends strongly on the buried positions that would be expected to be important for maintaining stability.

Hypothesizing that variants within the β sheets of SOD1 would be enriched for stability impacts, we "painted" a crystal structure of the active SOD1 homodimer[34] according to the median score at each position for both maps (Figures S9A and S9B). Interestingly, residues intolerant to variation formed distinct "stripes" on the β sheets such that the intolerant positions tended to correspond to hydrophobic residues with inward-facing side chains in both maps.

To gain further insight into the mechanism of variant impacts, we examined activity and abundance map scores at specific residues of known biochemical importance in the context of the sequence of events known to promote SOD1 stability and activity. Briefly, this sequence begins with Zn^{2+} binding, Cu^{2+} binding, then associated release of CCS, formation of a disulfide bond between Cys58 and Cys147,[69–72] followed by formation of the active SOD1 homodimer.

We found that positions coordinating the binding of Zn^{2+} (His72, His81, Asp84), Cu^{2+} (His47, His49), or both metals (His64) were generally intolerant to mutations, especially in the activity map (Figures S10A and 2C), suggesting we have captured the importance of metal binding to enzymatic activity. Substitution of the disulfide-bonded Cys58 and Cys147 residues also showed severe functional defects in the activity map. Interestingly, we found substitutions at the His121 residue (which has been implicated in Cu^{2+} binding[73]) impacted total activity but not abundance, suggesting a critical role in enzyme function that is independent of protein abundance. We expected that residues at the homodimer interface or within the core would be more sensitive to variation than non-interface surface residues (accessible surface area [ASA] >35 Å^2). Indeed, for the activity and abundance maps, we found that substitutions at the homodimer interface exhibited more severe functional defects than other surface residues (Figure 3A; Δmedian = 0.50; p_{adj} = 1e−6, Δmedian = 0.16; p_{adj} = 5e−3 for activity and abundance map, respectively, by Mann-Whitney U test).

We next considered the electrostatic loop VII (residues 121–142), which contributes to a positively charged environment through which negatively charged superoxide ions diffuse to the active site. Residues Lys137 and Thr138 within this loop, as well as the adjacent Arg144 residue, have been previously highlighted as important.[74] Our abundance map supported some contribution for Thr138 to stability but apparently not causing enough loss of total activity to affect fitness in our activity assay (Figure S10A). Residue Lys137 was tolerant to substitution in both maps. Interestingly, Arg144 was intolerant to substitution in the activity map, but not the abundance map, suggesting a stability-independent role in enzymatic function for this residue also.

Modifications, such as the oxidation of WT residues—specifically the solvent-exposed residues Val6, Trp33, and Cys112—have been linked to variant aggregation and to the propagation of gain-of-function effects.[75–77] Surprisingly, Val6 and Trp33 were both highly tolerant to substitution in both activity and abundance maps. Hydrogen bonding by Cys112 to both Glu50 and Arg116 residues has been noted as important to maintaining SOD1 structural integrity.[78] Also surprisingly, both maps indicated that, except for hydrophobic substitutions in the abundance map, Cys112 was quite tolerant to substitution (Figure S10B). Excepting hydrophobic substitutions, we found variation at Glu50 to also be generally tolerated, while both Arg116 and the Arg116-neighboring residue Gly115 (which may convey flexibility at Arg116) were intolerant to mutation.

It has previously been hypothesized for the aggregation-prone β-amyloid protein that specific key apolar residues can prevent aggregation by attenuating local hydrophobicity (the "amyloid-stretch" hypothesis).[79] Our analysis revealed that the charged Glu22 and Lys31 residues are somewhat intolerant to change in the abundance map (Figure S10C), which would be consistent with the amyloid-stretch hypothesis if loss of attenuating effects of these residues on neighboring hydrophobic residues Trp33 and Val6 resulted in aggregation.

There are two previous "β-edge" hypotheses related to the impact of charged residues at the edges of a β sheet. First, it has been hypothesized (for β sheet-containing proteins in general) that lysines located on edges of otherwise-aggregation-prone β sheets are important for protein solubility.[80] Second, negatively charged residues on β sheet edges have been described as gatekeepers preventing aggregation.[81] In contrast to the lysine β-edge hypothesis, and despite the fact that many of SOD1's lysine residues are located on the edges of the N-terminal β sheet (more specifically Lys4, Lys10, Lys24, Lys31, and Lys37), all of these lysines appeared tolerated in our activity map, and all but Lys31 were tolerated in the abundance map (Figures 3B and S10C). Our data are more consistent with the negatively charged β-edge hypothesis, in that most of the negatively charged residues on the β sheet edge (Glu22, Asp91, and Glu101, but not Asp97) appear intolerant to variation in our abundance map. Overall, however, the observation that all charged positions at the edge of a β sheet appeared highly tolerant to variation in the total enzymatic activity map keeps us from drawing black-and-white conclusions about the consistency of our results with either of the β-edge hypotheses.

In addition to analyzing our scores in the context of the active SOD1 homodimer structure, we also considered SOD1 complexed with its paralog, the CCS.[38] We examined both a reported Zn^{2+}-bound SOD1-CCS structure[38] and a Zn^{2+}-unbound SOD1-CCS model, derived via rigid-body docking of individual SOD1[39] and CCS[40] crystal structures (see material and methods). In both SOD1-CCS docked models, we found residues in SOD1's GDNT motif (positions 52–55) to be involved in CCS binding as previously reported.[38] We additionally identified a "Zn^{2+}-SOD1-CCS" set of five residues (see Figure 3C) as being involved in binding for the Zn^{2+}-bound SOD1-CCS structure but not the Zn^{2+}-unbound SOD1-CCS structure, suggesting their transient involvement in CCS binding. Both GDNT and the Zn^{2+}-SOD1-CCS residue set were found to be generally intolerant to substitutions in our activity map (Figure 3C). Four of the five Zn^{2+}-SOD1-CCS residues also exhibited tolerance for substitution in our abundance map. Because CCS shares over 50% sequence identity with SOD1,[82] we might expect a CCS-SOD1 binding mode that closely resembles that of the SOD1 homodimer. However, only 13 of the 18 residues at the SOD1 homodimer interface are seen at the SOD1-CCS binding interface. Among these 13 "both-interface" residues, the activity map identified six as intolerant to substitution—including three of the GDNT residues (Gly52, Asp53, and Thr55) and the GDNT-adjacent Phe51 residue—while the abundance map implicated only Ile114 and Gly115 as being important. One possible explanation for the discordance between the maps is the strict requirement for CCS1-dependent SOD1 metal binding in yeast, while a CCS-independent pathway is sufficient for SOD1 metal binding in mammals.[83,84]

Exploring mechanisms of active-site-adjacent substitutions

One way to gain insight into variant mechanism is to generate predictions of protein thermodynamic stability ($\Delta\Delta G$) for each variant. Agreement with functional scores immediately suggests that the functional impact is via a loss-of-stability mechanism. By contrast, "stable-but-inactive" variants—defined by having a functional impact that cannot be explained by an impact on stability—are likely to act by other mechanisms. Across a range of proteins, stable-but-inactive variants have been found to be substrate binding sites, surrounding "second-shell" residues that influence these sites,[26,85,86] or to be important for protein dynamics.[26,85,86] Not surprisingly, $\Delta\Delta G$ scores showed significant (albeit modest) correlation with functional scores (Figures S11A and S11B; Spearman's R for activity = 0.31, $p < 2e-16$; for abundance, R = 0.29,

$p < 2e{-}16$). To identify protein regions enriched for stable-but-inactive variants, we performed moving-window analyses across all protein positions, which compared the mean activity or mean abundance map score in each window with the mean predicted $\Delta\Delta G$ values (Figure S11C).

Comparing the median functional scores and median $\Delta\Delta G$ values at each position identified Gly86, Asn87, and Gly128 as important for enzymatic function but not stability (Figure S12A; teal positions). The positions of these residues within reported SOD1 structures, proximal to known active-site residues Asp125, His47, and His72, suggests that they are second-shell residues with potential to influence catalytic activity without altering stability. Indeed, two substitutions at Asn87—p.Asn87Lys and p.Asn87Ser—are known to be pathogenic with an unknown mechanism.[87] Interestingly, Asn87 is proximal to Asp125, at which a pathogenic variant (p.Asp125Gly) has also been observed. Based both on shared proximity and disease impact, we hypothesized both that Asn87 forms a salt bridge with Asp125, stabilizing the local electrostatic field, and further that this salt bridge facilitates a hydrogen-bond network with the metal-binding residues His47 and His72.

To investigate both of these hypotheses, we carried out 400-ns MD simulations for WT SOD1 and two variants—p.Asn87Lys and p.Gly128Pro (Figure S12B). Previous studies used MD simulations to conclude that folding of the electrostatic loop excludes water and promotes stabilizing hydrogen bonds between Asp125, His47, and His72, together enabling SOD1 to bind both Cu^{2+} and Zn^{2+} ions.[88] The position of the electrostatic loop can be interpreted through hydrogen bonding of Asp125–His47 (indicating amenability to Cu^{2+} binding) and Asp125–His72 (indicating amenability to Zn^{2+} binding).[88] To quantify the relationship between the Asn87-Asp125 interaction and electrostatic loop position, we evaluated Cα distances for Asn87–Asp125 (Figure S12C) and both of the residue pairs that indicate amenability to metal binding (Figures S12D and S12E).

In MD simulations of WT SOD1, the Asn87–Asp125 distance remained consistent with a salt bridge (Figures S12B and S12C). These simulations also showed evidence of hydrogen bonding between Asp125–His47 and Asp125–His72 (22% and 98% of the simulation time, respectively), reflecting occasional Cu^{2+}- and stable Zn^{2+}-binding ability, respectively (Figures S12B, S12F, and S12G). For the p.Asn87Lys variant, MD simulations showed an increased Cα distance for Asn87–Asp125, inconsistent with a salt bridge (Figures S12B and S12C). We also observed a reduction in time spent hydrogen bonded (53%; Figure S12G) for Asp125–His72, as well as elevated MSF values near His72 and within the electrostatic loop (Figure S12H), suggesting an impaired Zn^{2+}-binding state and diminished enzymatic activity.

For the stable-but-inactive candidate second-shell residue Gly128, we tested the hypothesis that this residue provides the flexibility needed for Asp125 to interact with the metal-binding sites by evaluating the movements of the p.Gly128Pro variant in the same set of simulations. In MD simulations of the p.Gly128Pro variant, we saw a weakened Asn87–Asp125 electrostatic interaction (Figure S12B). Interestingly, unlike the p.Asn87Lys variant, the p.Gly128-Pro structure displayed increased MSF values only within the electrostatic loop (Figure S12H). More importantly, as observed for p.Asn87Lys, p.Gly128Pro was conducive to Zn^{2+} binding (46% of the simulation time based on Asp125–His72 hydrogen bonding; Figure S12G).

Taken together, these MD simulation results (summarized in Table S1) (1) suggest that Asn87 binding to Asp125 forms a salt bridge that plays an important role in the positioning of the electrostatic loop and thus binding of both Cu^{2+} and Zn^{2+} ions; and (2) support a previous suggestion that Gly128 introduces steric restrictions in the backbone of the electrostatic loop that create a barrier to the formation of metal-binding sites,[88] providing a potential pathomechanism for variants found at position Asn87.

Functional scores suggest genotype-phenotype association in SOD1-ALS

The impact of a given variant can manifest differently in different people—i.e., can have variable expressivity or incomplete penetrance—due, for example, to environmental influences that can include pathogen exposure, age, sex, and other factors. This variability impacts disease phenotypes, such as earlier disease onset or duration.[35,89,90]

We further investigated two pathogenic variants, p.Asp12Tyr and p.His47Arg, which are considered clinically mild in the sense that onset of ALS is late and progression is slow.[91–93] While p.Asp12Tyr was non-damaging in both maps, p.His47Arg was non-damaging in the abundance map and found damaging in the activity map (Figure S10). The His47 residue, which binds Cu^{2+} directly, is both indirectly important for Zn^{2+} binding and required for SOD1 activity.[94] However, Cu^{2+} can still bind p.His47Arg variant SOD1 (at an alternative Cys112 site),[95] potentially explaining why p.His47Arg is stable in our abundance assay despite loss of enzymatic activity.

We next evaluated correlation more generally between our map scores and a previously assembled collection[35] of molecular and ALS disease phenotypes, including enzymatic activity and abundance, protein half-life (of the human protein measured in mouse), and age of onset and duration of ALS disease. We saw significant correlation with SOD1 half-life for our abundance scores (Figure 4A; $R = 0.78$, $p_{adj} = 2e{-}2$) but not for our activity map scores ($p_{adj} = 1$). We did not find significant correlation between either map and previous measurements of enzyme specific activity (Figure 4B; activity map $p_{adj} = 1$; abundance map $p_{adj} = 3e{-}1$) or with SOD1 protein abundance measurements (Figure 4C; activity map $p_{adj} = 1e{-}1$; abundance map $p_{adj} = 1$). However, scores for our abundance (but not activity map) showed significant correlation with age of onset (Figure 4D; activity map $p_{adj} = 2e{-}1$;

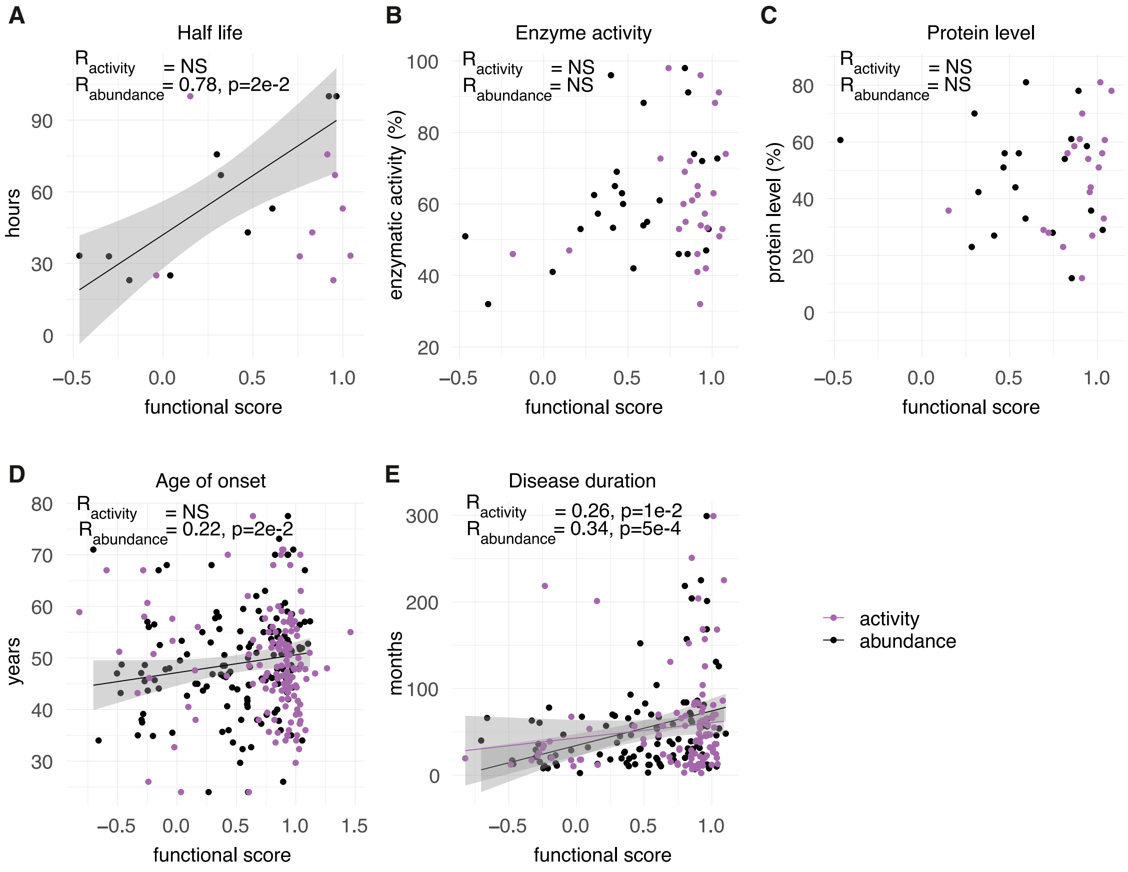

Figure 4. Genotype-phenotype analyses of SOD1 variants in both assays against a dataset of various phenotypes
Scores for both activity and abundance maps (mean score for substitutions at each position) were compared with an assembled dataset phenotypes from people with ALS, including (A) protein half-life measurements (in mice), (B) enzyme specific activity, (C) protein abundance measurements, (D) age of ALS onset, and (E) disease duration.[35] NS, not significant. Gray indicates 95% confidence interval for significant correlations.
See material and methods for details on regression and significance calculations.

abundance map $R = 0.22$, $p_{adj} = 2e{-}2$), while scores from both maps correlated significantly with disease duration (Figure 4E; activity map $R = 0.26$, $p_{adj} = 1e{-}2$; abundance map $R = 0.34$, $p_{adj} = 5e{-}4$).

Taken together, while these analyses revealed only limited correlation between our maps and previous biochemical data, they showed correlation with disease phenotypes, supporting both the quality of the maps and the existence of quantitative genotype-phenotype associations for classical ALS.

SOD1 abundance scores correlate with variant pathogenicity

Next, we wished to assess the ability of the SOD1 maps to identify pathogenic variants. More specifically, at a series of score thresholds for each map, we wished to evaluate precision (fraction of variants below a given threshold score that have been annotated as P/LP) and recall (fraction of all variants annotated as P/LP that scored below that threshold). Because precision is impacted by the proportion of pathogenic variants in the reference set (which will not generally correspond to the proportions observed clinically), we adopted the "balanced precision" measure, which estimates the precision that would have been observed with a balanced (i.e., 50% P/LP) reference set (see material and methods).

To enable this analysis, we established two positive reference sets of P/LP variants: 41 high-confidence variants from ClinVar classified by various submitters[12] and 84 classified by Labcorp Genetics (40 of which were also in the ClinVar set) using their Sherloc system.[12,27] Obtaining a negative reference set was more problematic due to there only being a single SOD1 missense variant that has been annotated as benign or likely benign (p.Asn20Ser defined as benign in the Labcorp set).[12,96] We therefore selected 100 PB variants from gnomAD[96] that have no annotated disease association or evidence of functional impact. We note that, to the extent that our negative and pathogenic reference sets have been contaminated with truly pathogenic and benign variants, respectively, our performance estimates will tend to be conservatively low. We also note that all analyses were carried out using the subset of reference-set variants having scores in both maps and from all computational predictors examined. The final reference sets contained 41 P/LP and 93 PB (ClinVar/gnomAD) and 83 P/LP and 94 B/PB (Labcorp/gnomAD).

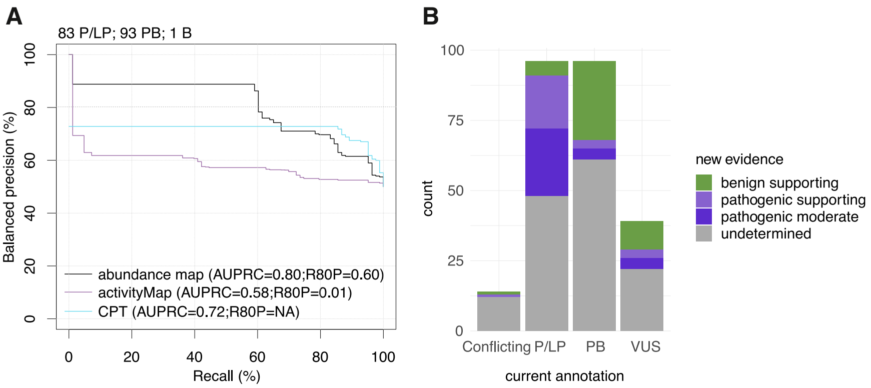

Figure 5. The SOD1 abundance map best distinguishes positive from negative reference variants and provides evidence for 41% of VUSs

(A) Evaluation of precision (fraction of variants in the positive reference set that scored below each threshold functional score) vs. recall (fraction of positive reference variants with functional scores below threshold). More specifically, we used balanced precision such that precision values reflect performance in a setting where positive and negative sets contain the same number of variants for reference sets with variants obtained from Labcorp/gnomAD. Balanced precision vs. recall curves are shown for the SOD1 total enzymatic activity (purple) and abundance maps (black), as well as for the best-performing computational predictor (CPT; turquoise). Performance for three additional predictors is shown in Figure S13. Performance was summarized in terms of area under the balanced precision vs. recall curve (AUBPRC) and recall at a balanced precision of 80% (R80BP). Plot indicates the number of variants in the positive (P/LP), gnomAD negative (PB), and Labcorp negative (B) reference sets used here (see material and methods for details).
(B) Currently clinically annotated variants and new evidence proposed based on calibrating our abundance map scores against the Labcorp Genetics reference sets.
See main text and material and methods for details on the derivation of evidence strength.

Evaluating the performance of the maps using, first, a single threshold score of 0.5 for both activity and abundance maps, we captured 12% and 46% of ClinVar pathogenic variants and 12% and 41% of Labcorp pathogenic variants, respectively (see Table S2 for summary and Data S2 for detailed list of variants). This score threshold achieved a balanced precision of 61% and 88%, respectively, for activity and abundance maps (ClinVar/gnomAD reference). Balanced precision measures for activity and abundance maps using the Labcorp/gnomAD reference were 62% and 89%, respectively. We also examined how performance changed as this threshold was varied. Using the ClinVar/gnomAD and Labcorp/gnomAD reference sets in turn, we derived balanced precision vs. recall curves for both our activity and abundance maps (Figures 5 and S13). For each analysis, we obtained two summary measures of performance: area under the balanced precision vs. recall curve (AUBPRC) and recall at 80% balanced precision (R80BP). For the activity map, this analysis showed AUBPRC values of 0.58 for both reference sets. By contrast, the abundance map showed higher AUBPRC values of 0.79 and 0.80. While the activity map showed poor R80BP performance (2% and 1% for ClinVar and Labcorp sets, respectively), the abundance map showed R80BP performance measures of 56% and 60% (Figures 5A and S13).

Under current clinical interpretation guidelines,[13] evidence from functional assays and computational variant-effect predictors is treated independently so that these sources of evidence are synergistic rather than in opposition. However, it can be still instructive to compare the performance of experimental variant-effect maps with computational predictors. Our abundance map outperformed four widely used predictors—AlphaMissense[97] (Figure S13; AUBPRC = 0.71; 0.72 for ClinVar and Labcorp reference sets, respectively), ESM-1b[98] (AUBPRC = 0.72 and 0.69), cross-protein transfer (CPT)[99] (AUBPRC = 0.73 and 0.72), and VARITY[29] (AUBPRC = 0.68 and 0.72). AUBPRC values and R80BP values are summarized in Table S3. A moving-window analysis did not reveal any SOD1 subregion for which computational predictors showed substantially better (or worse) performance (Figures S14).

To provide our map in a form that is convenient for use in interpreting clinical SOD1 missense variants, we used an established kernel-density estimation method[32,100] to transform all abundance map scores into log likelihood ratios of pathogenicity (LLRp; see material and methods). This calibration used the Labcorp Genetics positive and negative reference sets (augmented with gnomAD PB variants as described; Figure S15A). To calibrate these scores for use within the ACMG/AMP framework for variant interpretation, categorical evidence-strength levels were then derived from LLRp values as previously described.[26,32] Although we also performed this analysis on the activity map, this did not support the assignment of evidence at even the lowest "supporting" strength for any variant (Figure S15B). Although these estimates for evidence strength were obtained using an objective empirical framework, we caution that this procedure has not yet been reviewed by the appropriate ClinGen variant curation expert panel or equivalent clinical body. That said, of the 41 SOD1 missense variants currently annotated as VUSs (Figure 5B), the abundance map provided scores for 39 of them and yielded supporting evidence toward benign for 10 (24%), supporting evidence toward pathogenic for 3 (7%), and moderate evidence toward pathogenic for 4 (10%). Of the 2,387 missense variants with no current ClinVar annotation, the abundance map provided supporting evidence toward benign for 21%, supporting evidence toward pathogenic for 13%, and moderate evidence toward pathogenic for 21%. Thus, our map provides new evidence for over

41% of existing VUSs and 55% of missense variants not yet seen in the clinic, offering the potential to improve sensitivity and accuracy of SOD1 clinical variant classification.

Discussion

Using a combination of functional assays that respectively reflect total enzymatic activity and protein abundance, we generated proactive variant-effect maps evaluating the functional impact of nearly all missense variants of human SOD1.

Functional scores for both maps largely recapitulated known sequence-structure-function relationships for SOD1. For example, buried positions scored significantly lower than non-interfacial surface positions on both maps. Additionally, substitutions at hydrophobic positions were significantly more damaging to both activity and abundance, while conservative substitutions at uncharged sites were better tolerated. Moreover, proline substitutions were generally damaging in both maps, aligning with proline's known role in disrupting secondary structure. In addition to these patterns of mutational tolerance, we found missense variations to be damaging at positions crucial for metal ion (Zn^{2+} and Cu^{2+}) binding and the formation of the Cys58–Cys147 disulfide bond. Variation at positions of residues required for Cu-dependent CCS-mediated activation of SOD1 (i.e., Cu^{2+} insertion and intra-subunit disulfide-bond formation) were well tolerated in our human cell-based abundance map, aligning with previous reports of a CCS-independent pathway of SOD1 activation in human cells.[101] These positions were relatively intolerant to variation in the yeast-based activity map, consistent with the importance of CCS-dependent SOD1 maturation in yeast.[84]

Our maps provide supporting evidence for two existing hypotheses. First, the maps showed that introduction of negatively charged amino acids tended to be detrimental, consistent with the hypothesis that such substitutions increase the toxic, soluble form of SOD1 and correlate with reduced survival in people with SOD1-ALS variants.[67] Second, our maps showed that substitutions in charged residues on the edges of β sheets (Glu22, Asp91, and Glu101), tended to be damaging, supporting a previous hypothesis that positioning of charged residues at β sheet edges decreases aggregation propensity.[80] Although primarily cytosolic, SOD1 is also secreted. SOD1 variants reportedly require a certain degree of solubility for aggregation and prion-like propogation.[102] It would be interesting and clinically valuable to investigate impacts of Glu22, Asp91, and Glu101 on initiating aggregation or prion-like propagation of misfolded SOD1.

Our map scores, supported by MD simulations, provide evidence of a loss-of-function pathogenic mechanism for the p.Asn87Lys variant. Specifically, the interaction between Asn87 and Asp124 (within the electrostatic loop) stabilizes a closed conformation that excludes solvent from the metal site and enhances Zn^{2+} and Cu^{2+} affinity by maintaining strong hydrogen bonds between Asp124 and residues His46 and His72. Consistent with previous reports,[68] our MD simulations also supported a hypothesis that substitutions at Gly128 alter electrostatic loop flexibility by disrupting its proper positioning, increasing solvation, reducing interaction with the Zn^{2+}-binding residue His72, and impairing overall protein stability. Further *in vitro* enzymatic assays and agitation-induced fibrillation studies could validate these findings by testing whether the Asn87–Asp124 interaction governs electrostatic loop flexibility—e.g., our results predict that the pathogenic p.Asn87Lys variant impairs enzymatic activity. Interestingly, for the annotated pathogenic variants p.Asn87Lys and p.Asn87Ser, Phialomustin-B has been proposed as a therapeutic candidate, binding near the electrostatic loop and stabilizing the local conformation necessary for SOD1 function.[103] We did not specifically investigate the role of the inactive-but-stable candidate second-shell residue Gly86. However, given that this position is adjacent to Asn87 and that glycine is known to provide flexibility, we speculate that Gly86 provides the necessary conformational flexibility for Asn87's role.

Our abundance map scores were significantly correlated with disease duration and age of onset in people with SOD1-ALS variants, supporting a genotype-phenotype relationship. Unexpectedly, abundance map scores did not correlate with SOD1 protein-abundance measurements in people with SOD1-ALS despite showing strong positive correlation with mouse protein half-life[35,104]; low SOD1 protein half-life has been attributed to increased chaperone-assisted autophagy, leading to chaperone exhaustion, higher levels of misfolded SOD1, and ALS onset.[105,106] A possible explanation for the low correlation of the abundance map with human/mouse abundance measurements is homeostatic upregulation of SOD1 expression in people with SOD1-ALS variants, particularly in nervous-system tissue.[107,108] Although such homeostasis could compensate for reduced protein half-life *in vivo*, we should not expect to observe this in the exogenous (TetOn) expression system we used for our assays. Support for genotype-phenotype concordance was also demonstrated by variants p.Asp12Tyr and p.His47Arg—deemed pathogenic by ACMG/AMP evidence criteria but associated with a mild clinical course of ALS characterized by late onset and slow progression[53]–which were found to be tolerated in our abundance map. With the increasing routine use of clinical genetic testing, future studies could explore whether abundance impact scores are predictive of disease severity and, more specifically, whether high abundance correlates with clinical benignity.

Our maps revealed that residues at the homodimerization interface are highly sensitive to variation, supporting the idea that dimerization is required for SOD1 activity.[109] That our abundance map reflects the importance of abundance to disease is supported by: the ~0.1 nM K_d for SOD1

dimer dissociation,[110] the significant correlation observed between our abundance map scores and reported protein half-life measurements, and the ability of the abundance map to identify pathogenic variants. Indeed, maintaining lower concentrations of the SOD1 monomer has been shown to prevent its prion-like propagation.[111]

Our variant-effect map scores correlated with pathogenicity, as measured via precision-vs.-recall analysis. The best performance came from the abundance map, surpassing that of top-performing computational predictors such as AlphaMissense, CPT, ESM-1b, and VARITY.

An important limitation of our analysis is that no clinical missense variants of SOD1 are currently classified as benign, so that our negative reference set was necessarily composed of PB variants from gnomAD. Moreover, such contamination should similarly impact performance of both our map and computational predictors, except perhaps to the extent that computational predictors have been overfitted to the pathogenicity labels of variants used in our reference sets.

Another caveat of our study is that measurements were subject to random error. For example, a minority of nonsense variants that had been identified as damaging in yeast-based assays were not detected as damaging in the human-cell-based VAMP-seq assay. Although we used previously described methods[24] to evaluate random errors associated with each experimental score, reflecting the estimated magnitude of random experimental error, this does not prevent random errors that move in the same direction for two replicates for some small subset of variants.

Some measurements may have also been influenced by systematic errors. For instance, a clone may include an additional deleterious variant outside the sequenced tile. That each observed variant is present in many (>50 on average) independent clones will tend to mitigate these effects of secondary, unseen variants within any given clone. Fortunately, the strong separation of scores for synonymous and nonsense variants in both libraries suggests that influences on any given variant's score by unseen distal variants do not represent a major issue.

Both activity and abundance assays had other limitations. Our activity map evaluated protein function within the context of a yeast cell. As noted above, the yeast assay may have erred in finding residues as intolerant to substitution because of their role in facilitating CCS-dependent SOD1 maturation in yeast, given the CCS-independent nature of SOD1 maturation in human cells. In addition, the yeast-based activity map failed to detect the functional impact of many variants outside the active site, including some variants previously shown to induce unstable (yet active) SOD1 that also yield toxic gain of function (aggregation) typical of SOD1-ALS. We speculate that the yeast chaperone Hsp104, which lacks a mammalian or vertebrate ortholog, may have restored solubility of some mutant SOD1 protein, thereby increasing activity and cell growth in our yeast-based assay.[112,113] A caveat of our abundance assay is that it relies on the fusion of SOD1 to GFP, which may slightly alter physicochemical properties of the protein.[114]

We also acknowledge that our observation of a variant being deleterious—whether in the context of the activity or abundance assay—does not, by itself, provide a full mechanistic explanation. Misfolding of SOD1 variants contributes to toxicity through multiple, potentially overlapping pathogenic pathways: gain-of-function pathogenic variants promote toxic oligomeric assemblies but also contribute to degradation that limits the toxicity of these assemblies. Loss-of-function impacts (on abundance, activity, or both) can reduce the enzyme's protective role of limiting damage from free radicals in the nucleus.[115,116] For example, misfolding could promote toxic oligomeric assemblies formed by gain-of-function pathogenic variants but also contribute to degradation that limits the toxicity of these assemblies. Loss-of-function impacts (on either abundance, activity, or both) could reduce the enzyme's protective role of limiting damage from free radicals in the nucleus.[115,116] A more complete set of SOD1-related ALS pathomechanisms should also further include excitotoxicity, oxidative stress, mitochondrial dysfunction, and altered Ca^{2+} metabolism.[116] Both gain- and loss-of-function-associated impacts of misfolding SOD1 variants have been linked to the prion-like propagation along neuroanatomical pathways.[117,118] Although our abundance map provides evidence to identify pathogenic variants, future studies might assess the cell-non-autonomous ability of SOD1 variants to initiate or sustain prion-like propagation of misfolded conformations across a broad neuronal lineage. In the future, additional scalable assays could be employed to generate an atlas of SOD1 variant effects covering pathomechanism beyond activity and abundance.

A further limitation of our maps is their inability to detect aggregation (except indirectly to the extent that it impairs activity or abundance). SOD1 variants associated with toxic gain-of-function aggregation in cell lines have been linked to the accumulation of non-native oligomers, particularly the metastable SOD1 trimer.[115] Aggregates of SOD1 have also been widely reported in samples from people with ALS and animal and cell models of the disease.[116] However, the reported aggregating phenotype of SOD1 variants in cell-based models can be attributed to overexpression, leading to spontaneous non-native folding intermediates, stoichiometric exhaustion of the chaperone pool, or both. This is supported by our failure to observe robust aggregation except in the context of both transient overexpression of WT or p.Ala5Val, p. Gly86Arg variants of SOD1 and treatment with a protease or heat shock protein 70 inhibitor. For cells stably expressing variants, known gain-of-function variants showed reduced scores in our abundance map, suggesting the degradation of misfolded SOD1.[119] Given the genetic heterogeneity of ALS, further studies should focus on the gain-of-function effects of SOD1 variants, especially in

the context of impairment of molecular chaperones, which are important not only for re-folding but also for promoting the degradation of misfolded proteins.[119]

It would be interesting to explore other cellular contexts with potential to modulate SOD1 variant effects. These include oxidative stress, which has been suggested to affect the propensity of SOD1 to aggregate.[120,121] Although, in pilot studies, we found the aggregation propensity of SOD1 variants to be indistinguishable from WT, a human-cell-based complementation assay with the potential to capture more tissue-specific mechanisms might increase sensitivity to aggregation. It would also be interesting to investigate potentially disease-accelerating functional effects of variants in neuronal cell models.

The SOD1 variant-effect maps we provide could have immediate value: for a symptomatic person with a SOD1 variant that would otherwise be classified as VUS, our functional evidence could help confirm an early ALS diagnosis that was otherwise lacking definitive support, thus enabling therapy that is earlier therapeutic intervention that is more tailored to SOD1-ALS. Identification of each additional pathogenic variant could also enable cascade screening to identify family members who are carriers, enabling genetic counseling and surveillance. In each of these scenarios, identification of a definitively classified loss-of-function *SOD1* variant could support the diagnosis of motor neuron disease (e.g., iSODDES) in homozygous individuals. For compound-heterozygous carriers, knowledge of the functional impact of both variants can guide treatment decisions (e.g., suggesting that silencing one of the *SOD1* alleles may be a riskier therapeutic strategy). Knowledge of loss- as well as gain-of-function impacts enables future work evaluating the extent to which loss-of-function impacts will modify the ALS disease course.

Beyond the immediate clinical benefits of offering robust evidence for stronger interpretation and reclassification of variants, these variant-effect maps also offer a resource for deeper insights into sequence-function relationships for SOD1, contributing to the growing atlas of variant effects.[122]

Data and code availability

Final map scores for both maps and abundance-map LLRp values with confidence intervals and ACMG-compatible evidence strengths are available in Data S2. Final scores are also available on MaveDB[123] for the both the abundance map including LLRp values and evidence weights (accession number: urn:mavedb:mavedb:00001217-a-3) and the activity map (accession number: urn:mavedb:00001217-a-4). Code used for analysis can be found on GitHub (https://github.com/axakova/SOD1_Manuscript).

Acknowledgments

We gratefully acknowledge funding for this project from Biogen Inc. We further acknowledge support from the National Institutes of Health National Human Genome Research Institute (NIH/NHGRI) Center of Excellence in Genomic Science Initiative (HG010461), the NIH/NHGRI Impact of Genomic Variation on Function (IGVF) initiative (UM1HG011989), and a Canadian Institutes of Health Research (CIHR) Foundation Grant (FDN-159926) to F.P.R. We thank G. Lum and A. Nasrabad for computational assistance related to MD simulations.

Declaration of interests

F.P.R. is an investor in Ranomics, Inc. and is an investor in and advisor for SeqWell, Inc., BioSymetrics, Inc., and Constantiam Biosciences, Inc. J.H., A.A.M., and S.F. are employees and hold stock/stock options in Biogen Inc. P.M.A. has consulted on advisory boards for Biogen Inc, Roche, Arrowhead, Avrion, Mitsubishi Pharma, Regeneron, uniQure, and Orphazyme A/S and reports as a clinical trial principal investigator for AB Science, AL-S Pharma and Lilly, Amylyx Pharmaceuticals, Alexion Pharmaceuticals, Biogen Idec, IONIS Pharmaceuticals, Novartis, Orion Pharma, PTH Pharmaceuticals, Sanofi, uniQure Biopharma, and Zydus Therapeutics.

Supplemental information

Supplemental information can be found online at https://doi.org/10.1016/j.ajhg.2025.08.019.

Received: February 28, 2025
Accepted: August 25, 2025
Published: September 15, 2025

References

1. Johnston, C.A., Stanton, B.R., Turner, M.R., Gray, R., Blunt, A.H.-M., Butt, D., Ampong, M.-A., Shaw, C.E., Leigh, P.N., and Al-Chalabi, A. (2006). Amyotrophic lateral sclerosis in an urban setting: a population based study of inner city London: A population based study of inner city London. J. Neurol. *253*, 1642–1643.
2. Martin, S., Al Khleifat, A., and Al-Chalabi, A. (2017). What causes amyotrophic lateral sclerosis? F1000Res. *6*, 371.
3. Alonso, A., Logroscino, G., Jick, S.S., and Hernán, M.A. (2009). Incidence and lifetime risk of motor neuron disease in the United Kingdom: a population-based study. Eur. J. Neurol. *16*, 745–751.
4. Ryan, M., Heverin, M., Doherty, M.A., Davis, N., Corr, E.M., Vajda, A., Pender, N., McLaughlin, R., and Hardiman, O. (2018). Determining the incidence of familiality in ALS: A study of temporal trends in Ireland from 1994 to 2016. Neurol. Genet. *4*, e239.
5. Pasinelli, P., and Brown, R.H. (2006). Molecular biology of amyotrophic lateral sclerosis: insights from genetics. Nat. Rev. Neurosci. *7*, 710–723.
6. Ruffo, P., Perrone, B., and Conforti, F.L. (2022). SOD-1 Variants in Amyotrophic Lateral Sclerosis: Systematic Re-Evaluation According to ACMG-AMP Guidelines. Genes *13*, 537. https://doi.org/10.3390/genes13030537.
7. Gibson, S.B., Downie, J.M., Tsetsou, S., Feusier, J.E., Figueroa, K.P., Bromberg, M.B., Jorde, L.B., and Pulst, S.M. (2017). The evolving genetic risk for sporadic ALS. Neurology *89*, 226–233.
8. Dave, K.D., Oskarsson, B., Yersak, J., Krauss, R., Heiman-Patterson, T., Lomen-Hoerth, C., Selig, W.K.D., Halpern Paul,

I., Schaeffer, M., Garcia-Trujillo, B., et al. (2024). Contributions of neurologists to diagnostic timelines of ALS and thinkALS as an early referral instrument for clinicians. Amyotroph. Lateral Scler. Frontotemporal Degener. *26*, 215–224.

9. Balendra, R., Jones, A.R., Al Khleifat, A., Chiwera, T., Wicks, P., Young, C.A., Shaw, P.J., Turner, M.R., Leigh, P.N., and Al-Chalabi, A. (2023). Comparison of King's clinical staging in multinational amyotrophic lateral sclerosis cohorts. Amyotroph. Lateral Scler. Frontotemporal Degener *24*, 71–81.
10. Morgan, S., Shoai, M., Fratta, P., Sidle, K., Orrell, R., Sweeney, M.G., Shatunov, A., Sproviero, W., Jones, A., Al-Chalabi, A., et al. (2015). Investigation of next-generation sequencing technologies as a diagnostic tool for amyotrophic lateral sclerosis. Neurobiol. Aging *36*, 1600.e5–1600.e8.
11. Benatar, M., Wuu, J., Andersen, P.M., Bucelli, R.C., Andrews, J.A., Otto, M., Farahany, N.A., Harrington, E.A., Chen, W., Mitchell, A.A., et al. (2022). Design of a randomized, placebo-controlled, phase 3 trial of tofersen initiated in clinically presymptomatic SOD1 variant carriers: The ATLAS study. Neurotherapeutics *19*, 1248–1258.
12. Landrum, M.J., Lee, J.M., Riley, G.R., Jang, W., Rubinstein, W.S., Church, D.M., and Maglott, D.R. (2014). ClinVar: public archive of relationships among sequence variation and human phenotype. Nucleic Acids Res. *42*, D980–D985.
13. Richards, S., Aziz, N., Bale, S., Bick, D., Das, S., Gastier-Foster, J., Grody, W.W., Hegde, M., Lyon, E., Spector, E., et al. (2015). Standards and guidelines for the interpretation of sequence variants: a joint consensus recommendation of the American College of Medical Genetics and Genomics and the Association for Molecular Pathology. Genet. Med. *17*, 405–424.
14. Tabet, D., Parikh, V., Mali, P., Roth, F.P., and Claussnitzer, M. (2022). Scalable Functional Assays for the Interpretation of Human Genetic Variation. Annu. Rev. Genet. *56*, 441–465.
15. Gerasimavicius, L., Livesey, B.J., and Marsh, J.A. (2022). Loss-of-function, gain-of-function and dominant-negative mutations have profoundly different effects on protein structure. Nat. Commun. *13*, 3895.
16. Pejaver, V., Byrne, A.B., Feng, B.-J., Pagel, K.A., Mooney, S. D., Karchin, R., O'Donnell-Luria, A., Harrison, S.M., Tavtigian, S.V., Greenblatt, M.S., et al. (2022). Calibration of computational tools for missense variant pathogenicity classification and ClinGen recommendations for PP3/BP4 criteria. Am. J. Hum. Genet. *109*, 2163–2177.
17. Stenton, S.L., Pejaver, V., Bergquist, T., Biesecker, L.G., Byrne, A.B., Nadeau, E.A.W., Greenblatt, M.S., Harrison, S. M., Tavtigian, S.V., Radivojac, P., et al. (2024). Assessment of the evidence yield for the calibrated PP3/BP4 computational recommendations. Genet. Med. *26*, 101213.
18. Fayer, S., Horton, C., Dines, J.N., Rubin, A.F., Richardson, M.E., McGoldrick, K., Hernandez, F., Pesaran, T., Karam, R., Shirts, B.H., et al. (2021). Closing the gap: Systematic integration of multiplexed functional data resolves variants of uncertain significance in BRCA1, TP53, and PTEN. Am. J. Hum. Genet. *108*, 2248–2258.
19. Scott, A., Hernandez, F., Chamberlin, A., Smith, C., Karam, R., and Kitzman, J.O. (2022). Saturation-scale functional evidence supports clinical variant interpretation in Lynch syndrome. Genome Biol. *23*, 266.
20. Sun, S., Yang, F., Tan, G., Costanzo, M., Oughtred, R., Hirschman, J., Theesfeld, C.L., Bansal, P., Sahni, N., Yi, S., et al. (2016). An extended set of yeast-based functional assays accurately identifies human disease mutations. Genome Res. *26*, 670–680.
21. Luck, K., Kim, D.-K., Lambourne, L., Spirohn, K., Begg, B.E., Bian, W., Brignall, R., Cafarelli, T., Campos-Laborie, F.J., Charloteaux, B., et al. (2020). A reference map of the human binary protein interactome. Nature *580*, 402–408.
22. Brasil, A.d.A., de Carvalho, M.D.C., Gerhardt, E., Queiroz, D.D., Pereira, M.D., Outeiro, T.F., and Eleutherio, E.C.A. (2019). Characterization of the activity, aggregation, and toxicity of heterodimers of WT and ALS-associated mutant Sod1. Proc. Natl. Acad. Sci. USA *116*, 25991–26000.
23. Huai, J., and Zhang, Z. (2019). Structural Properties and Interaction Partners of Familial ALS-Associated SOD1 Mutants. Front. Neurol. *10*, 527.
24. Weile, J., Song, S., Cote, A.G., Knapp, J., Verby, M., Mellor, J. C., Wu, Y., Pons, C., Wong, C., van Lieshout, N., et al. (2017). A framework for exhaustively mapping functional missense variants. Mol. Syst. Biol. *13*, 957.
25. Matreyek, K.A., Starita, L.M., Stephany, J.J., Martin, B., Chiasson, M.A., Gray, V.E., Kircher, M., Khechaduri, A., Dines, J.N., Hause, R.J., et al. (2018). Multiplex assessment of protein variant abundance by massively parallel sequencing. Nat. Genet. *50*, 874–882.
26. van Loggerenberg, W., Sowlati-Hashjin, S., Weile, J., Hamilton, R., Chawla, A., Sheykhkarimli, D., Gebbia, M., Kishore, N., Frésard, L., Mustajoki, S., et al. (2023). Systematically testing human HMBS missense variants to reveal mechanism and pathogenic variation. Am. J. Hum. Genet. *110*, 1769–1786.
27. Nykamp, K., Anderson, M., Powers, M., Garcia, J., Herrera, B., Ho, Y.-Y., Kobayashi, Y., Patil, N., Thusberg, J., Westbrook, M., et al. (2020). Correction: Sherloc: a comprehensive refinement of the ACMG-AMP variant classification criteria. Genet. Med. *22*, 240.
28. Weile, J., Kishore, N., Sun, S., Maaieh, R., Verby, M., Li, R., Fotiadou, I., Kitaygorodsky, J., Wu, Y., Holenstein, A., et al. (2021). Shifting landscapes of human MTHFR missense-variant effects. Am. J. Hum. Genet. *108*, 1283–1300.
29. Wu, Y., Li, R., Sun, S., Weile, J., and Roth, F.P. (2021). Improved pathogenicity prediction for rare human missense variants. Am. J. Hum. Genet. *108*, 1891–1906.
30. Epanechnikov, V.A. (1969). Non-parametric estimation of a multivariate probability density. Theory Probab. Appl. *14*, 153–158.
31. Sheather, S.J., and Jones, M.C. (1991). A reliable data-based bandwidth selection method for kernel density estimation. Journal of the royal statistical society series b-methodological *53*, 683–690.
32. Tavtigian, S.V., Greenblatt, M.S., Harrison, S.M., Nussbaum, R.L., Prabhu, S.A., Boucher, K.M., Biesecker, L.G.; and ClinGen Sequence Variant Interpretation Working Group ClinGen SVI (2018). Modeling the ACMG/AMP variant classification guidelines as a Bayesian classification framework. Genet. Med. *20*, 1054–1060.
33. Montanucci, L., Capriotti, E., Frank, Y., Ben-Tal, N., and Fariselli, P. (2019). DDGun: an untrained method for the prediction of protein stability changes upon single and multiple point variations. BMC Bioinf. *20*, 335.
34. Strange, R.W., Antonyuk, S., Hough, M.A., Doucette, P.A., Rodriguez, J.A., Hart, P.J., Hayward, L.J., Valentine, J.S.,

and Hasnain, S.S. (2003). The structure of holo and metal-deficient wild-type human Cu, Zn superoxide dismutase and its relevance to familial amyotrophic lateral sclerosis. J. Mol. Biol. *328*, 877–891.
35. Huang, M., Liu, Y.U., Yao, X., Qin, D., and Su, H. (2024). Variability in SOD1-associated amyotrophic lateral sclerosis: geographic patterns, clinical heterogeneity, molecular alterations, and therapeutic implications. Transl. Neurodegener. *13*, 28.
36. Keskin, I., Forsgren, E., Lange, D.J., Weber, M., Birve, A., Synofzik, M., Gilthorpe, J.D., Andersen, P.M., and Marklund, S.L. (2016). Effects of cellular pathway disturbances on misfolded superoxide dismutase-1 in fibroblasts derived from ALS patients. PLoS One *11*, e0150133.
37. Synofzik, M., Ronchi, D., Keskin, I., Basak, A.N., Wilhelm, C., Gobbi, C., Birve, A., Biskup, S., Zecca, C., Fernández-Santiago, R., et al. (2012). Mutant superoxide dismutase-1 indistinguishable from wild-type causes ALS. Hum. Mol. Genet. *21*, 3568–3574.
38. Sala, F.A., Wright, G.S.A., Antonyuk, S.V., Garratt, R.C., and Hasnain, S.S. (2019). Molecular recognition and maturation of SOD1 by its evolutionarily destabilised cognate chaperone hCCS. PLoS Biol. *17*, e3000141.
39. Banci, L., Bertini, I., Cramaro, F., Del Conte, R., and Viezzoli, M.S. (2003). Solution structure of Apo Cu,Zn superoxide dismutase: role of metal ions in protein folding. Biochemistry *42*, 9543–9553.
40. Lamb, A.L., Wernimont, A.K., Pufahl, R.A., O'Halloran, T. V., and Rosenzweig, A.C. (2000). Crystal structure of the second domain of the human copper chaperone for superoxide dismutase. Biochemistry *39*, 1589–1595.
41. Jones, G., Jindal, A., Ghani, U., Kotelnikov, S., Egbert, M., Hashemi, N., Vajda, S., Padhorny, D., and Kozakov, D. (2022). Elucidation of protein function using computational docking and hotspot analysis by ClusPro and FTMap. Acta Crystallogr. D Struct. Biol. *78*, 690–697.
42. Desta, I.T., Porter, K.A., Xia, B., Kozakov, D., and Vajda, S. (2020). Performance and its limits in rigid body protein-protein docking. Structure *28*, 1071–1081.e3.
43. Vajda, S., Yueh, C., Beglov, D., Bohnuud, T., Mottarella, S.E., Xia, B., Hall, D.R., and Kozakov, D. (2017). New additions to the ClusPro server motivated by CAPRI. Proteins *85*, 435–444.
44. Kozakov, D., Hall, D.R., Xia, B., Porter, K.A., Padhorny, D., Yueh, C., Beglov, D., and Vajda, S. (2017). The ClusPro web server for protein-protein docking. Nat. Protoc. *12*, 255–278.
45. Kozakov, D., Beglov, D., Bohnuud, T., Mottarella, S.E., Xia, B., Hall, D.R., and Vajda, S. (2013). How good is automated protein docking?: Automated Protein Docking. Proteins *81*, 2159–2166.
46. Elez, K., Bonvin, A.M.J.J., and Vangone, A. (2018). Distinguishing crystallographic from biological interfaces in protein complexes: role of intermolecular contacts and energetics for classification. BMC Bioinf. *19*, 438.
47. Jiménez-García, B., Elez, K., Koukos, P.I., Bonvin, A.M., and Vangone, A. (2019). PRODIGY-crystal: a web-tool for classification of biological interfaces in protein complexes. Bioinformatics *35*, 4821–4823.
48. Mitternacht, S. (2016). FreeSASA: An open source C library for solvent accessible surface area calculations. F1000Res. *5*, 189.
49. Strange, R.W., Antonyuk, S.V., Hough, M.A., Doucette, P.A., Valentine, J.S., and Hasnain, S.S. (2006). Variable metallation of human superoxide dismutase: atomic resolution crystal structures of Cu-Zn, Zn-Zn and as-isolated wild-type enzymes. J. Mol. Biol. *356*, 1152–1162.
50. Jo, S., Kim, T., Iyer, V.G., and Im, W. (2008). CHARMM-GUI: a web-based graphical user interface for CHARMM. J. Comput. Chem. *29*, 1859–1865.
51. Lee, H., Radu, C., Han, J.W., and Grailhe, R. (2017). Assay Development for High Content Quantification of Sod1 Mutant Protein Aggregate Formation in Living Cells. J. Vis. Exp. *128*, 56425. https://doi.org/10.3791/56425.
52. Sawamura, M., Imamura, K., Hikawa, R., Enami, T., Nagahashi, A., Yamakado, H., Ichijo, H., Fujisawa, T., Yamashita, H., Minamiyama, S., et al. (2022). Cellular analysis of SOD1 protein-aggregation propensity and toxicity: a case of ALS with slow progression harboring homozygous SOD1-D92G mutation. Sci. Rep. *12*, 12636.
53. Chen, L.-X., Xu, H.-F., Lin, H.-X., Yang, X.-X., Li, H.-F., and Wu, Z.-Y. (2023). Pathogenicity classification of SOD1 variants of uncertain significance by in vitro aggregation propensity. Neurobiol. Aging *123*, 182–190.
54. Pokrishevsky, E., Grad, L.I., and Cashman, N.R. (2016). TDP-43 or FUS-induced misfolded human wild-type SOD1 can propagate intercellularly in a prion-like fashion. Sci. Rep. *6*, 22155.
55. Zu, J.S., Deng, H.X., Lo, T.P., Mitsumoto, H., Ahmed, M.S., Hung, W.Y., Cai, Z.J., Tainer, J.A., and Siddique, T. (1997). Exon 5 encoded domain is not required for the toxic function of mutant SOD1 but essential for the dismutase activity: identification and characterization of two new SOD1 mutations associated with familial amyotrophic lateral sclerosis. Neurogenetics *1*, 65–71.
56. Andersen, P.M., Nordström, U., Tsiakas, K., Johannsen, J., Volk, A.E., Bierhals, T., Zetterström, P., Marklund, S.L., Hempel, M., and Santer, R. (2019). Phenotype in an infant with SOD1 homozygous truncating mutation. N. Engl. J. Med. *381*, 486–488.
57. Saccon, R.A., Bunton-Stasyshyn, R.K.A., Fisher, E.M.C., and Fratta, P. (2013). Is SOD1 loss of function involved in amyotrophic lateral sclerosis? Brain *136*, 2342–2358.
58. Park, J.H., Nordström, U., Tsiakas, K., Keskin, I., Elpers, C., Mannil, M., Heller, R., Nolan, M., Alburaiky, S., Zetterström, P., et al. (2023). The motor system is exceptionally vulnerable to absence of the ubiquitously expressed superoxide dismutase-1. Brain Commun. *5*, fcad017.
59. Park, J.H., Elpers, C., Reunert, J., McCormick, M.L., Mohr, J., Biskup, S., Schwartz, O., Rust, S., Grüneberg, M., Seelhöfer, A., et al. (2019). SOD1 deficiency: a novel syndrome distinct from amyotrophic lateral sclerosis. Brain *142*, 2230–2237.
60. Ratovitski, T., Corson, L.B., Strain, J., Wong, P., Cleveland, D.W., Culotta, V.C., and Borchelt, D.R. (1999). Variation in the biochemical/biophysical properties of mutant superoxide dismutase 1 enzymes and the rate of disease progression in familial amyotrophic lateral sclerosis kindreds. Hum. Mol. Genet. *8*, 1451–1460.
61. Farrawell, N.E., and Yerbury, J.J. (2021). Mutant cu/Zn superoxide dismutase (A4V) turnover is altered in cells containing inclusions. Front. Mol. Neurosci. *14*, 771911.
62. Keskin, I., Birve, A., Berdynski, M., Hjertkvist, K., Rofougaran, R., Nilsson, T.K., Glass, J.D., Marklund, S.L., and Andersen, P.M. (2017). Comprehensive analysis to explain reduced or increased SOD1 enzymatic activity in ALS

patients and their relatives. Amyotroph. Lateral Scler. Frontotemporal Degener. *18*, 457–463.
63. Leykam, L., Forsberg, K.M.E., Nordström, U., Hjertkvist, K., Öberg, A., Jonsson, E., Andersen, P.M., Marklund, S.L., and Zetterström, P. (2024). Specific analysis of SOD1 enzymatic activity in CSF from ALS patients with and without SOD1 mutations. Neurobiol. Dis. *202*, 106718.
64. Zetterström, P., Stewart, H.G., Bergemalm, D., Jonsson, P.A., Graffmo, K.S., Andersen, P.M., Brännström, T., Oliveberg, M., and Marklund, S.L. (2007). Soluble misfolded subfractions of mutant superoxide dismutase-1s are enriched in spinal cords throughout life in murine ALS models. Proc. Natl. Acad. Sci. USA *104*, 14157–14162.
65. Dunham, A.S., and Beltrao, P. (2021). Exploring amino acid functions in a deep mutational landscape. Mol. Syst. Biol. *17*, e10305.
66. Lek, M., Karczewski, K.J., Minikel, E.V., Samocha, K.E., Banks, E., Fennell, T., O'Donnell-Luria, A.H., Ware, J.S., Hill, A.J., Cummings, B.B., et al. (2016). Analysis of protein-coding genetic variation in 60,706 humans. Nature *536*, 285–291.
67. Lindberg, M.J., Byström, R., Boknäs, N., Andersen, P.M., and Oliveberg, M. (2005). Systematically perturbed folding patterns of amyotrophic lateral sclerosis (ALS)-associated SOD1 mutants. Proc. Natl. Acad. Sci. USA *102*, 9754–9759.
68. Mavadat, E., Seyedalipour, B., Hosseinkhani, S., and Colagar, A.H. (2023). Role of charged residues of the "electrostatic loop" of hSOD1 in promotion of aggregation: Implications for the mechanism of ALS-associated mutations under amyloidogenic conditions. Int. J. Biol. Macromol. *244*, 125289.
69. Rakhit, R., and Chakrabartty, A. (2006). Structure, folding, and misfolding of Cu,Zn superoxide dismutase in amyotrophic lateral sclerosis. Biochim. Biophys. Acta *1762*, 1025–1037.
70. Boyd, S.D., Ullrich, M.S., Skopp, A., and Winkler, D.D. (2020). Copper Sources for Sod1 Activation. Antioxidants *9*, 500. https://doi.org/10.3390/antiox9060500.
71. Boyd, S.D., Liu, L., Bulla, L., and Winkler, D.D. (2018). Quantifying the Interaction between Copper-Zinc Superoxide Dismutase (Sod1) and its Copper Chaperone (Ccs1). J. Proteomics Bioinform. *11*, 473. https://doi.org/10.4172/jpb.1000473.
72. Arnesano, F., Banci, L., Bertini, I., Martinelli, M., Furukawa, Y., and O'Halloran, T.V. (2004). The unusually stable quaternary structure of human Cu,Zn-superoxide dismutase 1 is controlled by both metal occupancy and disulfide status. J. Biol. Chem. *279*, 47998–48003.
73. Hart, P.J., Balbirnie, M.M., Ogihara, N.L., Nersissian, A.M., Weiss, M.S., Valentine, J.S., and Eisenberg, D. (1999). A structure-based mechanism for copper-zinc superoxide dismutase. Biochemistry *38*, 2167–2178.
74. Eleutherio, E.C.A., Silva Magalhães, R.S., de Araújo Brasil, A., Monteiro Neto, J.R., and de Holanda Paranhos, L. (2021). SOD1, more than just an antioxidant. Arch. Biochem. Biophys. *697*, 108701.
75. Xu, W.-C., Liang, J.-Z., Li, C., He, Z.-X., Yuan, H.-Y., Huang, B.-Y., Liu, X.-L., Tang, B., Pang, D.-W., Du, H.-N., et al. (2018). Pathological hydrogen peroxide triggers the fibrillization of wild-type SOD1 via sulfenic acid modification of Cys-111. Cell Death Dis. *9*, 67.
76. Müller, K., Oh, K.-W., Nordin, A., Panthi, S., Kim, S.H., Nordin, F., Freischmidt, A., Ludolph, A.C., Ki, C.S., Forsberg, K., et al. (2022). De novo mutations in SOD1 are a cause of ALS. J. Neurol. Neurosurg. Psychiatry *93*, 201–206.
77. DuVal, M.G., Hinge, V.K., Snyder, N., Kanyo, R., Bratvold, J., Pokrishevsky, E., Cashman, N.R., Blinov, N., Kovalenko, A., and Allison, W.T. (2019). Tryptophan 32 mediates SOD1 toxicity in a in vivo motor neuron model of ALS and is a promising target for small molecule therapeutics. Neurobiol. Dis. *124*, 297–310.
78. Wen, X., Zhu, W., Xia, N.L., Li, Q., Di, L., Zhang, S., Chen, H., Lu, Y., Wang, M., Xu, M., et al. (2021). Missense Mutations of Codon 116 in the SOD1 Gene Cause Rapid Progressive Familial ALS and Predict Short Viability With PMA Phenotype. Front. Genet. *12*, 776831.
79. Esteras-Chopo, A., Serrano, L., and López de la Paz, M. (2005). The amyloid stretch hypothesis: recruiting proteins toward the dark side. Proc. Natl. Acad. Sci. USA *102*, 16672–16677.
80. Richardson, J.S., and Richardson, D.C. (2002). Natural beta-sheet proteins use negative design to avoid edge-to-edge aggregation. Proc. Natl. Acad. Sci. USA *99*, 2754–2759.
81. Nordlund, A., and Oliveberg, M. (2006). Folding of Cu/Zn superoxide dismutase suggests structural hotspots for gain of neurotoxic function in ALS: parallels to precursors in amyloid disease. Proc. Natl. Acad. Sci. USA *103*, 10218–10223.
82. Furukawa, Y., and O'Halloran, T.V. (2006). Posttranslational modifications in Cu,Zn-superoxide dismutase and mutations associated with amyotrophic lateral sclerosis. Antioxid. Redox Signal. *8*, 847–867.
83. Carroll, M.C., Girouard, J.B., Ulloa, J.L., Subramaniam, J.R., Wong, P.C., Valentine, J.S., and Culotta, V.C. (2004). Mechanisms for activating Cu- and Zn-containing superoxide dismutase in the absence of the CCS Cu chaperone. Proc. Natl. Acad. Sci. USA *101*, 5964–5969.
84. Boyd, S.D., Calvo, J.S., Liu, L., Ullrich, M.S., Skopp, A., Meloni, G., and Winkler, D.D. (2019). The yeast copper chaperone for copper-zinc superoxide dismutase (CCS1) is a multifunctional chaperone promoting all levels of SOD1 maturation. J. Biol. Chem. *294*, 1956–1966.
85. Cagiada, M., Johansson, K.E., Valanciute, A., Nielsen, S.V., Hartmann-Petersen, R., Yang, J.J., Fowler, D.M., Stein, A., and Lindorff-Larsen, K. (2021). Understanding the origins of loss of protein function by analyzing the effects of thousands of variants on activity and abundance. Mol. Biol. Evol. *38*, 3235–3246.
86. Høie, M.H., Cagiada, M., Beck Frederiksen, A.H., Stein, A., and Lindorff-Larsen, K. (2022). Predicting and interpreting large-scale mutagenesis data using analyses of protein stability and conservation. Cell Rep. *38*, 110207.
87. Hayward, C., Brock, D.J., Minns, R.A., and Swingler, R.J. (1998). Homozygosity for Asn86Ser mutation in the CuZn-superoxide dismutase gene produces a severe clinical phenotype in a juvenile onset case of familial amyotrophic lateral sclerosis. J. Med. Genet. *35*, 174.
88. Mera-Adasme, R., Suomivuori, C.-M., Fierro, A., Pesonen, J., and Sundholm, D. (2013). The role of solvent exclusion in the interaction between D124 and the metal site in SOD1: implications for ALS. J. Biol. Inorg. Chem. *18*, 931–938.
89. Kalia, M., Miotto, M., Ness, D., Opie-Martin, S., Spargo, T.P., Di Rienzo, L., Biagini, T., Petrizzelli, F., Al Khleifat, A., Kabiljo, R., et al. (2023). Molecular dynamics analysis of superoxide dismutase 1 mutations suggests decoupling between

mechanisms underlying ALS onset and progression. Comput. Struct. Biotechnol. J. *21*, 5296–5308.

90. Spargo, T.P., Opie-Martin, S., Hunt, G.P., Kalia, M., Al Khleifat, A., Topp, S.D., Shaw, C.E., Al-Chalabi, A., Iacoangeli, A.; and Project Mine ALS Sequencing Consortium (2023). SOD1-ALS-Browser: a web-utility for investigating the clinical phenotype in SOD1 amyotrophic lateral sclerosis. Amyotroph. Lateral Scler. Frontotemporal Degener. *24*, 1–10.
91. Del Grande, A., Conte, A., Lattante, S., Luigetti, M., Marangi, G., Zollino, M., Madia, F., Bisogni, G., and Sabatelli, M. (2011). D11Y SOD1 mutation and benign ALS: a consistent genotype-phenotype correlation. J. Neurol. Sci. *309*, 31–33.
92. Bayraktar, E., Çiftçi, V., Uysal, H., and Başak, A.N. (2023). Another de novo mutation in the SOD1 gene: the first Turkish patient with SOD1-His47Arg, a case report. Front. Genet. *14*, 1208673.
93. Ohi, T., Saita, K., Takechi, S., Nabesima, K., Tashiro, H., Shiomi, K., Sugimoto, S., Akematsu, T., Nakayama, T., Iwaki, T., and Matsukura, S. (2002). Clinical features and neuropathological findings of familial amyotrophic lateral sclerosis with a His46Arg mutation in Cu/Zn superoxide dismutase. J. Neurol. Sci. *197*, 73–78.
94. Antonyuk, S., Elam, J.S., Hough, M.A., Strange, R.W., Doucette, P.A., Rodriguez, J.A., Hayward, L.J., Valentine, J.S., Hart, P.J., and Hasnain, S.S. (2005). Structural consequences of the familial amyotrophic lateral sclerosis SOD1 mutant His46Arg. Protein Sci. *14*, 1201–1213.
95. Liu, H., Zhu, H., Eggers, D.K., Nersissian, A.M., Faull, K.F., Goto, J.J., Ai, J., Sanders-Loehr, J., Gralla, E.B., and Valentine, J.S. (2000). Copper(2+) binding to the surface residue cysteine 111 of His46Arg human copper-zinc superoxide dismutase, a familial amyotrophic lateral sclerosis mutant. Biochemistry *39*, 8125–8132.
96. Karczewski, K.J., Francioli, L.C., Tiao, G., Cummings, B.B., Alföldi, J., Wang, Q., Collins, R.L., Laricchia, K.M., Ganna, A., Birnbaum, D.P., et al. (2020). The mutational constraint spectrum quantified from variation in 141,456 humans. Nature *581*, 434–443.
97. Cheng, J., Novati, G., Pan, J., Bycroft, C., Žemgulytė, A., Applebaum, T., Pritzel, A., Wong, L.H., Zielinski, M., Sargeant, T., et al. (2023). Accurate proteome-wide missense variant effect prediction with AlphaMissense. Science *381*, eadg7492.
98. Brandes, N., Goldman, G., Wang, C.H., Ye, C.J., and Ntranos, V. (2023). Genome-wide prediction of disease variant effects with a deep protein language model. Nat. Genet. *55*, 1512–1522.
99. Jagota, M., Ye, C., Albors, C., Rastogi, R., Koehl, A., Ioannidis, N., and Song, Y.S. (2023). Cross-protein transfer learning substantially improves disease variant prediction. Genome Biol. *24*, 182.
100. Tavtigian, S.V., Harrison, S.M., Boucher, K.M., and Biesecker, L.G. (2020). Fitting a naturally scaled point system to the ACMG/AMP variant classification guidelines. Hum. Mutat. *41*, 1734–1737.
101. Proescher, J.B., Son, M., Elliott, J.L., and Culotta, V.C. (2008). Biological effects of CCS in the absence of SOD1 enzyme activation: implications for disease in a mouse model for ALS. Hum. Mol. Genet. *17*, 1728–1737.
102. Sugaya, K., and Nakano, I. (2014). Prognostic role of "prion-like propagation" in SOD1-linked familial ALS: an alternative view. Front. Cell. Neurosci. *8*, 359.
103. Unni, S., Kommu, P., Aouti, S., Nalli, Y., Bharath, M.M.S., Ali, A., and Padmanabhan, B. (2024). Structural insights into the modulation Of SOD1 aggregation By a fungal metabolite Phialomustin-B: Therapeutic potential in ALS. PLoS One *19*, e0298196.
104. Ly, C.V., Ireland, M.D., Self, W.K., Bollinger, J., Jockel-Balsarotti, J., Herzog, H., Allred, P., Miller, L., Doyle, M., Anez-Bruzual, I., et al. (2023). Protein kinetics of superoxide dismutase-1 in familial and sporadic amyotrophic lateral sclerosis. Ann. Clin. Transl. Neurol. *10*, 1012–1024.
105. Kepp, K.P. (2015). Genotype-property patient-phenotype relations suggest that proteome exhaustion can cause amyotrophic lateral sclerosis. PLoS One *10*, e0118649.
106. Guan, T., Zhou, T., Zhang, X., Guo, Y., Yang, C., Lin, J., Zhang, J.V., Cheng, Y., Marzban, H., Wang, Y.T., and Kong, J. (2023). Selective removal of misfolded SOD1 delays disease onset in a mouse model of amyotrophic lateral sclerosis. Cell. Mol. Life Sci. *80*, 304.
107. Gagliardi, S., Cova, E., Davin, A., Guareschi, S., Abel, K., Alvisi, E., Laforenza, U., Ghidoni, R., Cashman, J.R., Ceroni, M., and Cereda, C. (2010). SOD1 mRNA expression in sporadic amyotrophic lateral sclerosis. Neurobiol. Dis. *39*, 198–203.
108. Milani, P., Gagliardi, S., Cova, E., and Cereda, C. (2011). SOD1 Transcriptional and Posttranscriptional Regulation and Its Potential Implications in ALS. Neurol. Res. Int. *2011*, 458427.
109. Harris, N., Bachler, M., Costa, V., Mollapour, M., Moradas-Ferreira, P., and Piper, P.W. (2005). Overexpressed Sod1p acts either to reduce or to increase the lifespans and stress resistance of yeast, depending on whether it is Cu(2+)-deficient or an active Cu,Zn-superoxide dismutase. Aging Cell *4*, 41–52.
110. Khare, S.D., Caplow, M., and Dokholyan, N.V. (2004). The rate and equilibrium constants for a multistep reaction sequence for the aggregation of superoxide dismutase in amyotrophic lateral sclerosis. Proc. Natl. Acad. Sci. USA *101*, 15094–15099.
111. Bidhendi, E.E., Bergh, J., Zetterström, P., Andersen, P.M., Marklund, S.L., and Brännström, T. (2016). Two superoxide dismutase prion strains transmit amyotrophic lateral sclerosis-like disease. J. Clin. Investig. *126*, 2249–2253.
112. Kim, Y., Park, J.-H., Jang, J.-Y., Rhim, H., and Kang, S. (2013). Characterization and Hsp104-induced artificial clearance of familial ALS-related SOD1 aggregates. Biochem. Biophys. Res. Commun. *434*, 521–526.
113. Jackrel, M.E., DeSantis, M.E., Martinez, B.A., Castellano, L. M., Stewart, R.M., Caldwell, K.A., Caldwell, G.A., and Shorter, J. (2014). Potentiated Hsp104 variants antagonize diverse proteotoxic misfolding events. Cell *156*, 170–182.
114. Stevens, J.C., Chia, R., Hendriks, W.T., Bros-Facer, V., van Minnen, J., Martin, J.E., Jackson, G.S., Greensmith, L., Schiavo, G., and Fisher, E.M.C. (2010). Modification of superoxide dismutase 1 (SOD1) properties by a GFP tag–implications for research into amyotrophic lateral sclerosis (ALS). PLoS One *5*, e9541.
115. Hnath, B., and Dokholyan, N.V. (2022). Toxic SOD1 trimers are off-pathway in the formation of amyloid-like fibrils in ALS. Biophys. J. *121*, 2084–2095.
116. Peggion, C., Scalcon, V., Massimino, M.L., Nies, K., Lopreiato, R., Rigobello, M.P., and Bertoli, A. (2022). SOD1 in ALS: Taking Stock in Pathogenic Mechanisms and the

Role of Glial and Muscle Cells. Antioxidants *11*, 614. https://doi.org/10.3390/antiox11040614.

117. Ayers, J.I., Fromholt, S.E., O'Neal, V.M., Diamond, J.H., and Borchelt, D.R. (2016). Prion-like propagation of mutant SOD1 misfolding and motor neuron disease spread along neuroanatomical pathways. Acta Neuropathol. *131*, 103–114.
118. Keskin, I., Ekhtiari Bidhendi, E., Marklund, M., Andersen, P. M., Brännström, T., Marklund, S.L., and Nordström, U. (2021). Peripheral administration of SOD1 aggregates does not transmit pathogenic aggregation to the CNS of SOD1 transgenic mice. Acta Neuropathol. Commun. *9*, 111.
119. Wright, G.S.A. (2020). Molecular and pharmacological chaperones for SOD1. Biochem. Soc. Trans. *48*, 1795–1806.
120. Álvarez-Zaldiernas, C., Lu, J., Zheng, Y., Yang, H., Blasi, J., Solsona, C., and Holmgren, A. (2016). Cellular Redox Systems Impact the Aggregation of Cu,Zn Superoxide Dismutase Linked to Familial Amyotrophic Lateral Sclerosis. J. Biol. Chem. *291*, 17197–17208.
121. Furukawa, Y. (2013). Redox environment is an intracellular factor to operate distinct pathways for aggregation of Cu, Zn-superoxide dismutase in amyotrophic lateral sclerosis. Front. Cell. Neurosci. *7*, 240.
122. Fowler, D.M., Adams, D.J., Gloyn, A.L., Hahn, W.C., Marks, D.S., Muffley, L.A., Neal, J.T., Roth, F.P., Rubin, A.F., Starita, L.M., and Hurles, M.E. (2023). An Atlas of Variant Effects to understand the genome at nucleotide resolution. Genome Biol. *24*, 147.
123. Esposito, D., Weile, J., Shendure, J., Starita, L.M., Papenfuss, A.T., Roth, F.P., Fowler, D.M., and Rubin, A.F. (2019). MaveDB: an Open-Source Platform to Distribute and Interpret Data from Multiplexed Assays of Variant Effect. Genome Biol. *20*, 223.

The American Journal of Human Genetics, Volume *112*

Supplemental information

Landscapes of missense variant impact

for human superoxide dismutase 1

Anna Axakova, Megan Ding, Atina G. Cote, Radha Subramaniam, Vignesh Senguttuvan, Haotian Zhang, Jochen Weile, Samuel V. Douville, Marinella Gebbia, Ammar Al-Chalabi, Alexander Wahl, Jason Reuter, Jessica Hurt, Adele A. Mitchell, Stephanie Fradette, Peter M. Andersen, Warren van Loggerenberg, and Frederick P. Roth

Supplemental Note

Preliminary explorations to find an multiplexed assay of SOD1 gain of function effects

ALS-linked SOD1 variants have been reported to confer a gain of function in that they promote the formation of oligomers[1], with toxicity that correlates with the presence of smaller oligomers rather than larger aggregates[2]. Since SOD1-ALS is a dominant disease, we did not knock out the endogenous *SOD1* in HEK293T cells to better recapitulate the endogenous system. Indeed, one pathomechanism that has been implicated in ALS is the potential for wild-type SOD1 to heterodimerize with SOD1 variants and thereby increase the amount of aggregated SOD1[3,4]. Although we showed that human cells bearing SOD1-GFP fusions could reliably identify variants that decrease protein abundance, examination of these cells by fluorescence microscopy did not reveal GFP puncta indicating protein aggregation for either gain of function variants or WT cells under the growth conditions we used initially. We therefore sought alternative conditions that might sensitize cells to this toxic aggregation phenotype, with the hope that we might then use FACS to detect aggregating variants[5]. Previous reports suggested that cells can be sensitized to yield SOD1 variant toxicity either by SOD1 variant overexpression, or by environmental changes that either disrupt calcium homeostasis, trigger calpain or activate neuronal nitric oxide synthase[6,7].

First, to assess whether HEK293T cells for could be sensitive to SOD1 aggregation via its overexpression, we used transient transfection of plasmids with SOD1-GFP (WT; pathogenic variants p.Ala5Val, p.Gly86Arg, p.Arg116Gly which are known to aggregate) downstream of the CMV promoter. This was performed both in our baseline growth condition and after addition of the cysteine protease inhibitor MG-101 (also called ALLN) that has been reported to reduce the processing of misfolded SOD1 and result in a higher aggregation[7]. In these experiments, ALLN treatment did yield more aggregation for p.Ala5Val, p.Gly86Arg and p.Arg116Gly variants than for WT SOD1, but aggregation was observed in fewer than 1% of all cells (Figure S3A). The weakness of this effect, coupled with the fact that our multiplexing strategy is most effective when each cell expresses only a single variant, led us to abandon this sensitization strategy.

Second, we assessed whether SOD1 was able to aggregate when more stably expressed in the HEK293T cell system alone, or with or without treatment with KNK437, a heat shock protein 70 chaperone inhibitor. HEK293T integrant cells bearing SOD1-GFP for multiple alleles, including WT, known-aggregating variants p.Gly86Arg and p.Leu145Phe, as well as p.Gly130Ser (with an unknown propensity to aggregate) were evaluated for aggregation after 24 and 48 hours. Only for KNK437 (and only after 48 hours) did we observe the aggregating phenotype, and this was observed only in <1% of all cells stably expressing pathogenic variants (Figure S3B). Low penetrance of this cellular phenotype, coupled with variable aggregation across replicates suggested that this assay was unsuitable for a large-scale assay. Additional attempts to increase the sensitivity of this assay to aggregation using combinations of exogenous NO donor (GSNO), calcium ionophore (A23187), sorbitol, heat shock, ALLN and KNK437 were unsuccessful (data not shown).

Supplemental Figures

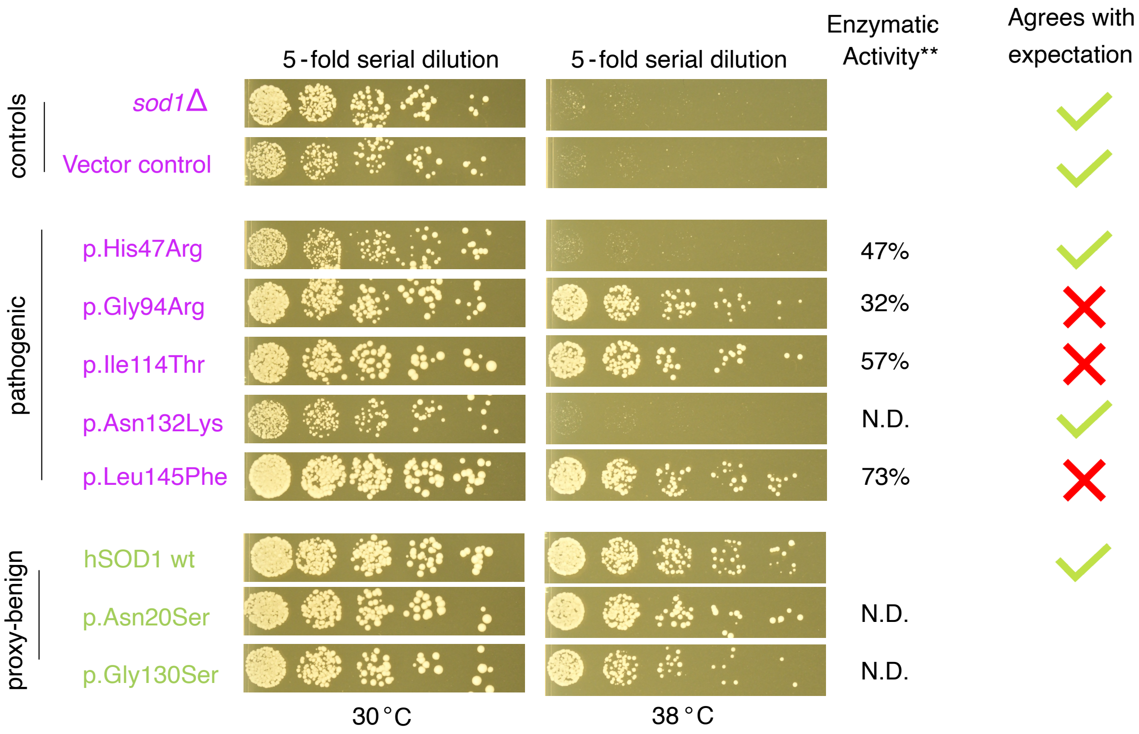

Figure S1: Functional complementation assay results showing whether expression of human SOD1 protein variants can rescue growth of a yeast *sod1Δ* strain.
Complementation assay results for ClinVar-reported negative controls and pathogenic variants (purple text), and WT control and proxy-benign variants (green text). Five-fold serial dilutions of yeast cells were spotted onto plates, with growth evaluation after incubating for 48 hours at either permissive (30°C) or non-permissive (38°C) temperature. Enzymatic activity measurements[8] from individuals with SOD1-ALS variants p.His47Arg, p.Gly94Arg, p.Ile114Thr and p.Leu145Phe are shown.

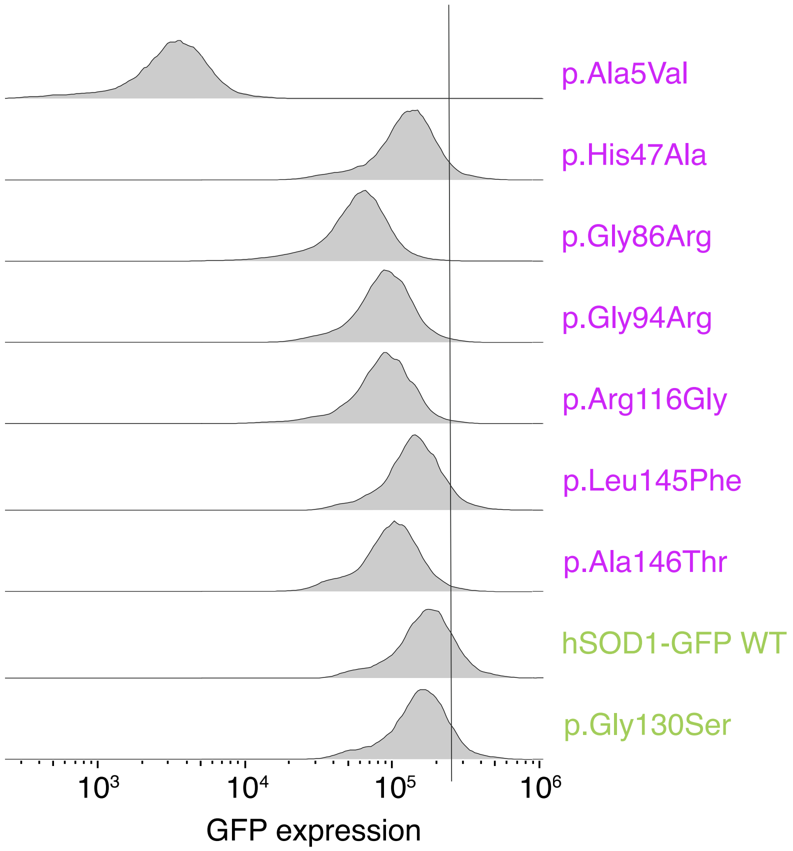

Figure S2: Distributions of GFP intensity from HEK293T cells with stably integrated SOD1-GFP indicate reduced abundance for pathogenic SOD1 variants.
Flow cytometric GFP distribution of single, viable cells with integrated SOD1-GFP at the *Bxb1* site in HEK293T cells for pathogenic variants (purple text), and WT SOD1-GFP control and one proxy-benign variant (p.Gly130Ser) taken from gnomAD (green text). Black vertical line indicates potential gating strategy to enrich for variants with high abundance.

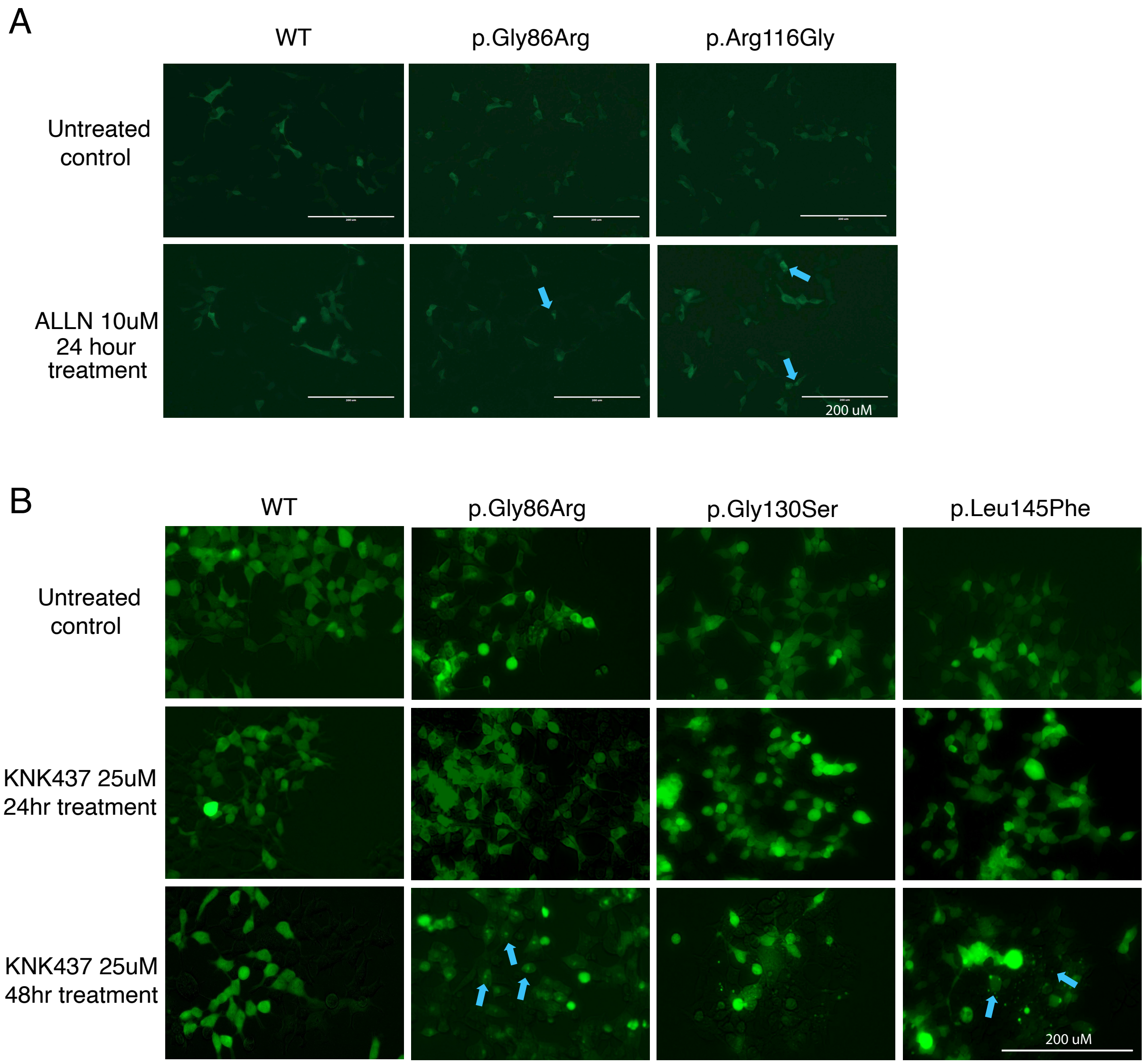

Figure S3: Chemical treatment of HEK293T cells achieved only limited SOD1-GFP aggregation. Aggregates indicated by the blue arrows for: (A) HEK293T cells with SOD1-GFP expressed at high levels via transient transfection, with and without ALLN treatment; (B) HEK293T cells with SOD1-GFP expressed at moderate levels via stable integration in the Bxb1 recombination site, with and without KNK437 treatment.

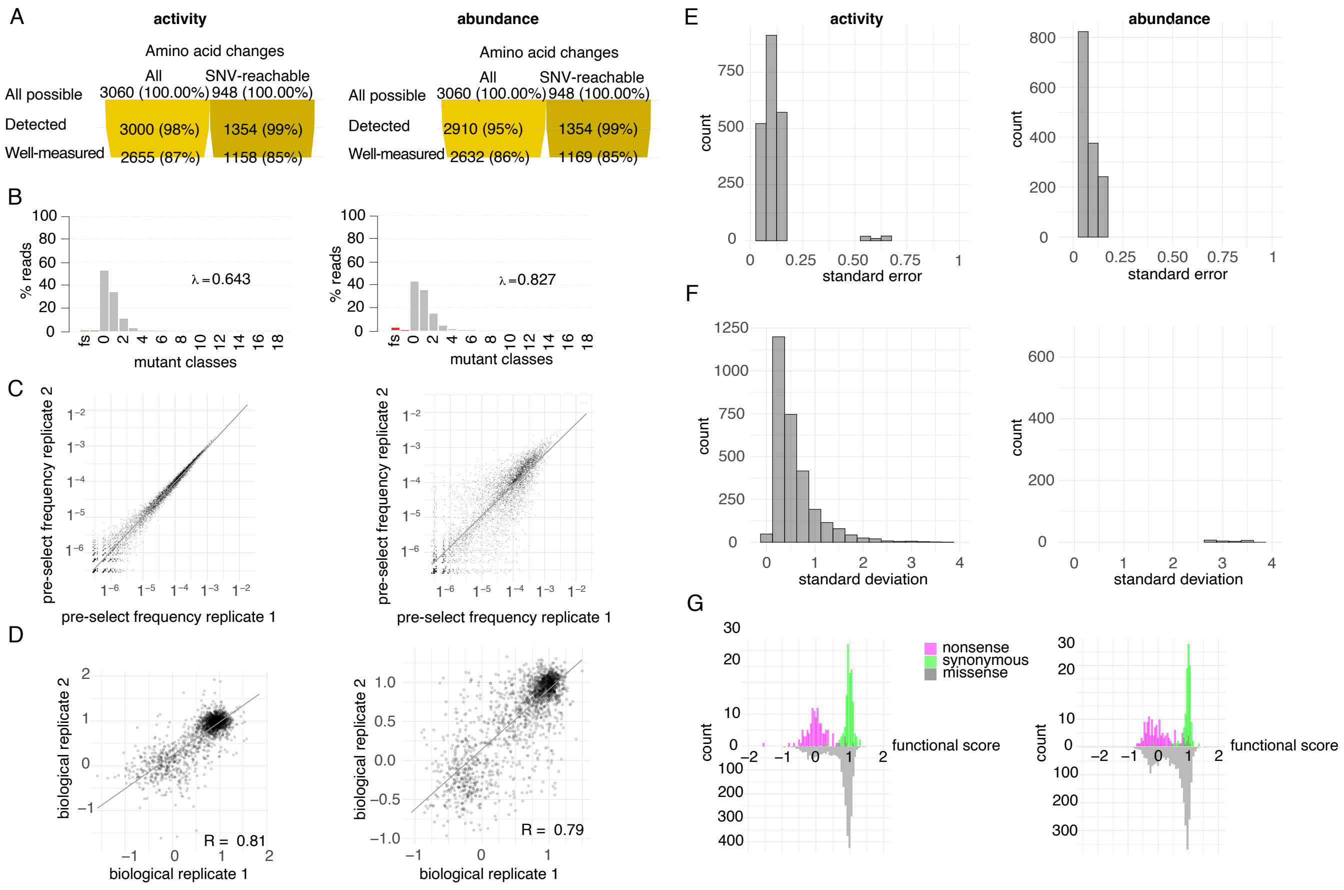

Figure S4: Characterization of SOD1 variant libraries used for activity (left) and abundance (right) variant effect maps.

(A) Percentage of variants detected and passing quality control are shown for each SOD1 map. For each map, the number of synonymous, nonsense, and missense substitutions are shown across all residue positions, both before (left) and after (right) restricting to substitutions that are possible given a single nucleotide change. The three rows correspond to: 1) theoretically possible substitutions; 2) substitutions detected in the pre-selection condition; and 3) substitutions above a threshold pre-selection frequency.

(B) Distribution of the number of missense variants in clones from the total enzymatic activity and abundance SOD1 mutagenized libraries, and the fraction of clones carrying small indels resulting in frameshifts (“fs”). The average number of amino acid changes per clone (λ) is also estimated (see Material and Methods).

(C) Pre-selection variant frequency correlation between biological replicates, for each assay.

(D) Functional score (including missense, nonsense and synonymous variants) correlations between biological replicates, for each assay.

(E) Distributions of standard errors for all missense, nonsense and synonymous variants measured.

(F) Distributions of standard deviations for all missense, nonsense and synonymous variants measured.

(G) Distributions of measured functional impact scores for nonsense (pink), synonymous (green), and missense (gray) variants for the maps.

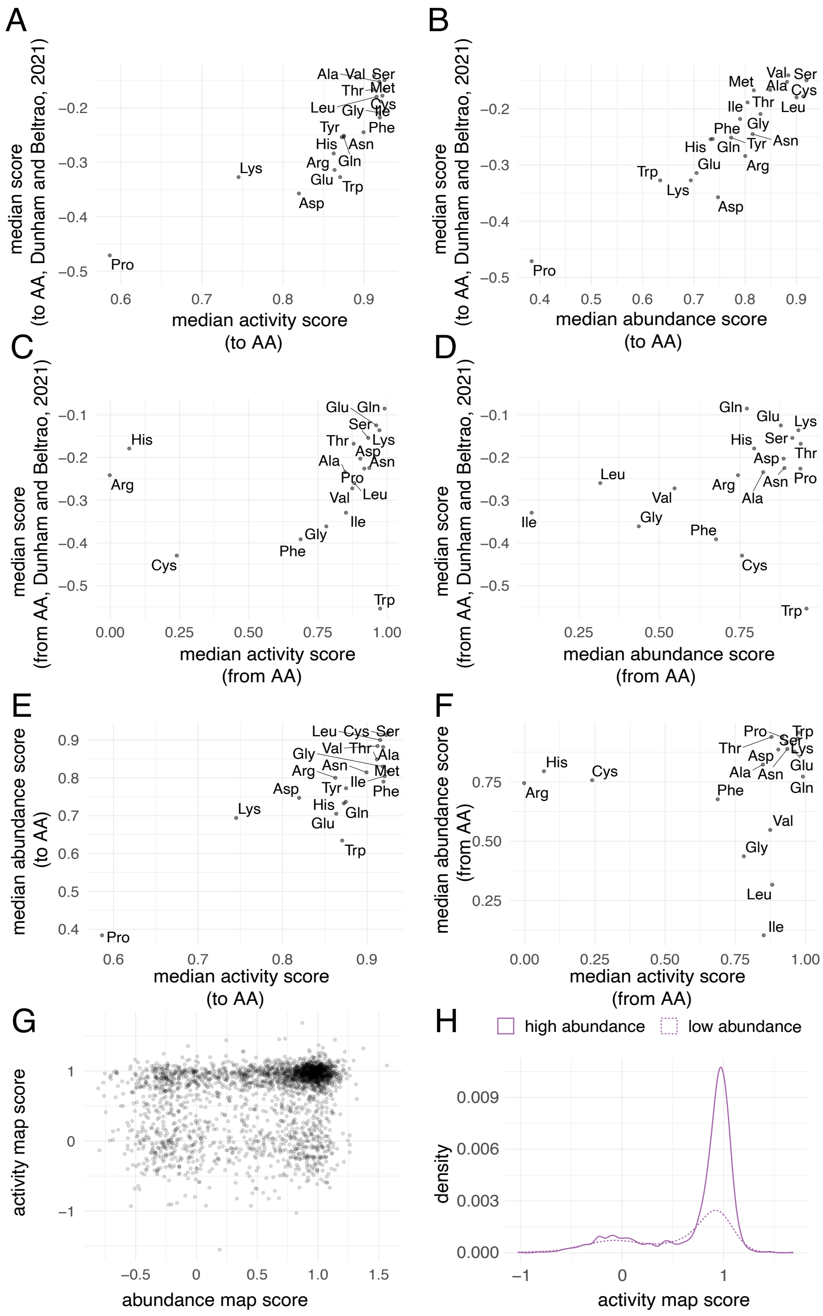

Figure S5: Correspondence between functional scores of SOD1 missense variants from the total enzymatic activity and abundance maps.

Correlation of activity and abundance map functional scores to aggregated scores from substitutions ‘to’ (A-B) and ‘from’ (C-D) amino acids from 28 deep mutational scans[9]. (‘To’ amino acids: Spearman’s R activity = 0.86, p<2e-16. Spearman’s R abundance = 0.90, p<2e-6. ‘From’ amino acids: Spearman’s R activity = 0.48, p=0.045, abundance p>0.05). Correlation of scores corresponding to median score of changes to (E) and from (F) specific amino acids between abundance and activity map (Spearman’s R to amino acid = 0.80, p<4e-5; Spearman’s R from amino acid = 0.56, p<0.02). Correlation between activity and abundance map scores for missense variants only (G; Spearman’s R = 0.25; p<2e-16). Activity scores corresponding to variants with high and low-abundance functional scores (H; abundance score > 0.5, abundance score < 0.5, respectively.) Solid lines represent linear regression fits.

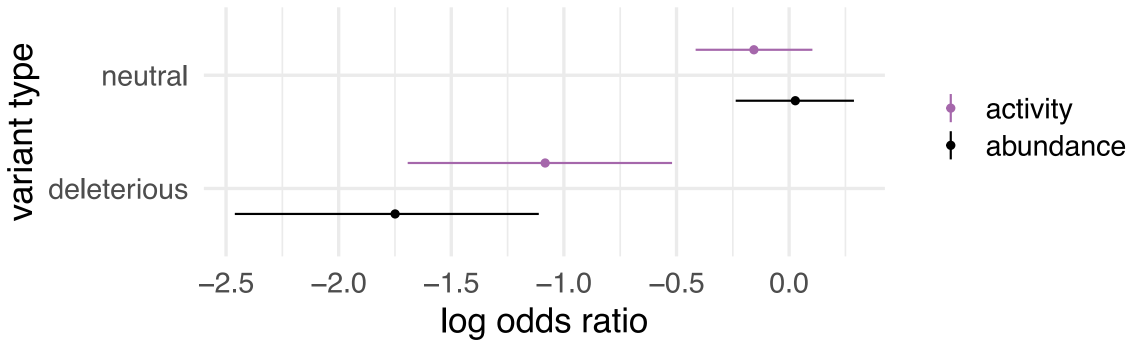

Figure S6: Depletion in human cohorts of missense variants with damaging functional impact scores for activity and abundance assays. Log-odds ratios for the depletion of variants with neutral or damaging scores from total enzymatic activity (purple) or abundance (black) maps in both UK Biobank and gnomAD population sequencing databases. Range lines represent 95% confidence intervals.

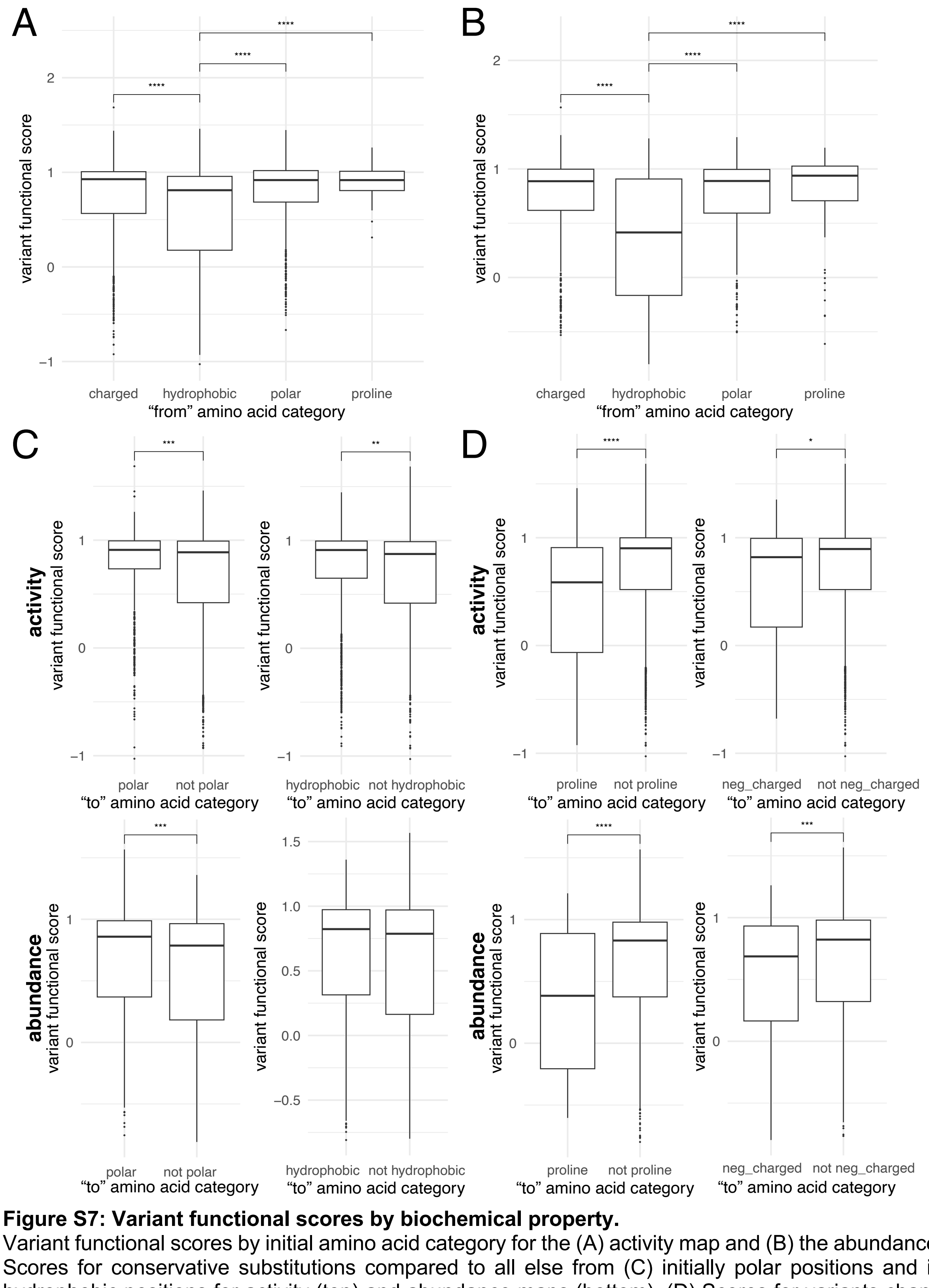

Figure S7: Variant functional scores by biochemical property.

Variant functional scores by initial amino acid category for the (A) activity map and (B) the abundance map. Scores for conservative substitutions compared to all else from (C) initially polar positions and initially hydrophobic positions for activity (top) and abundance maps (bottom). (D) Scores for variants changed to proline, or to negatively charged residues (Asp or Glu) for activity (top) and abundance maps (bottom). p**<0.01, p***<0.001, p****<0.0001 by Wilcoxon.

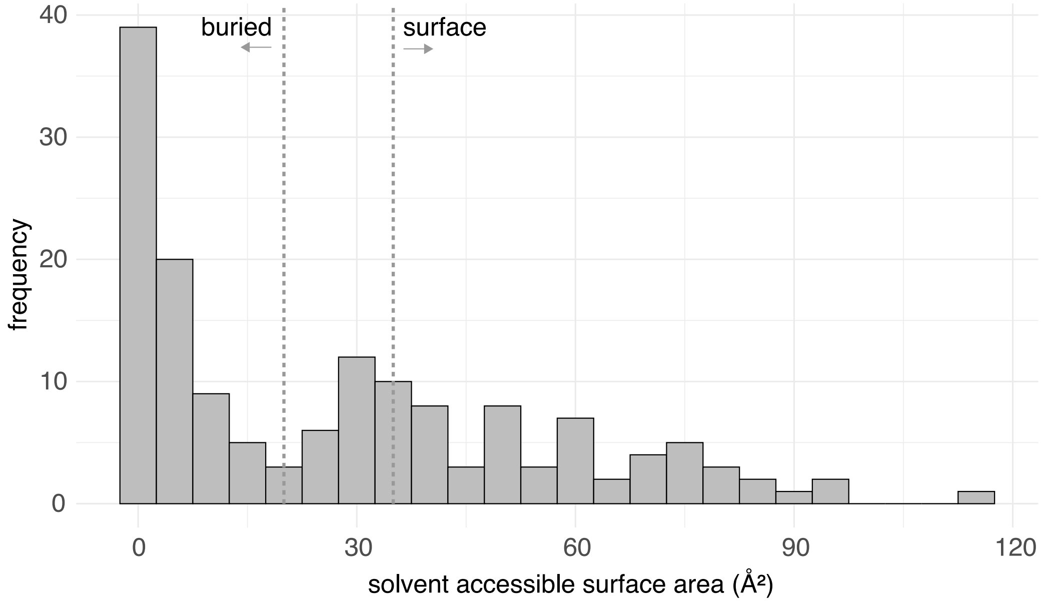

Figure S8: Distribution of solvent accessible surface area for SOD1 residues. SOD1 residues with surface area values exceeding 35% (based on freeSASA values) were considered exposed, while those below 20% were classified as buried (see Materials and Methods).

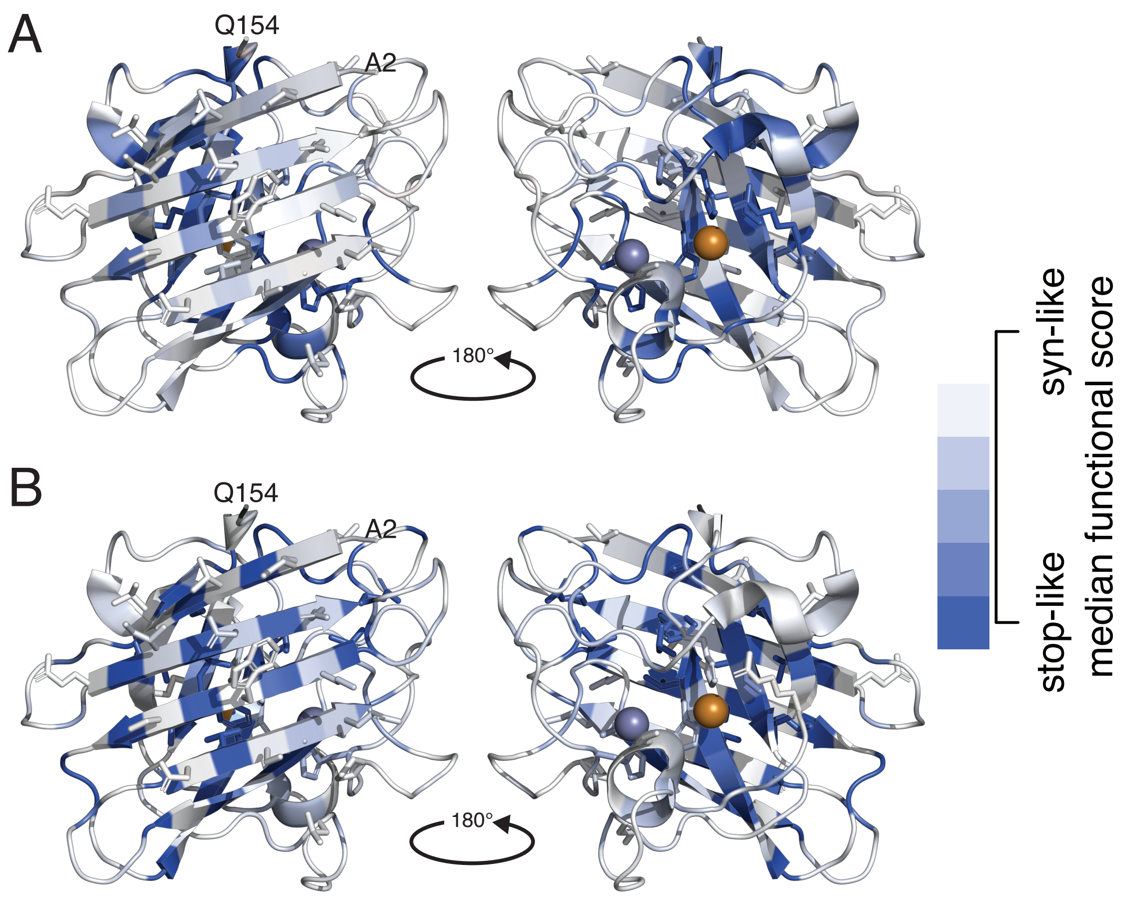

Figure S9: Crystal structure for SOD1 with residues colored according to map scores. Residues in the structure, PDB:1HL5[10] with Cu^{2+} (orange)/Zn^{2+} (blue-grey) ions, were colored based on the median scores of missense variants measured from (A) the enzymatic activity map and (B) the abundance map.

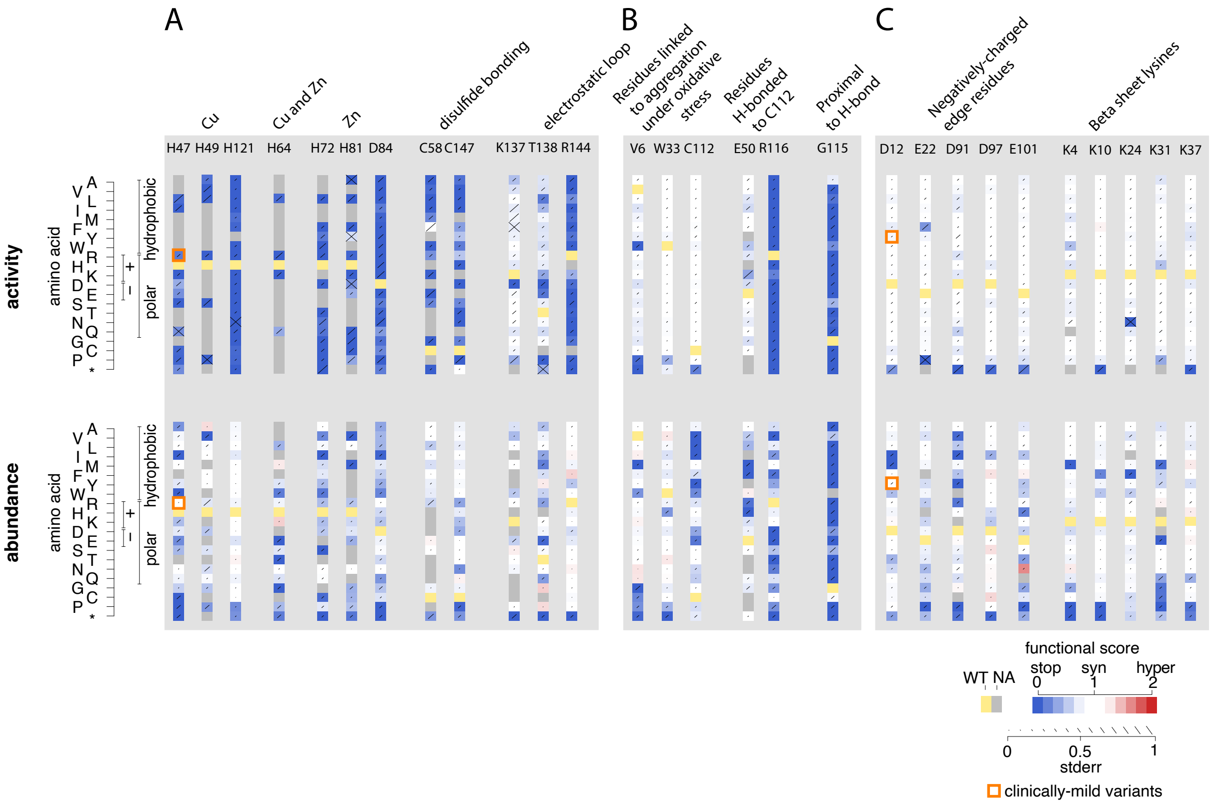

Figure S10: Identifying patterns of mutational tolerance in SOD1.

Functional scores for each possible amino acid substitution (y-axis) at specific SOD1 residue position sets of interest (x-axis). Sets of interest included: (A) residues that participate in Cu2+ and Zn2+-binding, disulphide bonds, or the electrostatic loop that promotes high-affinity site-specific metal binding; (B) residues at which variation has been reported to either cause SOD1 aggregation under oxidative stress, or instability of the H-bond around Cys112; and (C) negatively-charged residues at the edge of the first β-sheet and lysine residues within the first β-sheet. Orange boxes indicate variants with clinically-mild disease presentation.

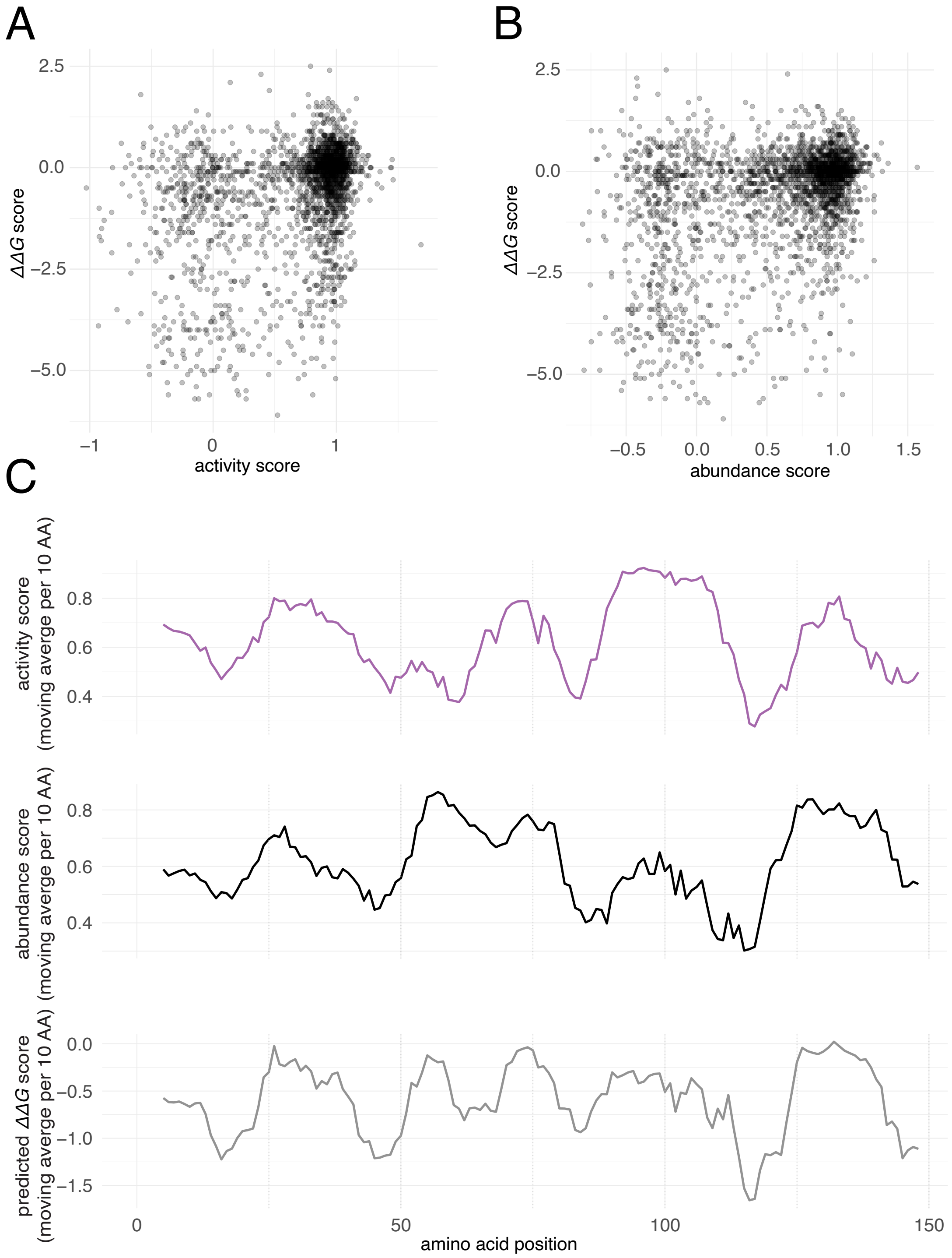

Figure S11: Comparing map scores with predicted effects on protein stability. Functional scores compared to free energy change (*ΔΔG*) for activity (A) and abundance (B) maps. (C) Moving windows of scores from the total enzymatic activity (purple), abundance maps (black), and predicted ΔΔG (light grey) values for SOD1 missense variants at different protein positions. Plotted values represent averages within windows of ten amino acid (AA) positions.

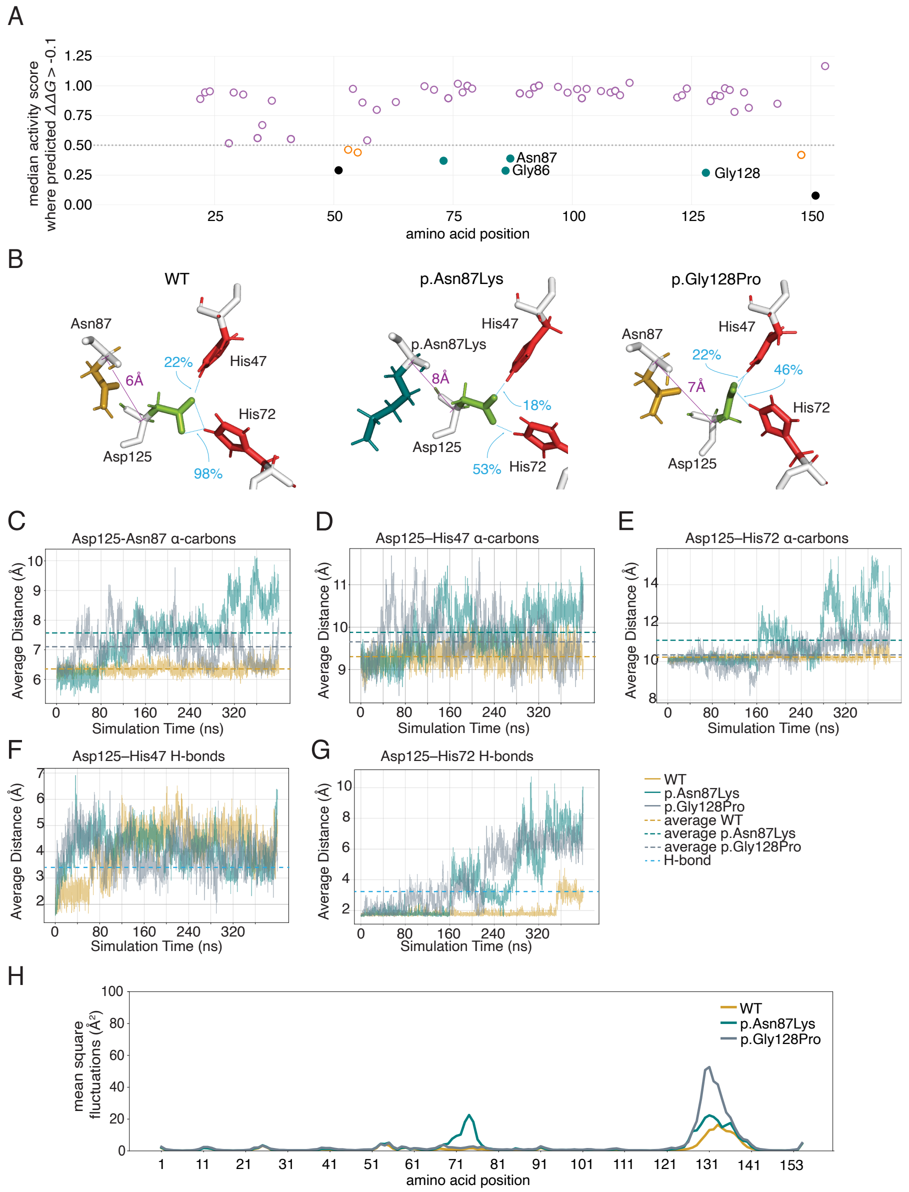

Figure S12: Modeling effects of SOD1 missense variants on protein stability and structure.
(A) For the subset of SOD1 missense variants with no predicted detrimental impact on stability (median predicted *ΔΔG*>-0.1), median total enzymatic activity score is plotted. Circles indicate residues with damaging activity scores: a) at the SOD1-CCS interface (orange); b) residues proximal to the active site ('second-shell residues'; teal); c) at the SOD1 homodimeric interface (black).
(B) To illustrate the structural impact of variants p.Asn87Lys and p.Gly128Pro on the metal-binding site, the average distance (Å) between the Cα atoms of residue pairs 125 (electrostatic loop) and 87 (βstrand residue proximal to the electrostatic loop) is shown in purple, along with the fraction of simulation time (blue) in which hydrogen bonds occurred between metal-binding residue pairs Asp125-His47 and Asp125-His72.
(C-G) Molecular dynamics simulations evaluating impact of variation on inter-residue distances relevant for SOD1's ability to bind metal. The dotted lines represent the average distances between the α-carbons of residue 125 and (C) Asn87, (D) His47, and (E) His72, in WT SOD1, as well as in the p.Asn87Lys and p.Gly128Pro variants. The blue dotted line indicates the hydrogen bond interaction threshold (3.4 Å) for (F) Asp125-His47 and (G) Asp125-His72.
(H) Mean-square fluctuation (MSF) of Cα atoms of WT SOD1, as well as p.Asn87Lys and p.Gly128Pro variants. MSF reflects the average deviation of atoms throughout the simulation (400 ns) relative to the initial structure.

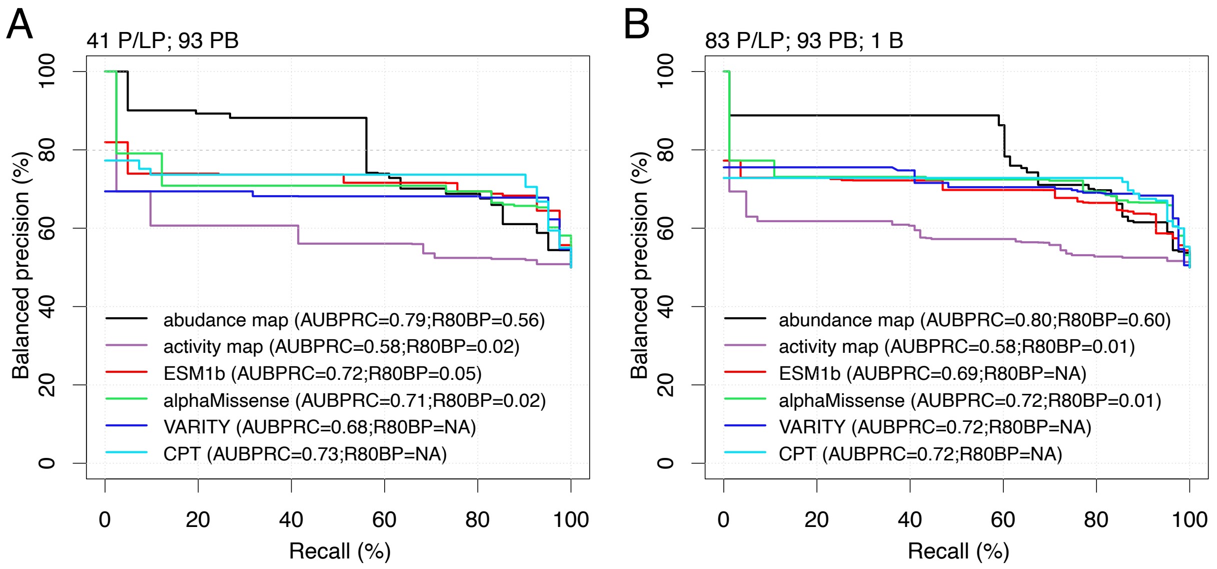

Figure S13: Comparison of total enzymatic activity and abundance SOD1 maps with computational predictors using balanced precision recall analysis for SOD1. We used reference sets drawn from (A) ClinVar/gnomAD and (B) Labcorp/gnomAD to draw PRC curves for the maps and computational predictors. Here we evaluate precision (fraction of variants scoring below each threshold functional impact score that are in the positive reference set containing pathogenic variants) vs recall (fraction of positive reference variants with functional scores below threshold). Precision has been transformed to reflect performance in a balanced test setting where positive and negative sets contain the same number of variants. Balanced precision-recall curves are shown for the total enzymatic activity (purple) and abundance maps (black), as well as computational predictors; ESM1b (red), AlphaMissense (green), VARITY (blue) and CPT (turquoise). Positive and negative reference set sizes (P/LP and B/PB, respectively; see Methods) are indicated. The number of variants in the reference set included in these PRC curves was lower than the curated reference sets due to the intersection between sets for experimental and computational approaches. The variant p.Asn20Ser was classified as B by Labcorp Genetics but was excluded from the ClinVar set because of a conflicting annotation.

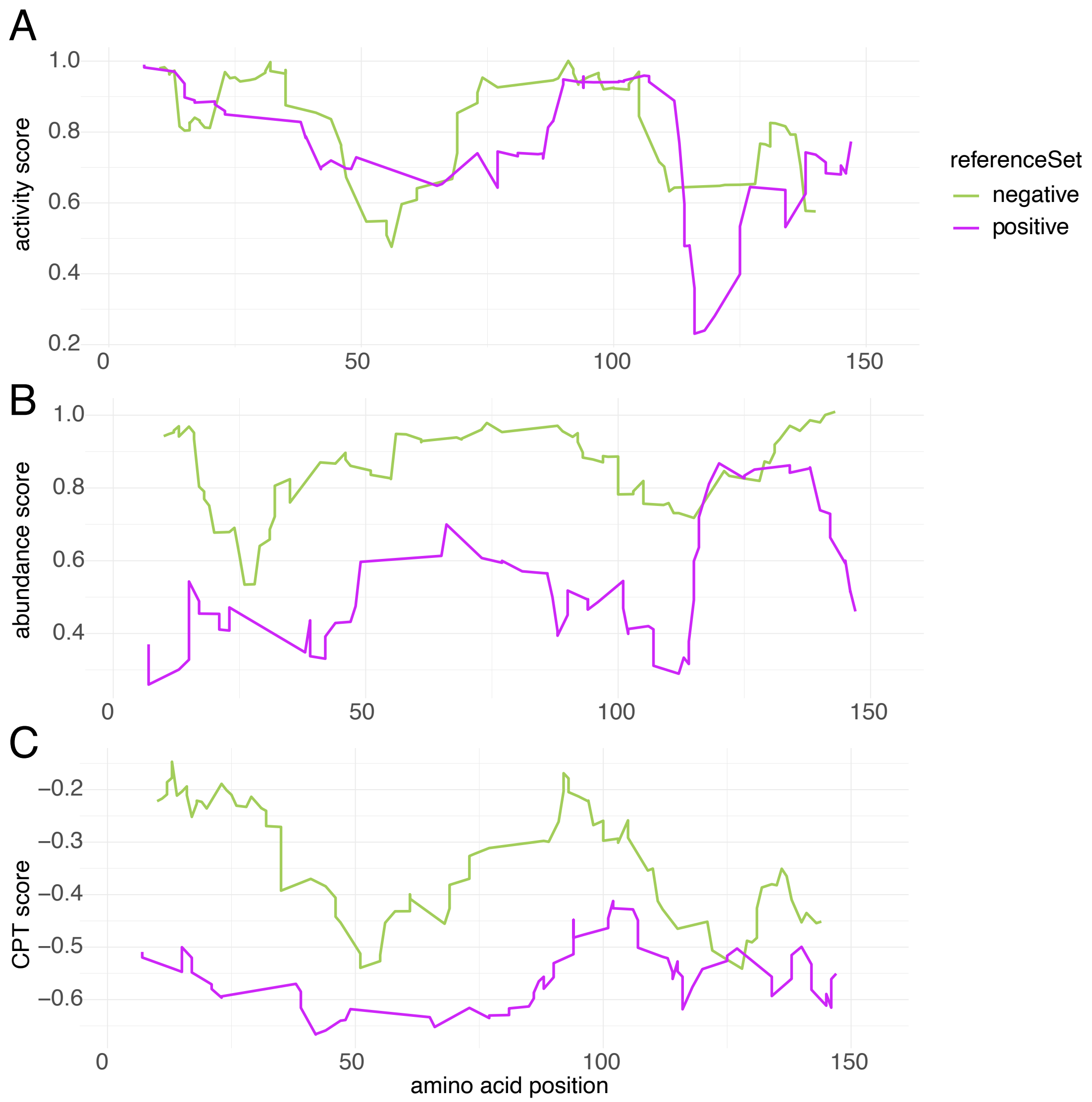

Figure S14: Correspondence of SOD1 maps and computational predictor CPT to clinical variant annotations. Plotted values are (A) activity map, (B) abundance map and (C) computational predictor CPT scores as part of the positive (magenta) or negative (green) reference variant sets from Labcorp/gnomAD, respectively. CPT scores were multiplied by -1 so score directions matched the map scores (more negative considered more damaging).

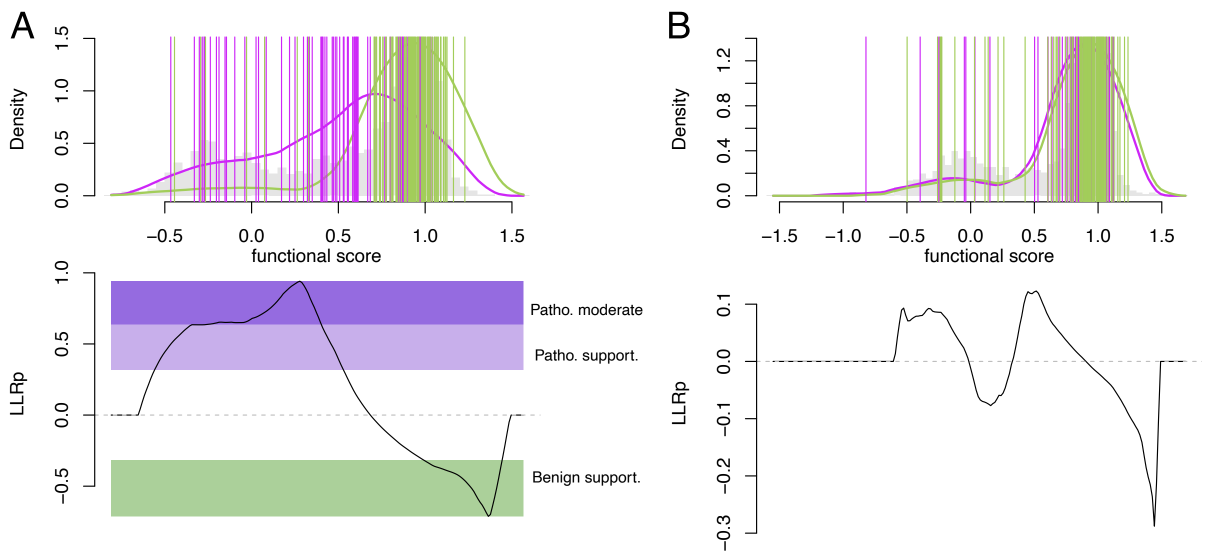

Figure S15: Evidentiary value of abundance map scores for clinical variant interpretation. Calculation and distribution of log-likelihood ratios of pathogenicity (LLRps) for scores of the SOD1 abundance (A) and activity (B) maps. The functions (top) express the log ratio between the likelihood of observing a given score in the score distribution of the Labcorp/gnomAD reference set, including positive reference variants (magenta) from Labcorp Genetics and negative reference set variants from gnomAD + one B from Labcorp (green). LLRps and corresponding evidence strengths are indicated in the bottom panels. Gray histogram bars show the distribution of missense variants for comparison.

Table S1: Summary table of molecular dynamics simulations for residues involved in SOD1 electrostatic loop.

Condition	Position 1	Position 2	Ca Distance (A) between positions 1 and 2	Hydrogen Bonding Time (%) between positions 1 and 2
WT	Asn87	Asp125	6.4 ± 0.2	N/A
WT	His47	Asp125	9.3 ± 0.3	22
WT	His72	Asp125	10.2 ± 0.2	98
p.Asn87Lys	p.Asn87Lys	Asp125	7.6 ± 1.0	N/A
p.Asn87Lys	His47	Asp125	9.9 ± 0.6	18
p.Asn87Lys	His72	Asp125	11.2 ± 1.4	53
p.Gly128Pro	Asn87	Asp125	7.1 ± 0.5	N/A
p.Gly128Pro	His47	Asp125	9.7 ± 0.5	22
p.Gly128Pro	His72	Asp125	10.4 ± 0.6	46

Table S2: Summary table of variant impact on activity or abundance assays for measured variants included in either reference set, with variant-specific information detailed in Table S1.

	**ClinVar/gnomAD Reference Set**		**Labcorp/gnomAD Reference Set**	
	ClinVar P/LP	gnomAD PB	Labcorp P/LP	gnomAD PB, 1 Labcorp B
% damaging (score <0.5) in activity map	5/41<br>12%	12/95<br>13%	10/84<br>12%	12/96<br>13%
% damaging (score <0.5) in abundance map	19/41<br>46%	7/96<br>7%	34/83<br>41%	7/96<br>7%

Table S3: Summary table of the area under the balanced precision-recall curve (AUBPRC) and the recall at 80% balanced precision for maps and computational predictors for the two reference sets tested.

	Reference set			
**Map or computational predictor**	ClinVar/gnomAD: 41 ClinVar P/LP, 93 gnomAD PB		Labcorp/gnomAD**:** 83 Labcorp P/LP, 93 gnomAD PB, 1 Labcorp B	
	**AUBPRC**	**R80BP**	**AUBPRC**	**R80BP**
Abundance map	0.79	56%	0.80	60%
Activity map	0.58	2%	0.58	1%
ESM1b	0.72	5%	0.69	NA
alphaMissense	0.71	2%	0.72	1%
VARITY	0.68	NA	0.72	NA
CPT	0.73	NA	0.72	NA

Supplemental References

1. Proctor, E.A., Fee, L., Tao, Y., Redler, R.L., Fay, J.M., Zhang, Y., Lv, Z., Mercer, I.P., Deshmukh, M., Lyubchenko, Y.L., et al. (2016). Nonnative SOD1 trimer is toxic to motor neurons in a model of amyotrophic lateral sclerosis. Proc. Natl. Acad. Sci. U. S. A. *113*, 614–619.

2. Choi, E.S., and Dokholyan, N.V. (2021). SOD1 oligomers in amyotrophic lateral sclerosis. Curr. Opin. Struct. Biol. *66*, 225–230.

3. Huai, J., and Zhang, Z. (2019). Structural Properties and Interaction Partners of Familial ALS-Associated SOD1 Mutants. Front. Neurol. *10*, 527.

4. Brasil, A. de A., de Carvalho, M.D.C., Gerhardt, E., Queiroz, D.D., Pereira, M.D., Outeiro, T.F., and Eleutherio, E.C.A. (2019). Characterization of the activity, aggregation, and toxicity of heterodimers of WT and ALS-associated mutant Sod1. Proc. Natl. Acad. Sci. U. S. A. *116*, 25991–26000.

5. Hidalgo, I.H., Fleming, T., Eckstein, V., Herzig, S., Nawroth, P.P., and Tyedmers, J. (2016). Characterization of aggregate load and pattern in living yeast cells by flow cytometry. Biotechniques *61*, 137–148.

6. Kim, H.J., Im, W., Kim, S., Kim, S.H., Sung, J.J., Kim, M., and Lee, K.W. (2007). Calcium-influx increases SOD1 aggregates via nitric oxide in cultured motor neurons. Exp. Mol. Med. *39*, 574–582.

7. Lee, H., Radu, C., Han, J.W., and Grailhe, R. (2017). Assay Development for High Content Quantification of Sod1 Mutant Protein Aggregate Formation in Living Cells. J. Vis. Exp. https://doi.org/10.3791/56425.

8. Huang, M., Liu, Y.U., Yao, X., Qin, D., and Su, H. (2024). Variability in SOD1-associated amyotrophic lateral sclerosis: geographic patterns, clinical heterogeneity, molecular alterations, and therapeutic implications. Transl. Neurodegener. *13*, 28.

9. Dunham, A.S., and Beltrao, P. (2021). Exploring amino acid functions in a deep mutational landscape. Mol. Syst. Biol. *17*, e10305.

10. Strange, R.W., Antonyuk, S., Hough, M.A., Doucette, P.A., Rodriguez, J.A., Hart, P.J., Hayward, L.J., Valentine, J.S., and Hasnain, S.S. (2003). The structure of holo and metal-deficient wild-type human Cu, Zn superoxide dismutase and its relevance to familial amyotrophic lateral sclerosis. J. Mol. Biol. *328*, 877–891.
